# Spatiotemporal coordination of cell division and growth during organ morphogenesis

**DOI:** 10.1371/journal.pbio.2005952

**Published:** 2018-11-01

**Authors:** Samantha Fox, Paul Southam, Florent Pantin, Richard Kennaway, Sarah Robinson, Giulia Castorina, Yara E. Sánchez-Corrales, Robert Sablowski, Jordi Chan, Verônica Grieneisen, Athanasius F. M. Marée, J. Andrew Bangham, Enrico Coen

**Affiliations:** 1 Department of Cell and Developmental Biology, John Innes Centre, Norwich, England, United Kingdom; 2 School of Computational Sciences, University of East Anglia, Norwich, England, United Kingdom; 3 Department of Computational and Systems Biology, John Innes Centre, Norwich, England, United Kingdom; University of California San Diego, United States of America

## Abstract

A developing plant organ exhibits complex spatiotemporal patterns of growth, cell division, cell size, cell shape, and organ shape. Explaining these patterns presents a challenge because of their dynamics and cross-correlations, which can make it difficult to disentangle causes from effects. To address these problems, we used live imaging to determine the spatiotemporal patterns of leaf growth and division in different genetic and tissue contexts. In the simplifying background of the *speechless* (*spch*) mutant, which lacks stomatal lineages, the epidermal cell layer exhibits defined patterns of division, cell size, cell shape, and growth along the proximodistal and mediolateral axes. The patterns and correlations are distinctive from those observed in the connected subepidermal layer and also different from the epidermal layer of wild type. Through computational modelling we show that the results can be accounted for by a dual control model in which spatiotemporal control operates on both growth and cell division, with cross-connections between them. The interactions between resulting growth and division patterns lead to a dynamic distributions of cell sizes and shapes within a deforming leaf. By modulating parameters of the model, we illustrate how phenotypes with correlated changes in cell size, cell number, and organ size may be generated. The model thus provides an integrated view of growth and division that can act as a framework for further experimental study.

## Author summary

Organ morphogenesis involves two coordinated processes: growth of tissue and increase in cell number through cell division. Both processes have been analysed individually in many systems and shown to exhibit complex patterns in space and time. However, it is unclear how these patterns of growth and cell division are coordinated in a growing leaf that is undergoing shape changes. We have addressed this problem using live imaging to track growth and cell division in the developing leaf of the mustard plant *Arabidopsis thaliana*. Using subsequent computational modelling, we propose an integrated model of leaf growth and cell division, which generates dynamic distributions of cell size and shape in different tissue layers, closely matching those observed experimentally. A key aspect of the model is dual control of spatiotemporal patterns of growth and cell division parameters. By modulating parameters in the model, we illustrate how phenotypes may correlate with changes in cell size, cell number, and organ size.

## Introduction

The development of an organ from a primordium typically involves two types of processes: increase in cell number through division, and change in tissue shape and size through growth. However, how these processes are coordinated in space and time is unclear. It is possible that spatiotemporal regulation operates through a single control point: either on growth with downstream effects on division, or on division with downstream effects on growth. Alternatively, spatiotemporal regulation could act on both growth and division (dual control), with cross talk between them. Distinguishing between these possibilities is challenging because growth and division typically occur in a context in which the tissue is continually deforming. Moreover, because of the correlations between growth and division it can be hard to distinguish cause from effect [1]. Plant development presents a tractable system for addressing such problems because cell rearrangements make little or no contribution to morphogenesis, simplifying analysis [2].

A growing plant organ can be considered as a deforming mesh of cell walls that yields continuously to cellular turgor pressure [3,4]. In addition to this continuous process of mesh deformation, new walls are introduced through cell division, allowing mesh strength to be maintained and limiting cell size. It is thus convenient to distinguish between the continuous expansion and deformation of the mesh, referred to here as growth, and the more discrete process of introducing new walls causing increasing cell number, cell division [5–8].

The developing *Arabidopsis* leaf has been used as a system for studying cell division control within a growing and deforming tissue. Developmental snapshots of epidermal cells taken at various stages of leaf development reveal a complex pattern of cell sizes and shapes across the leaf, comprising both stomatal and non-stomatal lineages [9]. Cell shape analysis suggests that there is a proximal zone of primary proliferative divisions that is established and then abolished abruptly. Expression analysis of the cell cycle reporter construct *cyclin1 Arabidopsis thaliana* β-glucuronidase (*cyc1At-GUS*) [10] shows that the proximal proliferative zone extends more distally in the subepidermal as compared with the epidermal layer. Analysis of the intensity of *cyc1At-GUS*, which combines both epidermal and subepidermal layers, led to a one-dimensional model in which cell division is restricted to a corridor of fixed length in the proximal region of the leaf [11]. The division corridor is specified by a diffusible factor generated at the leaf base, termed mobile growth factor, controlled by expression of *Arabidopsis* cytochrome P450/*CYP78A5* (*KLUH*). Two-dimensional models have been proposed based on growth and cell division being regulated in parallel by a morphogen generated at the leaf base [12,13]. These models assume either a constant cell area at division, or constant cell cycle duration.

The above models represent important advances in understanding the relationships between growth and division, but leave open many questions, such as the relations of divisions to anisotropic growth, variations along both mediolateral and proximodistal axes, variation between cell layers, variation between genotypes with different division patterns, and predictions in relation to mutants that modify organ size, cell numbers, and cell sizes [14].

Addressing these issues can be greatly assisted through the use of live confocal imaging to directly quantify growth and division [15–22]. Local rates and orientations of growth can be estimated by the rate that landmarks, such as cell vertices, are displaced away from each other. Cell division can be monitored by the appearance of new walls within cells. This approach has been used to measure growth rates and orientations for developing *Arabidopsis* leaves and has led to a tissue-level model for its spatiotemporal control [16]. Live tracking has also been used to follow stomatal lineages and inform hypotheses for stomatal division control [23]. It has also been applied during a late stage of wild-type leaf development after most divisions have ceased [24]. However, this approach has yet to be applied across an entire leaf for extended periods to compare different cell layers and genotypes.

Here, we combine tracking and modelling of 2D growth in different layers of the growing *Arabidopsis* leaf to study how growth and division are integrated during organ morphogenesis. We exploit the *speechless* (*spch*) mutant to allow divisions to be followed in the absence of stomatal lineages, and show how the distribution and rates of growth and cell division vary in the epidermal and subepidermal layers along the proximodistal and mediolateral axes and in time. We further compare these findings to those of wild-type leaves grown under similar conditions. Our results reveal spatiotemporal variation in both growth rates and cell properties, including cell sizes, shapes, and patterns of division. By developing an integrated model of growth and division, we show how these observations can be accounted for by a model in which core components of both growth and division are under spatiotemporal control. Varying parameters of this model illustrates how changes in organ size, cell size, and cell number are likely interdependent, providing a framework for evaluating growth and division mutants.

## Results

To develop an integrated model of growth and division, we first tracked the epidermis of *spch* mutants, as they exhibit a simplified pattern of cell lineages [23]. Cell division dynamics were monitored by measuring spatiotemporal variation in two components: competence and execution. Competence refers to whether a cell has the potential to divide at some point in the future, whereas execution refers to a cell undergoing division (i.e., being cleaved into two).

### A proximal zone of division competence initially extends with tissue growth in the epidermis

**Fig 1:**
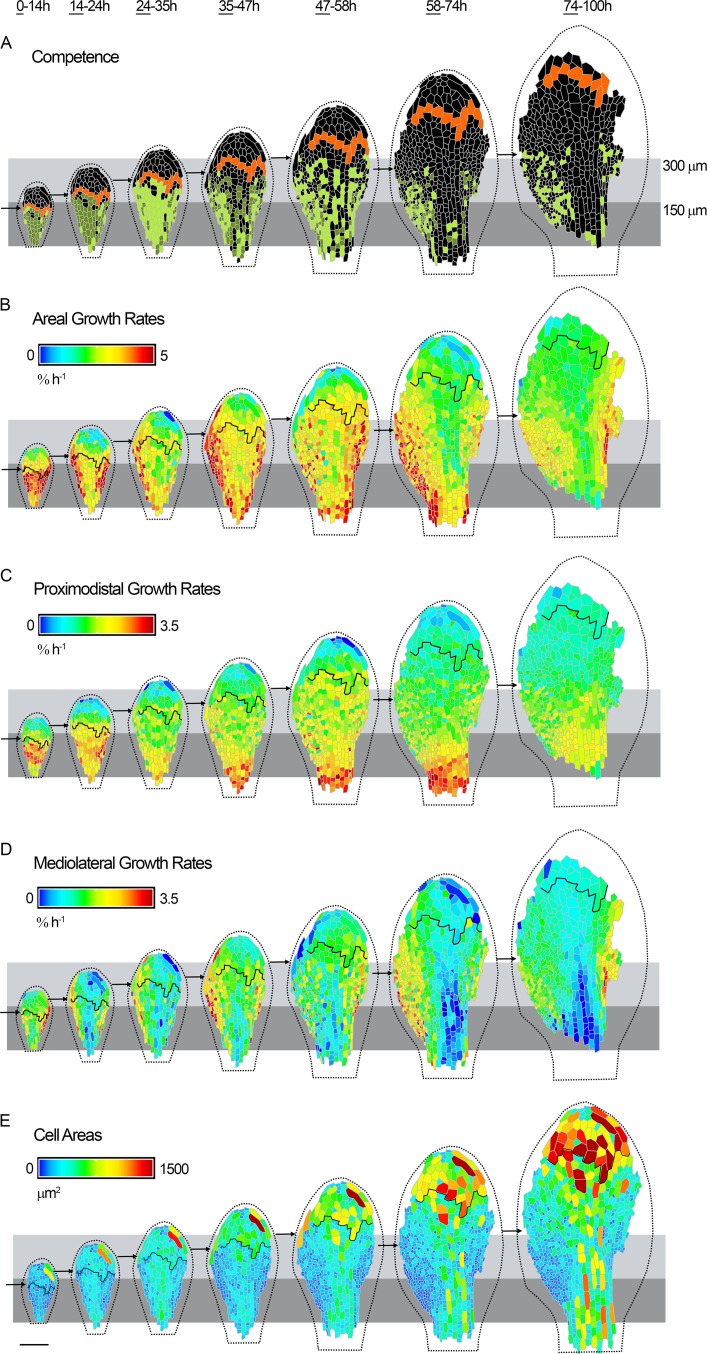
Dynamics of cell division and growth in the *spch* epidermis. Time-lapse imaging of a *spch* leaf at approximately 12-h intervals over 4 d (0–100 h; last time point in series not shown). Data shown on the first time point (underlined) for each tracking interval. Leaf widths for the first time point (left to right) are 0.15, 0.22, 0.27, 0.31, 0.39, 0.48, and 0.68 mm. (**A**) Cells amenable to tracking that were competent to divide (green), and either executed division during the interval (light green) or divided in a later interval (dark green). Cells that did not divide (black, first row in 0–14 h are coloured orange throughout). For the last interval (74–100 h), cell divisions could only be tracked for a subset of cells because of missing data points at 100 h. (**B-D**) Cellular growth rates (heat maps) for each tracking interval. Black line refers to orange cells in (A). (**B**) Areal growth rates. (**C**) Growth rates parallel to the midline (proximodistal). (**D**) Growth rates perpendicular to the midline (mediolateral). (**E**) Cell areas for the first time point of the interval. Leaf outline indicated by dotted black line. The petiole-lamina boundary was defined by selecting a cell from a later stage of development, where the lamina narrows, and then tracing its lineage back to all stages. Grey boxes are aligned to the petiole-lamina boundary and extend to 150 or 300 μm. Black arrows indicate distal boundary of the zone of division competence. Scale bar = 100 μm. See also S1 Fig, S2 Fig, S3 Fig, S4 Fig, S5 Fig, and S6 Fig. Source data are available from https://figshare.com/s/b14c8e6cb1fc5135dd87. *spch*, *speechless*.

Tracking cell vertices on the abaxial epidermis of *spch* seedlings imaged at about 12-h intervals allowed cells at a given developmental stage to be classified into those that would undergo division (competent to divide, green, Fig 1A), and those that did not divide for the remainder of the tracking period (black, Fig 1A). During the first time interval imaged (Fig 1A, 0–14 h), division competence was restricted to the basal half of the leaf, with a distal limit of about 150 μm (all distances are measured relative to the petiole-lamina boundary, Fig 1). To visualise the fate of cells at the distal limit, we identified the first row of nondividing cells (orange) and displayed them in all subsequent images. During the following time intervals, the zone of competence extended together with growth of the tissue to a distance of about 300 μm, after which it remained at this position, while orange boundary cells continued to extend further through growth. Fewer competent cells were observed in the midline region at later stages. Thus, the competence zone shows variation along the proximodistal and mediolateral axes of the leaf, initially extending through growth to a distal limit of about 300 μm and disappearing earlier in the midline region.

### Execution of division exhibits spatiotemporal variation in the epidermis

**Fig 2:**
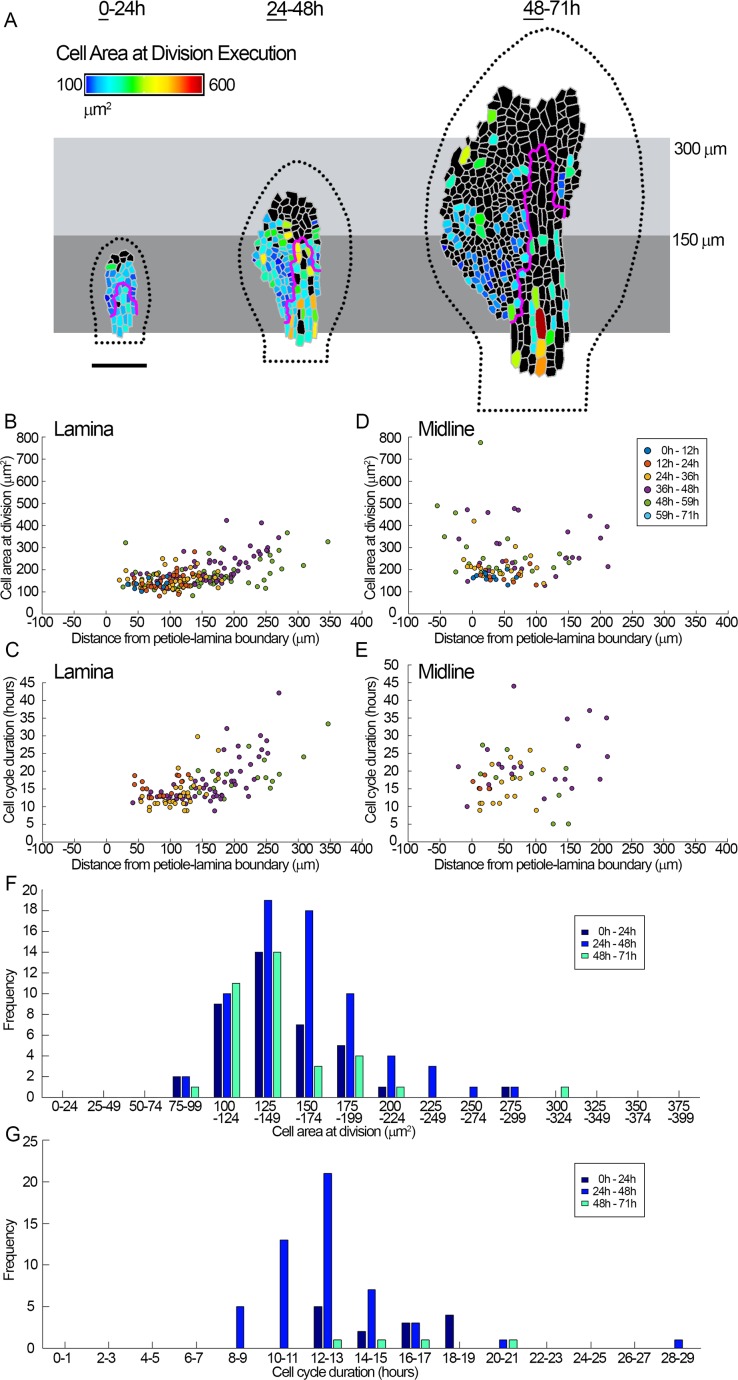
Quantification of cell division execution. Time-lapse imaging of a *spch* leaf imaged at 2-h intervals over 3 d (0–71 h, last time point in series not shown). Leaf widths for the first time point (left to right) are 0.09, 0.21, and 0.36 mm. (**A**) Cell area at division execution (heat map) for cells amenable to tracking, visualised over 24-h intervals and shown on the first time point of the interval (underlined). Cells that did not divide during the 24-h interval are coloured black. If cells divided more than once in the 24-h interval, the area of the first division is shown. Leaf outline indicated by dotted black line. The petiole-lamina boundary was defined as described in Fig 1. Grey boxes are aligned to the petiole-lamina boundary and extend to 150 or 300 *μ*m. Cells within the magenta line were assigned as being within the midline region. (**B-E**) Data grouped into intervals of 12 h (inset in D). Means (*μ*) are shown with ± ranges indicating 1.96 × standard error, corresponding to the 95% confidence limit for a normal distribution. (**B, D**) Area of cells at division execution versus distance from the petiole-lamina boundary. (**B**) Cells in the lamina (*μ* = 170.8 ± 7.7 *μ*m^2^), mean cell area of cells within the proximal 150 *μ*m = 151 ± 6.5 *μ*m^2^, mean cell area of cells outside the proximal 150 *μ*m = 203 ± 9.7 *μ*m^2^. (**D**) Cells in the midline region (*μ* = 243.0 ± 22.4 *μ*m^2^). (**C, E**) Cell cycle duration versus distance from the petiole-lamina boundary. (**C**) Cells in the lamina (*μ* = 16.2 ± 1.0 h). For cells in the proximal 150 *μ*m, *μ* = 13.9 ± 0.8 h. For cells outside the proximal 150 *μ*m, *μ* = 19.4 ± 1.8 h. (**E**) Cells in the midline (*μ* = 18.9 ± 2.1 h). (**F, G**) Histograms of cell area at time of division execution, and cell cycle duration, during time intervals of 24 h (as in A). (**F**) Area at division execution for cells within the proximal 150 *μ*m of the lamina (*μ* = {145.6, 158.3, 142.6}, standard deviation σ = {36.3, 37.9, 40.1}). (**G**) Cell cycle duration for cells within the proximal 150 *μ*m of the lamina (*μ* = {15.8, 13.1, 16.7}, σ = {2.5, 3.3, 2.7}). Measurements of cell area and cell cycle duration are accurate to within 2 h (the time interval between imaging) of division execution. Scale bar = 100 μm. See also S7 Fig. Source data are available from https://figshare.com/s/b14c8e6cb1fc5135dd87. *spch*, *speechless*.

To monitor execution of division, we imaged *spch* leaves at shorter intervals (every 2 h). At early stages, cells executed division when they reached an area of about 150 *μ*m^2^ (Fig 2A, 0–24 h). At later stages, cells in the proximal lamina (within 150 *μ*m) continued to execute division at about this cell area (mean = 151 ± 6.5 *μ*m^2^, Fig 2B), while those in the more distal lamina or in the midline region executed divisions at larger cell areas (mean = 203 ± 9.7 *μ*m^2^ or 243.0 ± 22.4 *μ*m^2^, respectively, Fig 2A, 2B and 2D). Cell cycle duration showed a similar pattern, being lowest within the proximal 150 *μ*m of the lamina (mean = 13.9 ± 0.8 h) and higher distally (mean = 19.4 ± 1.8 h) or in the midline region (18.9 ± 2.1 h, Fig 2C and 2E). Within any given region, there was variation around both the area at time of division execution and the cell cycle duration (Fig 2F and 2G). For example, the area at execution of division within the proximal 150 *μ*m of the lamina had a mean of about 150 *μ*m^2^, with standard deviation of about 40 *μ*m^2^ (Fig 2F). The same region had a cell cycle duration with a mean of about 14 h and a standard deviation of about 3 h. Thus, both the area at which cells execute division and cycle duration show variation around a mean, and the mean varies along the proximodistal and mediolateral axes of the leaf. These findings suggest that models in which either cell area at the time of division or cell cycle duration are fixed would be unable to account for the observed data.

### Epidermal growth rates exhibit spatiotemporal variation

To determine how cell division competence and execution are related to leaf growth, we measured areal growth rates (relative elemental growth rates [25]) for the different time intervals, using cell vertices as landmarks (Fig 1B). Areal growth rates varied along both the mediolateral and proximodistal axis of the leaf, similar to variations observed for competence and execution of division. The spatiotemporal variation in areal growth rate could be decomposed into growth rates in different orientations. Growth rates parallel to the midline showed a proximodistal gradient, decreasing towards the distal leaf tip (Fig 1C and S1A Fig). By contrast, mediolateral growth was highest in the lateral lamina and declined towards the midline, becoming very low there in later stages (Fig 1D and S1B Fig). The region of higher mediolateral growth may correspond to the marginal meristem [26]. Regions of low mediolateral growth (i.e., the proximal midline) showed elongated cell shapes. Models for leaf growth therefore need to account not only for the spatiotemporal pattern of areal growth rates but also the pattern of anisotropy (differential growth in different orientations) and correlated patterns of cell shape.

### Cell sizes reflect the combination of growth and division

**Fig 3:**
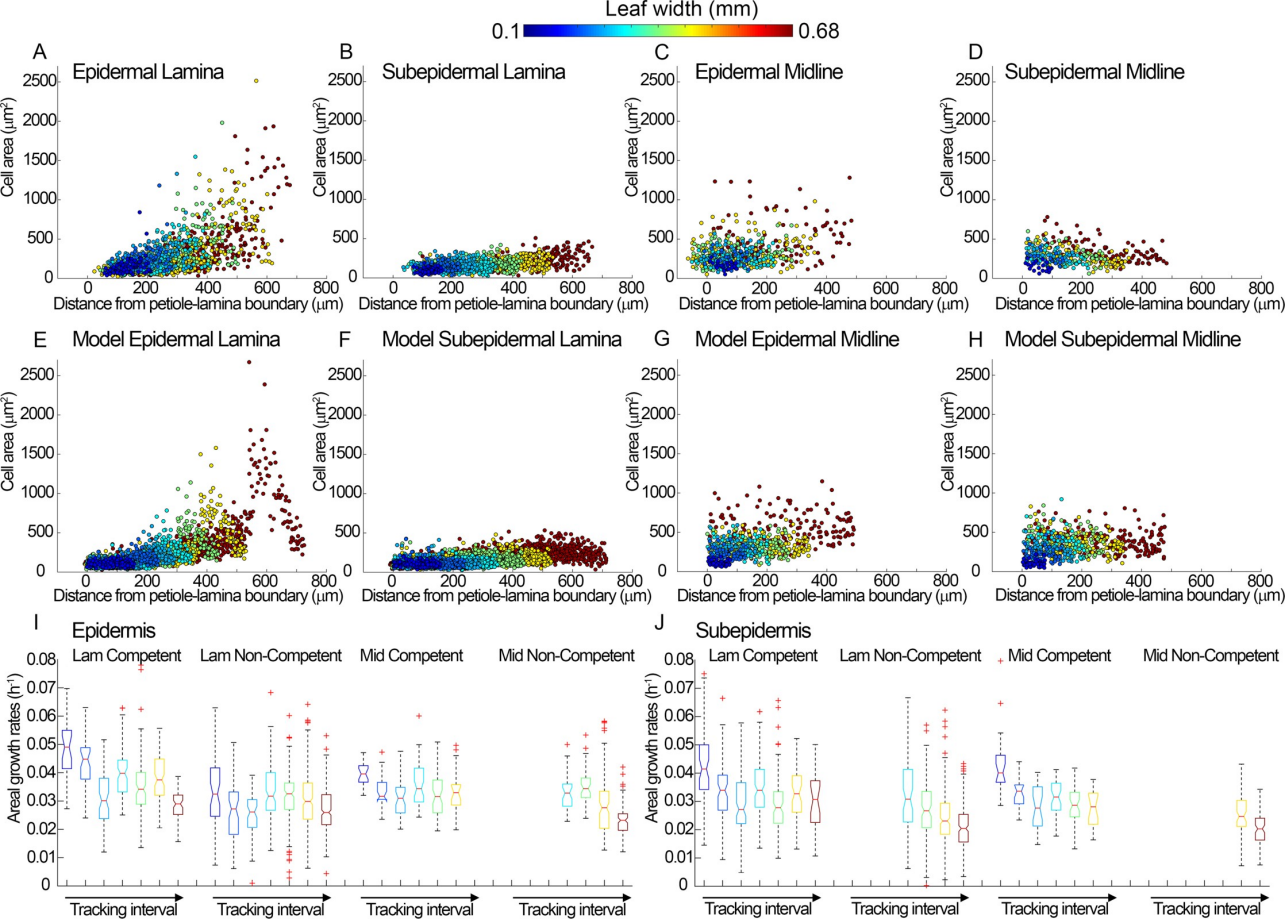
Quantification of cell areas and growth rates in epidermal and subepidermal data and models. (**A-D**) Data from cells amenable to tracking in the time-lapse experiment shown in Fig 1 and Fig 6. Data points are colour coded according to leaf width at the beginning of each time interval, as detailed in legend to Fig 1. (**A**) Epidermal cells in the lamina. (**B**) Subepidermal cells in the lamina. (**C**) Epidermal cells in the midline. (**D**) Subepidermal cells in the midline. (**E-H**) Output from epidermal and subepidermal models. (**E**) v-cells in the lamina of the epidermal model. (**F**) v-cells in the lamina of the subepidermal model. (**G**) v-cells in the midline of the epidermal model. (**H**) v-cells in the midline of the subepidermal model. Model data points are colour coded according to leaf width at equivalent stages to the data. (**I-J**) Areal growth rates of tracked cells in the lamina (Lam) and midline (Mid) regions, according to whether they were competent to divide or not competent to divide for the (**I**) epidermis and (**J**) subepidermis. Data are grouped according to tracking interval (as in Fig 1); colours represent leaf widths at the start of each interval (as for A-D). Boxes represent the central 50% of the data, with top and bottom at the 25% and 75% quantiles of the data. Central red lines represent the median of the data, and two medians are significantly different (at a 5% significance level) if their notches overlap. Outliers are shown as red crosses. Data with a sample size less than 15 are omitted. Source data are available from https://figshare.com/s/b14c8e6cb1fc5135dd87. Lam, lamina; Mid, midline; v-cell, virtual cell.

Cell size should reflect both growth and division: growth increases cell size while division reduces cell size. Cell periclinal areas were estimated from tracked vertices (Fig 1E). Segmenting a sample of cells in 3D showed that these cell areas were a good proxy for cell size, although factors such as leaf curvature introduced some errors (for quantifications see S5 Fig, and ‘Analysis of cell size using 3D segmentation’ in Materials and methods). At the first time point imaged, cell areas were about 100–200 *μ*m^2^ throughout most of the leaf primordium (Fig 1E, left). Cells within the proximal 150 *μ*m of the lamina remained small at later stages, reflecting continued divisions. In the proximal 150–300 *μ*m of the lamina, cells were slightly larger, reflecting larger cell areas at division execution. Lamina cells distal to 300 *μ*m progressively enlarged, reflecting the continued growth of these nondividing cells (Fig 1E and Fig 3A). Cells in the midline region were larger on average than those in the proximal lamina, reflecting execution of division at larger cell areas (Fig 1E and Fig 3C). Thus, noncompetent cells increase in area through growth, while those in the competence zone retain a smaller size, with the smallest cells being found in the most proximal 150 *μ*m of the lateral lamina.

### Correlations between cell size and growth rate

**Fig 4:**
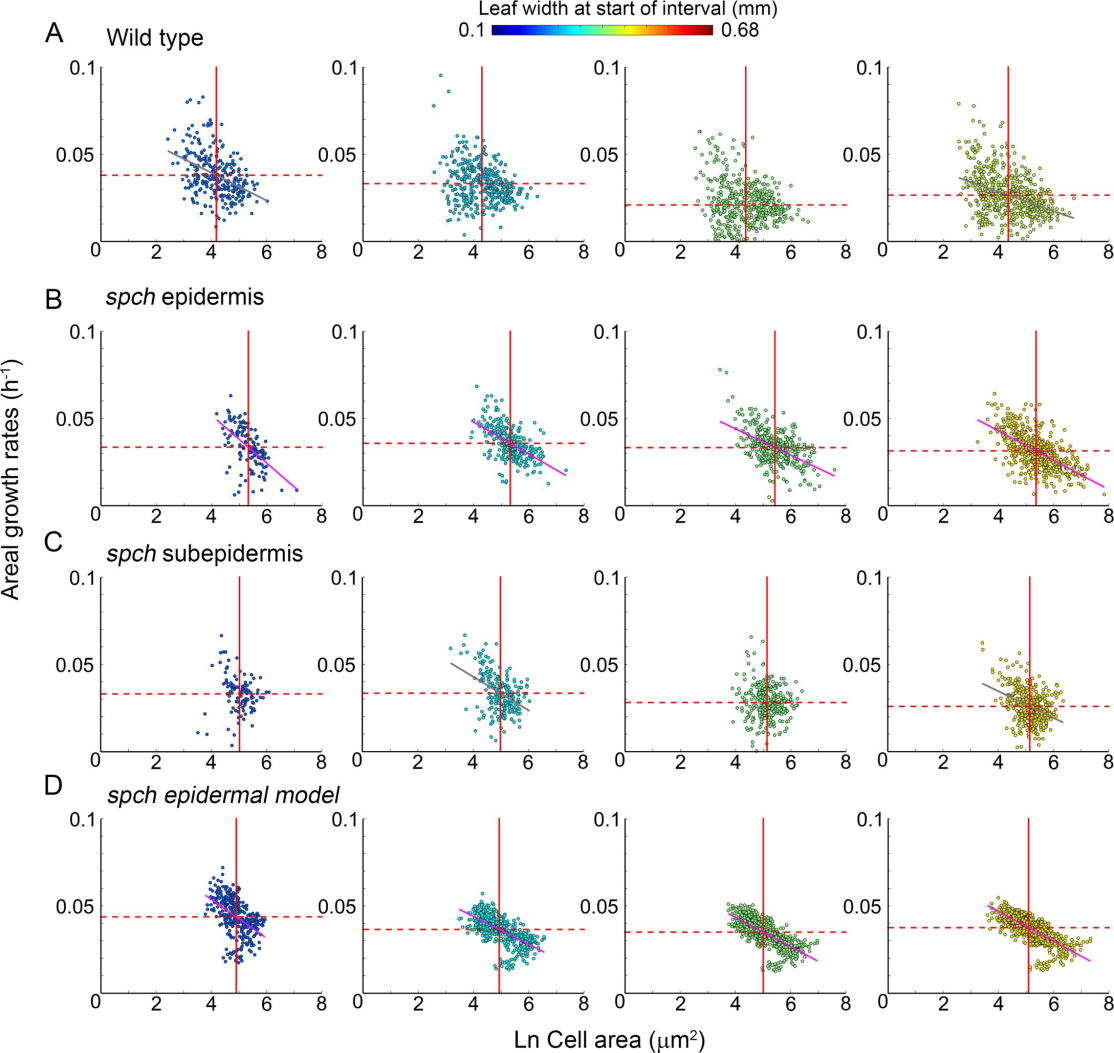
Correlations between growth rates and cell size. Areal cell growth rates against log cell area for time intervals at different developmental stages. Data points are colour coded according to leaf width at the start of the interval (colour scale is shown at the top). Solid red lines indicate mean cell areas (μ*a*) and dashed red lines indicate mean growth rate (μ*k*), with ± ranges indicating 1.96 × standard error of mean. Lines fitted for *R*^2^ > 0.2 are shown in magenta; those with *R*^2^ < 0.2 and > 0.1 are shown in grey (*p*-values are all less than 5.1×10^−6^ for line fits). (**A**) Wild type (from dataset shown in Fig 7B–7D, time intervals: 12–25 h, 25–37 h, 37–47 h, 47–57 h). From left to right, μ*a* = {4.2 ± 0.08, 4.3 ± 0.07, 4.4 ± 0.07, 4.4 ± 0.07}, μ*k* = {0.038 ± 0.001, 0.033 ± 0.001, 0.021 ± 0.001, 0.026 ± 0.001}, gradient *m* = {−0.008, −0.004, −0.002, −0.006}, and *R*^2^ = {0.18,−,−,0.14}. (**B**) *spch* epidermis (from dataset shown in Fig 1, time intervals: 14–24 h, 35–47 h, 47–58 h, 58–74 h), μ*a* = {5.3 ± 0.08, 5.3 ± 0.06, 5.4 ± 0.06, 5.4 ± 0.06}, μ*k* = {0.033 ± 0.001, 0.036 ± 0.001, 0.033 ± 9×10^−4^, 0.031 ± 8×10^−4^}, *m* = {−0.014, −0.009, −0.008, −0.008}, and *R*^2^ = {0.31,0.31,0.27,0.38}. (**C**) *spch* subepidermis (from dataset shown in Fig 6, time intervals: 14–24 h, 35–47 h, 47–58 h, 58–74 h), μ*a* = {5.0 ± 0.09, 5.0 ± 0.06, 5.1 ± 0.05, 5.1 ± 0.05}, μ*k* = {0.033 ± 0.002, 0.033 ± 0.001, 0.028 ± 0.001, 0.026 ± 9×10^−4^}, *m* = {−0.003, −0.010, −0.002, −0.008}, and *R*^2^ = {−, 0.19, −, 0.15}. (**D**) *spch* epidermal model output at stages corresponding to leaf widths in (B), μ*a* = {4.9 ± 0.05, 4.9 ± 0.05, 5.0 ± 0.04, 5.0 ± 0.04}, μ*k* = {0.045 ± 0.001, 0.037 ± 5×10^−4^, 0.035 ± 0.004, 0.038 ± 1×10^−4^}, *m* = {−0.012, −0.008, −0.008, −0.009}, *R*^2^ = {0.33,0.42,0.54,0.59}. Source data are available from https://figshare.com/s/b14c8e6cb1fc5135dd87. Ln, natural logarithm; *spch*, *speechless*.

Visual comparison between areal growth rates (Fig 2B) with cell sizes (Fig 2E) suggested that regions with higher growth rates had smaller cell sizes. Plotting areal growth rates against log cell area confirmed this impression, revealing a negative correlation between growth rate and cell size (Fig 4B). Thus, rapidly growing regions tend to undergo more divisions. This relationship is reflected in the pattern of division competence: mean areal growth rates of competent cells in the lamina were higher than noncompetent cells, particularly at early stages (Fig 3I). However, there was no fixed threshold growth rate above which cells were competent, and for the midline region there was no clear difference between growth rates of competent and noncompetent cells (Fig 3I). Plotting areal growth rates for competent and noncompetent cells showed considerable overlap (S6 Fig), with no obvious switch in growth rate when cells no longer divide (become noncompetent). Thus, high growth rate broadly correlates with division competence, but the relationship is not constant for different regions or times.

### Subepidermal division rules can be decoupled from those of the overlying epidermis

To determine how the patterns and correlations observed for the epidermis compared to those in other tissues, we analysed growth and divisions in the subepidermis. The advantage of analysing an adjacent connected cell layer is that unless intercellular spaces become very large, the planar cellular growth rates will be very similar to those of the attached epidermis (because of tissue connectivity and lack of cell movement). Comparing the epidermal and subepidermal layers therefore provides a useful system for analysing division behaviours in a similar spatiotemporal growth context. Moreover, by using the *spch* mutant, one of the major distinctions in division properties between these layers (the presence of stomatal lineages in the epidermis) is eliminated.

**Fig 5:**
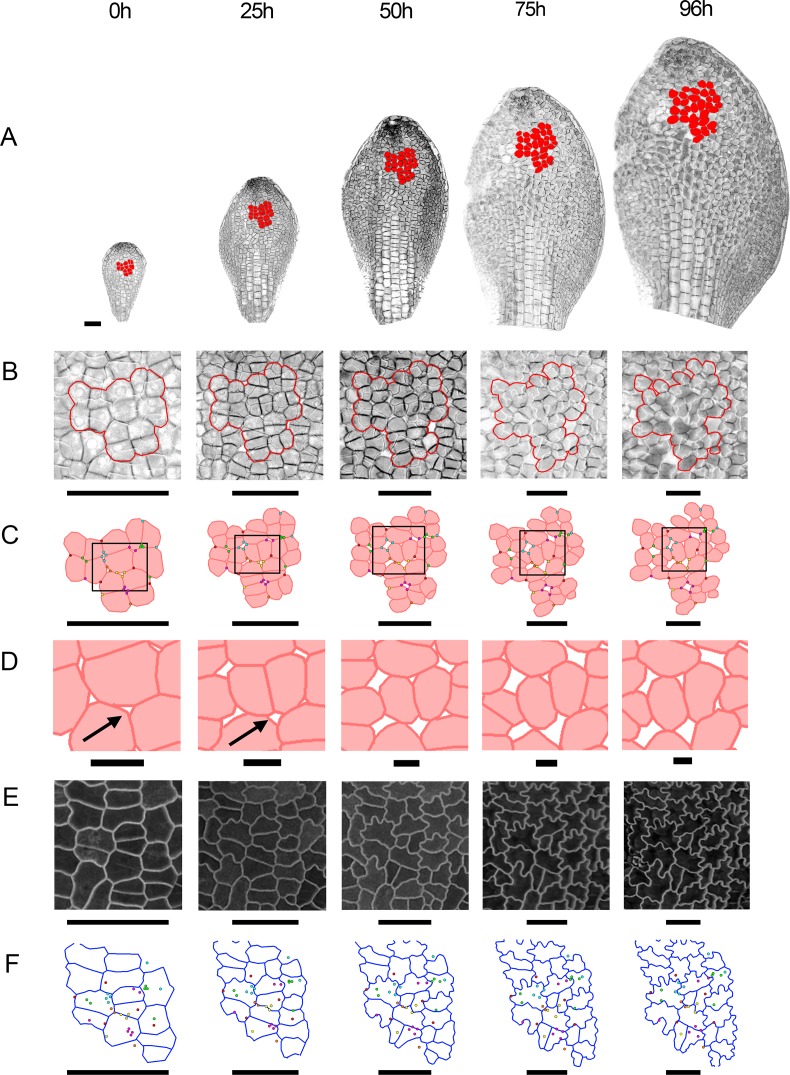
Subepidermal and epidermal cells in a *spch* leaf. (**A**) Projections of the subepidermal layer, imaged approximately every 24 h over 4 d (0–96 h). Cell divisions and growth for the epidermal layer of this leaf are shown in S3 Fig. Leaf widths (left to right) are 0.17, 0.27, 0.39, 0.50, and 0.58 mm. A patch of cells was tracked between intervals (cells coloured red). (**B**) Enlargement of the patch of cells in (A) (red outline). (**C**) Cells outlined in (B), showing individual cells (filled pink, outlined red) and vertices around some air spaces (coloured dots, also shown in F). (**D**) Enlargement of cells located in the black box of C, showing air spaces (white, examples highlighted with black arrows). (**E**) Epidermal cells adjacent to the subepidermal patch (B). (**F**) Outlines of epidermal cells (blue) with vertices of subepidermal cells (coloured spots, also shown in C). Scale bars for A, B, C, E, F = 50 μm; scale bar for D = 10 μm. See also S8 Fig and S9 Fig. Source data are available from https://figshare.com/s/b14c8e6cb1fc5135dd87. *spch*, *speechless*.

Divisions in the abaxial subepidermis were tracked by digitally removing the overlying epidermal signal (the distalmost subepidermal cells could not be clearly resolved). As with the epidermis, 3D segmentation showed that cell areas were a good proxy for cell size, although average cell thickness was greater (S11 Fig, see also ‘Analysis of cell size using 3D segmentation’ in Materials and methods). Unlike the epidermis, intercellular spaces were observed for the subepidermis. As the tissue grew, subepidermal spaces grew and new spaces formed (Fig 5A–5D). Similar intercellular spaces were observed in subepidermal layers of wild-type leaves, showing they were not specific to *spch* mutants (S8 Fig).

Vertices and intercellular spaces in the subepidermis broadly maintained their spatial relationships with the epidermal vertices (Fig 5C, 5E and 5F). Comparing the cellular growth rates in the plane for a patch of subepidermis with the adjacent epidermis showed that they were similar (S9 Fig), although the subepidermal rates were slightly lower because of the intercellular spaces. This correlation is expected, because unless the intercellular spaces become very large, the areal growth rates of the epidermal and subepidermal layers are necessarily similar.

**Fig 6:**
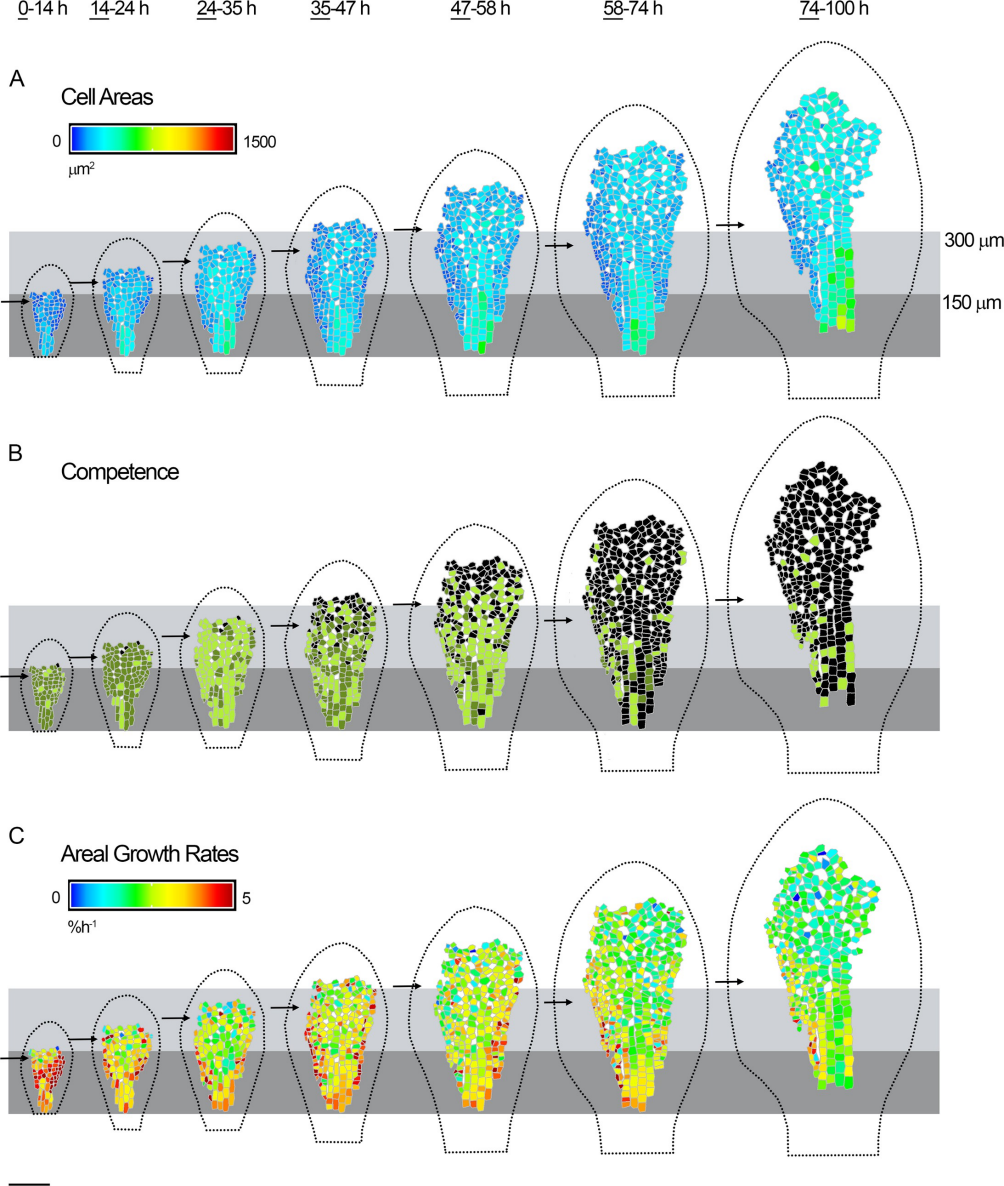
Dynamics of cell division and growth in the *spch* subepidermis. Cells amenable to tracking in the subepidermal layer of the *spch* leaf shown in Fig 1. Data shown on first time point (underlined) for each tracking interval. (**A**) Cell areas (heat map). (**B**) Cells that were competent to divide (green) and either executed division during the interval (light green) or divided in a later interval (dark green). Cells that did not divide (black), intercellular air spaces (white). (**C**) Cellular areal growth rates (heat map). Leaf outline indicated by dotted black line. The petiole-lamina boundary was defined as described in Fig 1. Grey boxes are aligned to the petiole-lamina boundaries and extend to 150 or 300 *μ*m. Black arrows indicate the distal boundary of the epidermal zone of division (as shown in Fig 1). Scale bar = 100 *μ*m. See also S10 Fig and S11 Fig. Source data are available from https://figshare.com/s/b14c8e6cb1fc5135dd87. *spch*, *speechless*.

The most striking difference between subepidermal and epidermal datasets was the smaller size of the distal lamina cells of the subepidermis (compare Fig 6A with Fig 1E, and Fig 3A with Fig 3B). For the epidermis, these cells attain areas of about 1,000 *μ*m^2^ at later stages, while for the subepidermis they remain below 500 *μ*m^2^. This finding was consistent with the subepidermal division competence zone extending more distally (Fig 6B), reaching a distal limit of about 400 μm compared with 300 μm for the epidermis. A more distal limit for the subepidermis has also been observed for cell cycle gene expression in wild type [10]. Moreover, at early stages, divisions occurred throughout the subepidermis rather than being largely proximal, as observed in the epidermis, further contributing to the smaller size of distal subepidermal cells (S10 Fig). Despite these differences in cell size between layers, subepidermal cell areal growth rates showed similar spatiotemporal patterns to those of the overlying epidermis, as expected because of tissue connectivity (compare Fig 6C with Fig 1B). Consequently, correlations between growth rate and cell size were much lower for the subepidermis than for the epidermis (Fig 4B and 4C).

This difference in the relationship between growth and cell size in different cell layers was confirmed through analysis of cell division competence. In the subepidermis, at early stages there was no clear difference between mean growth rates for competent and noncompetent cells (Fig 3J cyan, green), in contrast to what is observed in the epidermis (Fig 3I cyan, green), while at later stages noncompetent cells had a slightly lower growth rate (Fig 3J yellow, red).

### SPCH promotes division competence

**Fig 7:**
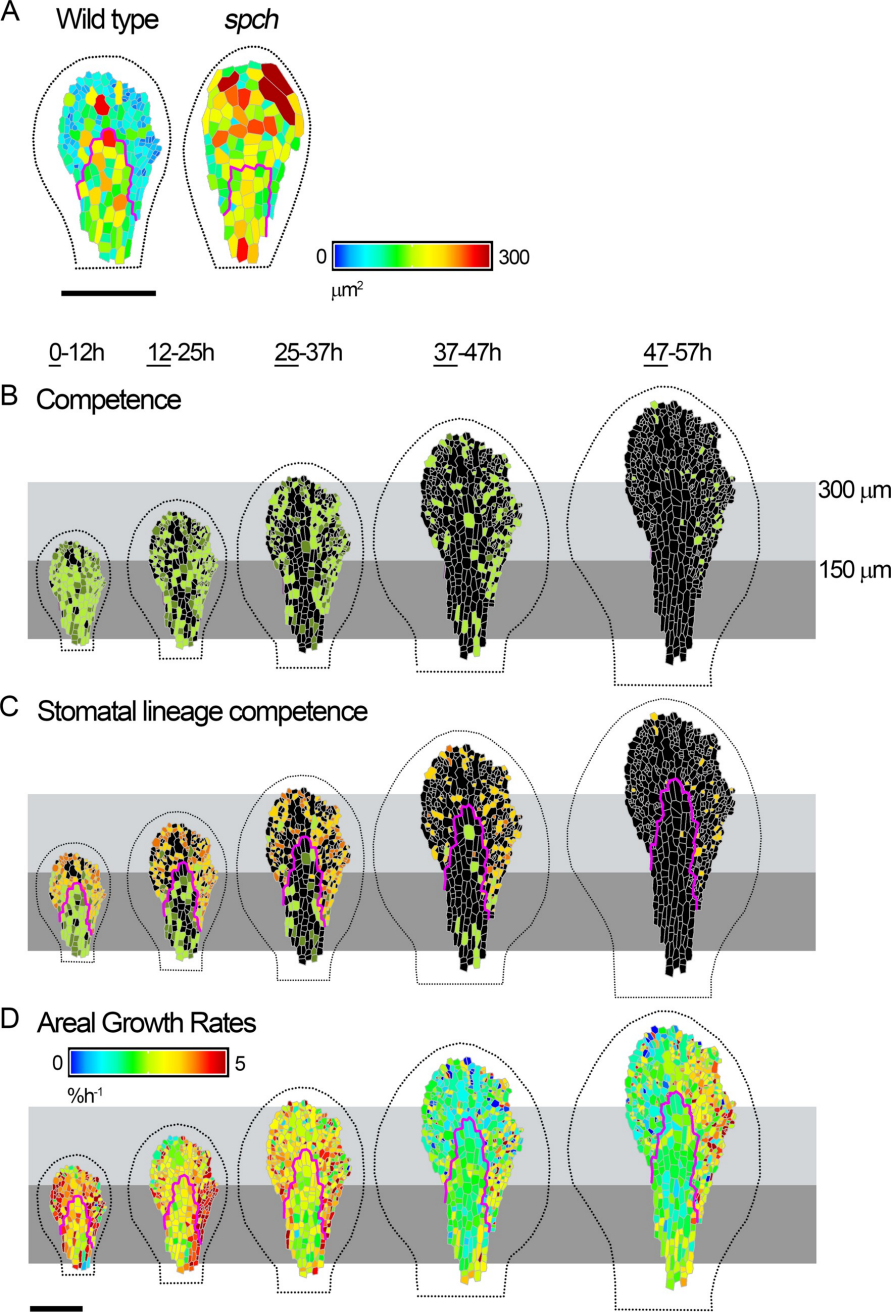
Dynamics of cell division and growth in the wild-type epidermis. (**A**) Cell areas (heat map) of wild-type (left) and *spch* (right) leaves at similar developmental stages. (**B-D**) Cells amenable to tracking from time-lapse imaging of a wild-type leaf (expressing *pSPCH*:*SPCH-GFP*, not shown) at approximately 1-h intervals over 2.5 d (0–57 h, last time point in series not shown). Data are visualised over about 12-h intervals and shown on first time point (underlined) for each interval. Leaf widths for first time point (left to right) are 0.17, 0.23, 0.28, 0.39, and 0.42 mm. (**B**) Cells amenable to tracking that were competent to divide (green) and either executed division during the interval (light green) or divided in a later interval (dark green). Cells that did not divide (black). (**C**) Non-stomatal divisions coloured as for (B). Stomatal lineage divisions that executed division during the interval (yellow) or divided in a later interval (orange). (**D**) Cellular areal growth rates (heat map) for each tracking interval. Leaf outline indicated by dotted black line. The petiole-lamina boundary was defined as described in Fig 1. Grey boxes are aligned to the petiole-lamina boundary and extend to 150 or 300 *μ*m. Cells within the magenta lines were assigned as being destined to form the midline according to their position and shape in the final image. Scale bars = 100 *μ*m. See also S12 Fig, S13 Fig, and S14 Fig. Source data are available from https://figshare.com/s/b14c8e6cb1fc5135dd87. GFP, green fluorescent protein; *spch*, speechless.

To determine how the patterns of growth and division observed in *spch* related to those in wild type, we imaged a line generated by crossing a *spch* mutant rescued by a functional SPCH protein fusion (*pSPCH*:*SPCH-GFP*) to wild type expressing the *PIN3* auxin transporter (*PIN3*:*PIN3-GFP)*, which marks cell membranes in the epidermis [23]. The resulting line allows stomatal lineage divisions to be discriminated from non-stomatal divisions (see below) in a *SPCH* context. At early stages, wild-type and *spch* leaves were not readily distinguishable based on cell size (S12 Fig). However, by the time leaf primordia attained a width of about 150 *μ*m, the number and size of cells differed dramatically. Cell areas in wild type were smaller in regions outside the midline region, compared with corresponding cells in *spch* (Fig 7A). Moreover, cell divisions in wild type were observed throughout the lamina that was amenable to tracking (Fig 7B, 0–12 h), rather than being largely proximal. Divisions were observed over the entire lamina for subsequent time intervals, including regions distal to 300 *μ*m (Fig 7B, 12–57 h). These results indicate that SPCH can confer division competence in epidermal cells outside the proximal zone observed in *spch* mutants.

### SPCH acts autonomously to reduce the cell area at which cells execute division

To further clarify how SPCH influences cell division, we used SPCH-GFP signal to classify wild-type cells into two types: (1) Stomatal lineage divisions, which include both amplifying divisions (cells express SPCH strongly around the time of division and retain expression in one of the daughter cells) (S1 Video, orange/yellow in Fig 7C) and guard mother cell divisions (SPCH expression is bright and diffuse during the first hours of the cycle, transiently switched on around time of division, and then switched off in both daughters). (2) Non-stomatal divisions, in which SPCH expression is much weaker, or only lasts <2 h, and switches off in both daughter cells (S2 Video, light/dark green in Fig 7C).

If cells with inactive SPCH behave in a similar way in wild-type or *spch* mutant contexts, we would expect non-stomatal divisions to show similar properties to divisions in the *spch* mutant. In the first time interval, non-stomatal divisions (green) were observed within the proximal 150 *μ*m (Fig 7C, 0–12 h), similar to the extent of the competence zone in *spch* (Fig 1A, 0–14h). The zone of non-stomatal divisions then extended to about 250 *μ*m and became restricted to the midline region. After leaf width was greater than 0.45 mm, we did not observe further non-stomatal divisions in the midline region, similar to the situation in *spch* leaves at a comparable width (Fig 1A, 58-74h, 0.48 mm). These results suggest that similar dynamics occur in the non-stomatal lineages of wild type and the *spch* mutant.

To determine how *SPCH* modulates division, we analysed stomatal and non-stomatal divisions in the lamina. Considerable variation was observed for both the area at which cells divide (25–400 *μ*m^2^) and cell cycle duration (8–50 h) (S13 Fig). The mean area at which cells execute division was greater for non-stomatal divisions (about 165 ± 28 *μ*m^2^ [1.96 × standard error]) than stomatal divisions (about 80 ± 6 *μ*m^2^) (S13 Fig). Similarly, cell cycle durations were longer for non-stomatal divisions (about 25 ± 3 h) compared with stomatal divisions (about 18 ± 1 h). These results suggest that in addition to conferring division competence, SPCH acts cell autonomously to promote division at smaller cell sizes and/or for shorter cell cycle durations.

### Wild type and *spch* can grow at similar rates despite different division patterns

Given the alteration in cell sizes and division patterns in wild type compared to *spch*, we wondered if these may reflect alterations in growth rates. When grown on agar plates, *spch* mutant leaves grow more slowly than wild-type leaves (S14A Fig). The slower growth of *spch* could reflect physiological limitations caused by the lack of stomata, or an effect of cell size on growth—larger cells in *spch* cause a slowing of growth. However, the tracking data and cell size analysis of *spch* and wild type described above were carried out on plants grown in a bio-imaging chamber in which nutrients were continually circulated around the leaves. Growth rates for wild type and *spch* leaves grown in these conditions were comparable for much of early development, and similar to those observed for wild type on plates (compare Fig 7D with Fig 1B, S14 Fig). These results suggest that the reduced growth rates of *spch* compared with wild type at early stages on plates likely reflect physiological impairment caused by a lack of stomata rather than differences in cell size. As a further test of this hypothesis, we grew *fama* (basic helix-loop-helix transcription factor bHLH097) mutants, as these lack stomata but still undergo many stomatal lineage divisions [27]. We found that *fama* mutants attained a similar size to *spch* mutants on plates, consistent with the lack of stomata being the cause of reduced growth in these conditions (S14 Fig).

Plots of cell area against growth rates of tracked leaves grown in the chamber showed that, for similar growth rates, cells were about three times smaller in wild type compared with *spch* (compare Fig 4A with Fig 4B). Thus, the effects of *SPCH* on division can be uncoupled from effects on growth rate, at least at early stages of development.

At later stages (after leaves were about 1 mm wide), *spch* growth in the bio-imaging chamber slowed down compared with wild type, and leaves attained a smaller final size. This later difference in growth rate might be explained by physiological impairment of *spch* because of the lack of stomata, and/or by feedback of cell size on growth rates. This change in later behaviour may reflect the major developmental and transcriptional transition that occurs after cell proliferation ceases [9].

### An integrated model of growth and division

The above results reveal that patterns of growth rate, cell division, and cell size and shape exhibit several features in *spch*: (1) a proximal corridor of cell division competence, with an approximately fixed distal limit relative to the petiole-lamina boundary; (2) the distal limit is greater for subepidermal (400 *μ*m) than epidermal tissue (300 *μ*m); (3) a further proximal restriction of division competence in the epidermis at early stages that extends with growth until the distal limit of the corridor (300 *μ*m) is reached; (4) larger and narrower cells in the proximal midline region of the epidermis; (5) a proximodistal gradient in cell size in the epidermal lamina; (6) a negative correlation between cell size and growth rate that is stronger in the epidermis than subepidermis; (7) variation in both the size at which cells divide and cell cycle duration along both the proximodistal and mediolateral axes; and (8) variation in growth rates parallel or perpendicular to the leaf midline. In wild-type plants, these patterns are further modulated by the expression of SPCH, which leads to division execution at smaller cell sizes and extension of competence, without affecting growth rates at early stages. Thus, growth and division rates exhibit different relations in adjacent cell layers, even in *spch*, in which epidermal-specific stomatal lineages are eliminated, and division patterns can differ between genotypes (wild type and *spch*) without an associated change in growth rates.

These observations argue against spatiotemporal regulators acting solely on the execution of division, which then influences growth, as this would be expected to give conserved relations between division and growth. For the same reason, they argue against a single-point-of-control model in which spatiotemporal regulators act solely on growth, which then secondarily influences division. Instead, they suggest dual control, with spatiotemporal regulators acting on both growth and division components. With dual control, growth and division may still interact through cross-dependencies, but spatiotemporal regulation does not operate exclusively on one or the other.

**Fig 8:**
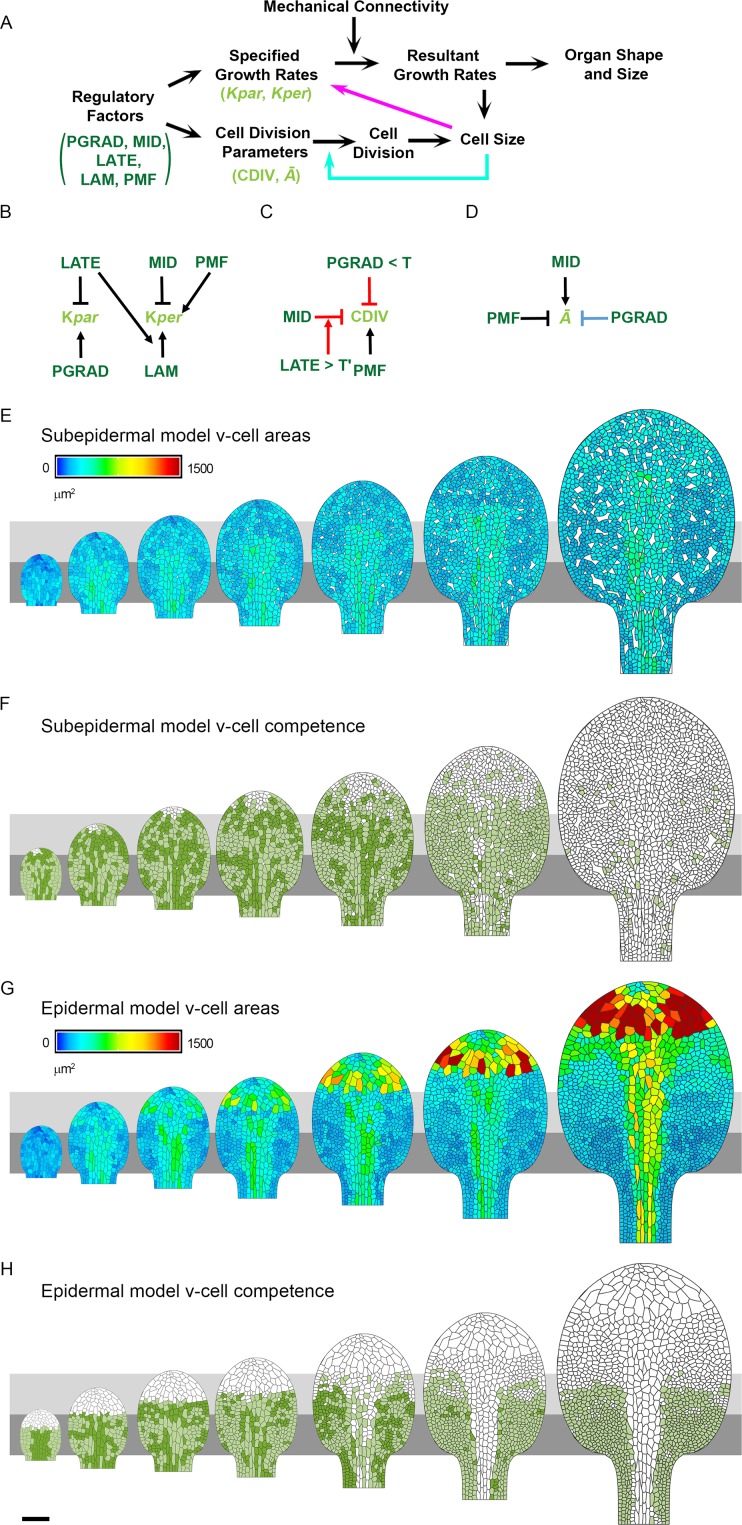
Model regulatory network and output. (**A**) Schematic showing how spatiotemporal regulatory factors (PGRAD, MID, LATE, LAM, PMF) act on components of specified growth (*K*_*par*_, *K*_*per*_) and cell division (CDIV and *Ā*) to influence resultant growth, cell division, cell size, and organ shape and size. The magenta arrow shows an optional feedback on growth rate from cell size. The cyan arrow shows feedback from cell size to division. (**B-D**) Model regulatory networks for early stages of leaf development (124–182 h). (**B**) Growth regulatory network (KRN). Epidermal-specific interactions are denoted with red lines, subepidermal-specific interactions with light blue, and interactions common to both models with black. (**C**) The regulatory network for CDIV. (**D**) The division threshold (*Ā*) regulatory network. (**E-H**) Model output leaves (left to right) at 115, 132, 140, 147, 156, 164, and 178 h for the (**E-F**) subepidermis model and (**G-H**) epidermis model. (**E**) Area and pattern of v-cells (heat map) with intercellular spaces (white). (**F**) v-cells that were competent to divide (green) and either executed division during the interval (light green) or divided in a later interval (dark green). Cells that did not divide (white). (**G**) Area and pattern of v-cells (heat map). (**H**) v-cells that were competent to divide (green) and either executed division during the interval (light green) or divided in a later interval (dark green). Cells that did not divide (white). Grey boxes are aligned to the petiole-lamina boundary and extend to 150 and 300 μm. Scale bar = 100 μm. See also S15 Fig and S16 Fig. Source data are available from https://figshare.com/s/b14c8e6cb1fc5135dd87. *Ā*, threshold cell area for division execution; CDIV, competence to divide; *K*_*par*_, specified growth rate parallel to polarity axis; *K*_*per*_, specified growth rate perpendicular to polarity axis; KRN, growth regulatory network; LAM, a factor distinguishing lamina from petiole; LATE, a timing factor; MID, a mediolateral factor; PGRAD, a graded proximodistal factor; PMF, proximal mobile factor; v-cell, virtual cell.

To determine how a hypothesis based on dual control may account for all the observations, we used computational modelling. We focussed on the epidermal and subepidermal layers of the *spch* mutant, as these lack the complications of stomatal lineages. For simplicity and clarity, spatiotemporal control was channelled through a limited set of components for growth and division (Fig 8A). There were two components for growth under spatiotemporal control: specified growth rates parallel and perpendicular to a proximodistal polarity field (*K*_*par*_ and *K*_*per*_, respectively) [16]. Together with mechanical constraints of tissue connectivity, these specified growth components lead to a pattern of resultant growth and organ shape change [28]. There were two components for cell division under spatiotemporal control: competence to divide (CDIV), and a threshold area for division execution that varies around a mean (*Ā*). Controlling division execution by a threshold cell size (*Ā*) introduces a cross-dependency between growth and division, as cells need to grow to attain the local threshold size before they can divide. The cross-dependency is indicated by the cyan arrow in Fig 8A, feeding information back from cell size (which depends on both growth and division) to division. An alternative to using *Ā* as a component of division-control might be to use a mean cell cycle duration threshold. However, this would bring in an expected correlation between high growth rates and large cell sizes (for a given cell cycle duration, a faster-growing cell will become larger before cycle completion), which is the opposite trend of what is observed.

Spatiotemporal regulators of growth and division components can be of two types: those that become deformed together with the tissue as it grows (fixed to the tissue) and those that maintain their pattern to some extent despite deformation of the tissue by growth (requiring mobile or diffusible factors) [28]. In the previously published growth model, regulatory factors were assumed, for simplicity, to deform with the tissue as it grows [16]. These factors comprised a graded proximodistal factor (PGRAD), a mediolateral factor (MID), a factor distinguishing lamina from petiole (LAM), and a timing factor (LATE) (S15A and S15B Fig). However, such factors cannot readily account for domains with limits that remain at a constant distance from the petiole-lamina boundary, such as the observed corridors for division competence. This is because the boundary of a domain that is fixed to the tissue will extend with the tissue as it grows. We therefore introduced a mobile factor, proximal mobile factor (PMF), that was not fixed to the tissue to account for these behaviours. This motivation is similar to that employed by others [11–13]. PMF was generated at the petiole-lamina boundary and with appropriate diffusion and decay coefficients such that PMF initially filled the primordium and then showed a graded distribution as the primordium grew larger, maintaining a high concentration in the proximal region and decreasing towards the leaf tip (S15C and S15D Fig). This profile was maintained despite further growth, allowing thresholds to be used to define domains with relatively invariant distal limits. Further details of the growth model are given in Materials and methods, and the resultant growth rates are shown in S16 Fig (compare with Fig 1B and 1D).

Cells were incorporated by superimposing polygons on the initial tissue or canvas (S15A Fig, right). The sizes and geometries of these virtual cells (v-cells) were based on cells observed at corresponding stages in confocal images of leaf primordia [16]. The vertices of the v-cells were anchored to the canvas and displaced with it during growth. Cells divided according to Errera’s rule: the shortest wall passing through the centre of the v-cell [29], with noise in positioning of this wall incorporated to capture variability. V-cells were competent to divide if they expressed factor CDIV, and executed division when reaching a mean cell target area, *Ā*. As the observed area at time of division was not invariant (Fig 2F), we assumed the threshold area for division varied according to a standard deviation of *σ* = 0.2*Ā* around the mean. CDIV and *Ā* are the two core components of division that are under the control of spatiotemporal regulators in the model (Fig 8A, 8C and 8D). Variation between epidermal and subepidermal patterns reflects different interactions controlling cell division (interactions colour coded red and blue, respectively, in Fig 8C and 8D).

We first modelled cell divisions in the subepidermis, as this layer shows a more uniform pattern of cell sizes (Fig 3B and Fig 6A). Formation of intercellular spaces was simulated by replacing a random selection of cell vertices with small empty equilateral triangles, which grew at a rate of 2.5% h^−1^, an average estimated from the tracking data. To account for the distribution of divisions and cell sizes, we assumed that v-cells were competent to divide (express CDIV) where PMF was above a threshold value. This value resulted in the competence zone extending to a distal limit of about 400 *μ*m. To account for the proximodistal pattern of cell areas in the lamina (Fig 3B and Fig 6A) and larger cells in the midline (Fig 3D and Fig 6A), we assumed that *Ā* was modulated by the levels of PMF, PGRAD, and MID (Fig 8D, black and blue). These interactions gave a pattern of average v-cell areas and division competence that broadly matched those observed (compare Fig 8E and 8F with Fig 6A and 6B, and Fig 3F and 3H with 3B and 3D, S3 Video).

For the epidermis, the zone of division competence was initially in the proximal region of the primordium and then extended with the tissue as it grew (Fig 1A). We therefore hypothesised that in addition to division being promoted by PMF, there was a further requirement for a proximal factor that extended with the tissue as it grew. We used PGRAD to achieve this additional level of control, assuming CDIV expression requires PGRAD to be above a threshold level (Fig 8C, red and black). V-cells with PGRAD below this threshold were not competent to divide, even in the presence of high PMF. Thus, at early stages, when PMF was high throughout the primordium, the PGRAD requirement restricted competence to the proximal region of the leaf (Fig 8H). At later stages, as the PGRAD domain above the threshold extended beyond 300 *μ*m, PMF became limiting, preventing CDIV from extending beyond about 300 *μ*m. To account for the earlier arrest of divisions in the midline region (Fig 1A), CDIV was inhibited by MID when LATE reached a threshold value (Fig 8C, red). As well as CDIV being regulated, the spatiotemporal pattern of *Ā* was modulated by factors MID and PMF (Fig 8D black).

With these assumptions, the resulting pattern of epidermal divisions and v-cell sizes broadly matched those observed experimentally for the epidermis (compare Fig 8G with Fig 1E, S4 Video). In particular, the model accounted for the observed increases in cells sizes with distance from the petiole-lamina boundary, which arise because of the proximal restrictions in competence (compare Fig 3E and 3G with Fig 3A and 3C). The model also accounted for the elongated cell shapes observed in the midline region, which arise through the arrest of division combined with low specified growth rate perpendicular to the polarity. Moreover, the negative correlations between growth rates and cell size, not used in developing the model, were similar to those observed experimentally (Fig 4B and 4D). These correlations arise because both growth and division are promoted in proximal regions.

We also measured the cell topology generated by the epidermal model. It has previously been shown that the frequency of six-sided neighbours observed experimentally for the *spch* leaf epidermis is very low compared with that for other plant and animal tissues and also with that generated by a previous implementation of Errera’s rule (S17 Fig) [30]. The topological distribution generated by the epidermal leaf model gave a six-sided frequency similar to that observed experimentally, falling two standard deviations away from the mean and thus close to a reasonable fit (S17 Fig). The increased similarity of the model output to the *spch* leaf epidermal topology, compared with a previous implementation of Errera’s rule [31], may reflect the incorporation of anisotropic growth in our model. If polarity is removed from our model to render specified growth as isotropic (while preserving local areal growth rates), the frequency of six-sided neighbours increases, becoming more like the empirical data for the shoot apical meristem (S17 Fig). A further likely contribution to the lowering of six-sided neighbour frequency generated by our model is the use of random noise to displace the positioning of new walls, rather than positioning them always to pass precisely through the cell centre. Thus, our analysis shows how incorporating more realistic growth patterns can be valuable in evaluating division rules.

Taken together, the simulations show that the pattern of growth and division can be broadly accounted for by factors modulating specified growth rates (*K*_*par*_ and *K*_*per*_) and cell division components (CDIV and *Ā*). Variation between epidermal and subepidermal patterns generated by the models reflects different interactions controlling cell division (Fig 8C and 8D).

### Modulation of model parameters leads to variation in leaf size, cell number, and cell size

Many mutants have been described that influence cell division and/or leaf size [32,33]. To gain a better understanding of such mutants, we explored how changes in key parameters in our model may alter leaf size, cell size, and cell number. As leaf size is normally measured at maturity, we first extended our analysis to later stages of development. Tracking *spch* to later stages of development showed that overall growth rates declined, on average, while remaining relatively high towards the proximal region of the lamina (S4B Fig), consistent with a previous study [18]. Cell divisions were not observed after the leaf reached a width of about 0.9 mm (S4A Fig, 96h). To capture arrest of division, we assumed that CDIV was switched off throughout the leaf after LATE reached a threshold value.

**Fig 9:**
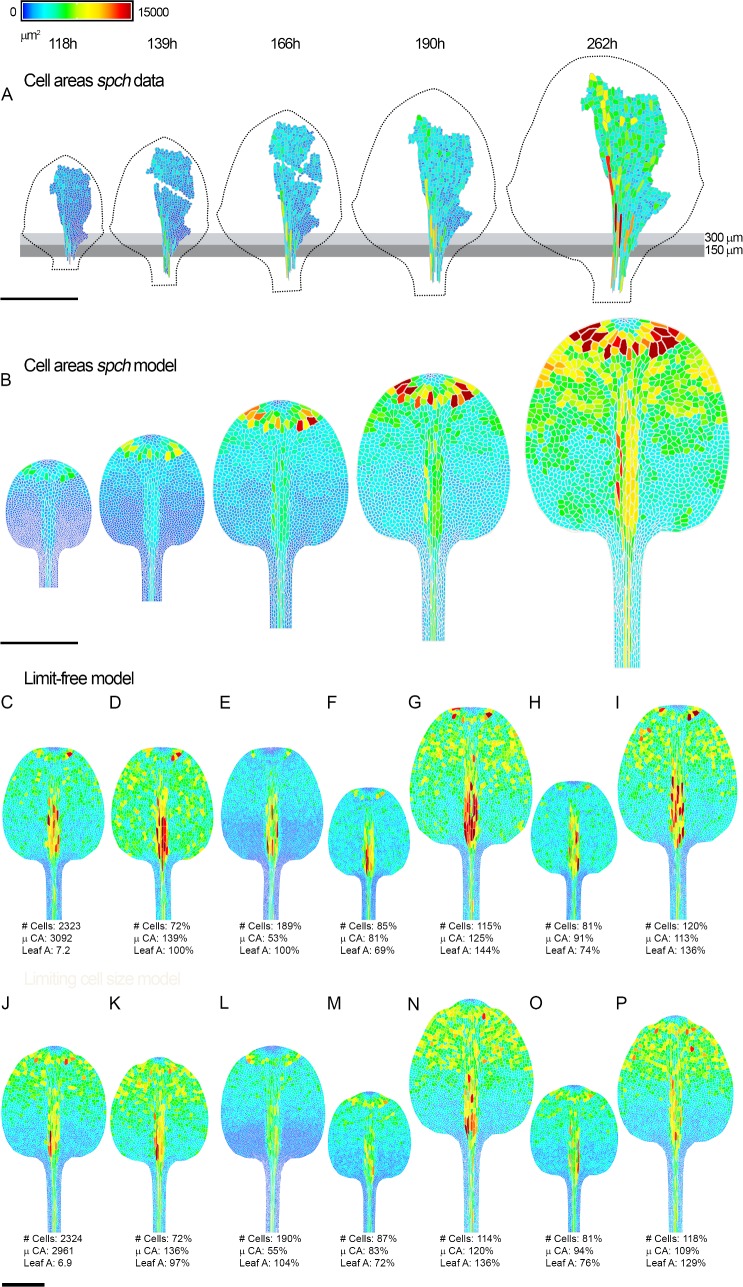
Cell areas, numbers, and leaf sizes at later developmental stages. (**A**) Cell areas for the *spch* leaf at later stages (earlier stages shown in S4 Fig). Leaf widths (left to right) are 1.1, 1.4, 1.8, 2.1, and 2.8 mm. Leaf outline is indicated by a dotted black line. Grey boxes are aligned to the petiole-lamina boundary and extend to 150 or 300 μm. (**B**) Model output at equivalent stages to experimental data (model times 201, 219, 243, 264, and 412 h). (**C-I**) Limit-free *spch* epidermal model outputs at maturity. (**C**) Wild type. (**D**) Cell division threshold increased by 85 μm^2^. (**E**) Cell division threshold decreased by 85 μm^2^. (**F**) Specified growth rate reduced by 5%. (**G**) Specified growth rate increased by 5%. (**H**) LATE comes on 6 h earlier. (**I**) LATE comes on 6 h later. (**J-P**) Limiting cell size *spch* epidermal model outputs at maturity. (**J**) Wild type. (**K**) Cell division threshold increased by 85 μm^2^. (**L**) Cell division threshold decreased by 85 μm^2^. (**M**) Specified growth rate reduced by 5%. (**N**) Specified growth increased by 5%. (**O**) LATE comes on 6 h earlier. (**P**) LATE comes on 6 h later. Number of cells (# Cells), average cell area (μ CA) in μm^2^, and leaf area (Leaf A) in mm^2^ are shown for each model. Percentage values for mutants show relative difference to limit-free (**C**) and limiting cell size (**J**) wild-type models. Scale bars = 1 mm. Source data are available from https://figshare.com/s/b14c8e6cb1fc5135dd87. # Cells, number of cells; μ CA, average cell area; LATE, a timing factor; Leaf A, leaf area; *spch*, *speechless*.

In the previously published growth model [16], the decline of growth rates with developmental time was captured through an inhibitory effect of LATE on growth. To extend the model to later stages and bring about eventual arrest of growth, we assumed that LATE increased exponentially after 189 h and inhibited both *K*_*per*_ and *K*_*par*_ thereafter. Parameters for growth inhibition were adjusted to give a final leaf width of about 3 mm, which was the final size attained for leaf 1 in *spch* mutants in the bio-imaging chamber. The v-cell sizes generated by the model broadly matched the patterns observed (Fig 9A and 9B, S5 Video). As epidermal divisions have ceased by the time the *spch* leaf is about 1 mm wide, all the growth depicted in Fig 9A and 9B occurs in the absence of division (i.e., cell expansion). However, a notable discrepancy between the model output and the experimental data was the generation of distal v-cells that exceeded the values observed (about 20,000 μm^2^ compared with about 10,000 μm^2^). A similar result was obtained if the model was tuned to match not only the final leaf width but also the reduced growth rate of *spch* in the growth chamber at later stages (S14B and S14C Fig). A better fit was obtained by inhibiting specified growth rates in distal regions at later stages. This inhibition was implemented by introducing inhibitory factors with levels that increased distally. The result was that distal v-cells remained at or below about 10,000 μm^2^ (Fig 9C and S6 Video). We refer to this as the limit-free model. Another way of limiting the size of distal v-cells was to introduce feedback from cell size to growth, so that the specified growth rate decreased as v-cells approached upper size limits (Fig 9J and S7 Video). This feedback corresponds to introducing a further interaction in the regulatory pathway (Fig 8A, magenta). We refer to this as the limiting cell size model.

We varied parameters in both the limit-free model (Fig 9C) and the limiting cell size model (Fig 9J) to see how the parameters influence cell number, cell size, and final leaf size. Increasing *Ā* by a constant amount did not change leaf size with the limit-free model but resulted in fewer, larger v-cells (Fig 9D). Reducing *Ā* resulted in a leaf with more v-cells that were, on average, smaller but did not change leaf size (Fig 9E). With the limiting cell size model, increasing or decreasing *Ā* had similar effects as with the limit-free model but also slightly reduced or increased leaf size (Fig 9K and 9L). Thus, it is possible to affect cell number and size without a major effect on organ size or growth.

To investigate how changing growth parameters influences cell numbers and areas, we reduced the specified growth rates (values for *K*_*par*_ and *K*_*per*_) by 5%. For the limit-free model this resulted in a smaller leaf with both smaller and fewer v-cells (Fig 9F). There were fewer cells because they grew more slowly and thus took longer to reach *Ā*, and cells were smaller because they grew at a slower rate after they had ceased dividing. Conversely, increasing specified growth rate by 5% led to larger leaves, with more v-cells that were, on average, larger (Fig 9G). The model with limiting cell size gave similar results (Fig 9M and 9N). Thus, modulating growth rates has consequences on organ size, cell size, and cell number. This may account for why many mutants with smaller organs have both fewer cells and smaller cells [34].

To examine the effect of changes in developmental timing, we altered the onset of LATE. Moving the onset earlier for the limit-free model led to smaller leaves because of the earlier decline in growth rate (Fig 9H). There were fewer v-cells because of the earlier arrest of division, and there was also a slight reduction in v-cell size. Delaying the onset of LATE had the opposite effect of increasing leaf size, cell number, and cell size (Fig 9I). The limiting cell size model gave similar results (Fig 9O and 9P). Thus, changes in developmental timing affected organ size and cell number, with a lesser effect on cell size. This is because changing LATE shifts both the onset of the growth rate decline and the time of division arrest (inactivation of CDIV).

**Fig 10:**
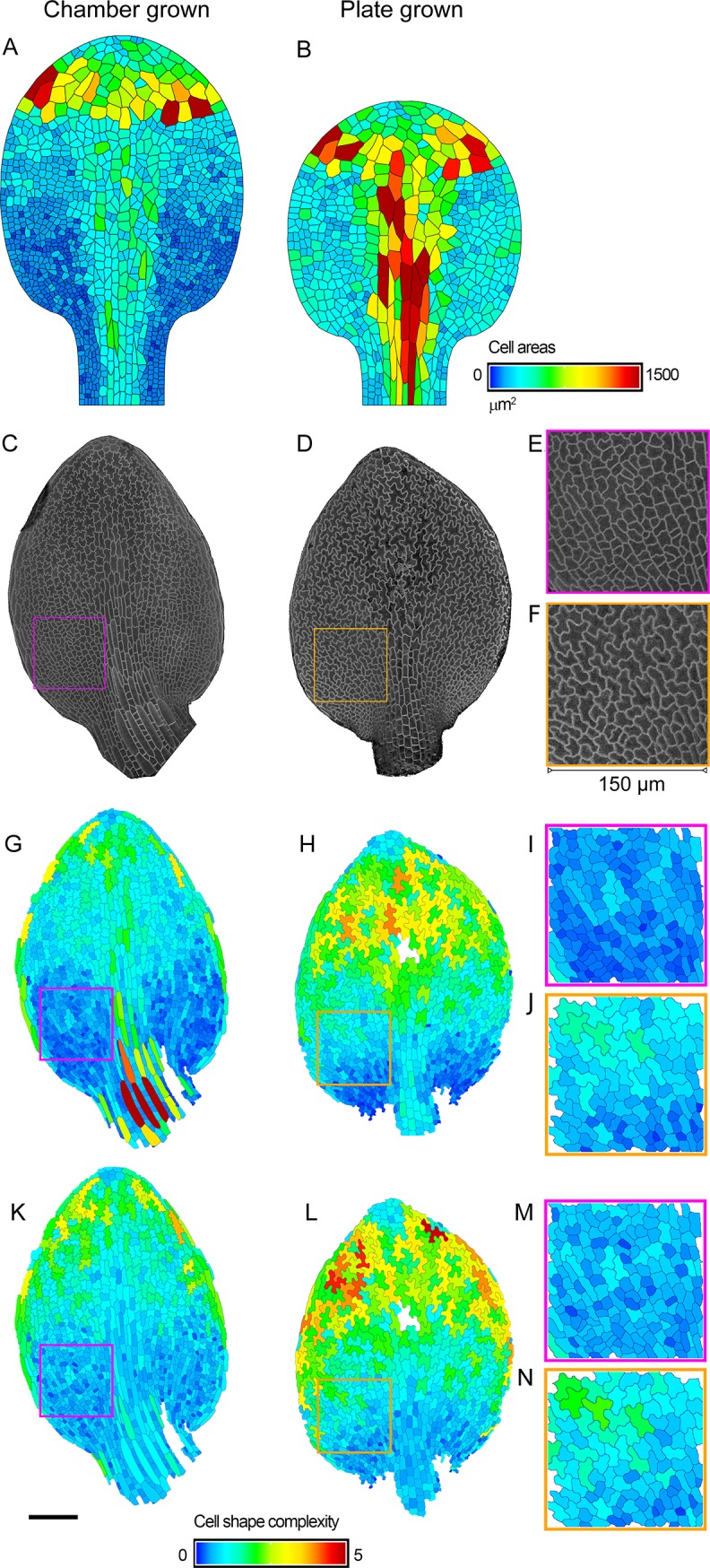
Comparison of *spch* epidermis grown in a bio-imaging chamber and on plates. (**A**, **B**) Model outputs when leaf has grown to a width of about 0.5 mm (see horizontal line in S14A Fig). (**A**) Epidermal model used to generate Fig 8G, corresponding to growth in a chamber. (**B**) Output from epidermal model tuned to match *spch* growth on plates (by slowing growth by 40% and physiological time by 45%). **(C)** Leaf grown in a bio-imaging chamber when width has attained 0.46 mm (8DAS). **(D)** Leaf grown on plates when width has attained 0.48 mm (13 DAS). **(E)** Enlargement of region indicated by magenta square in C. (**F**) Enlargement of region indicated by orange square in D. **(G,H)** Segmented cells from leaves shown in C,D. Cell area colour coded as heat map for A,B. **(I)** Enlargement of segmented region indicated by magenta square in G; average cell area 123.3 ± 6.4 μm^2^ (*n* = 184). All cells with their centroid falling within the square were taken into account. **(J)** Enlargement of segmented region indicated by orange square in H; average cell area 199.8 ± 17.3 μm^2^ (*n* = 111). **(K,L)** Cell complexity from leaves shown in C,D, quantified through the CD of the LOCO-EFA components of each individual cell’s shape, normalised for cell area (see Materials and methods). Heat map range corresponds to zero for perfect circular or elliptical shapes, ranging to 5 as more LOCO-EFA harmonics are needed to describe the shape (dimensionless measure). (**M**) Enlargement of region indicated by magenta square in K. (**N**) Enlargement of region indicated by orange square in L. Scale bar = 100 μm. Source data are available from https://figshare.com/s/b14c8e6cb1fc5135dd87. CD, cumulative difference; DAS, days after stratification; LOCO-EFA, Lobe-Contribution Elliptic Fourier Analysis; *spch*, *speechless*.

A further application of the model is to explore the effects of the environment on leaf growth and division. To illustrate this possibility, we analysed data for the *spch* mutant grown on plates, which exhibits a greatly reduced growth rate compared with growth in the chamber (S14A and S14B Fig). A prediction of the model is that cell divisions should cease when the leaf is at a smaller size (i.e., the leaf will have grown less by the time the threshold value of LATE for division arrest is reached). In addition, as *spch* plants grown on plates have impaired general physiology, the rate of developmental progression (physiological time) may also be slowed down. We simulated these effects by modifying the model parameters such that the overall growth rate was reduced by 40% and physiological time reduced by 45%. This gave a growth curve matching that observed for *spch* grown on plates (blue line, S14A Fig). As expected, this model takes longer to attain a given leaf width (e.g., 0.5 mm) than the original model. The resulting cell areas are larger at the 0.5-mm leaf-width stage, particularly in proximal regions, because divisions arrest when the leaf is at a smaller size, so all subsequent cell growth occurs in the absence of division (Fig 10A and 10B and S18 Fig).

To test this prediction of enlarged cell size, we compared leaves when they had attained a width of about 0.5 mm (Fig 10C and 10D), which is just before divisions cease for *spch* grown in the chamber (Fig 1). Cells in the proximal lamina of the chamber-grown leaves were relatively small (mean = 123.3 ± 6.4 μm^2^ for region shown in Fig 10I), typical of dividing cells (Fig 10C and 10G); whereas those of the plate-grown leaves were larger (mean = 199.8 ± 17.3 μm^2^ for region shown in Fig 10J), indicating division arrest (Fig 10D and 10H and S18 Fig). Proximal lamina cells in plate-grown leaves also showed greater shape complexity, typical of pavement cells that have ceased division (Fig 10K–10N and S18 Fig). These results suggest that cell divisions in much of the lamina cease when the leaf is smaller for plate-grown compared to chamber-grown leaves, as predicted by the model. The sizes of midline cells for plate-grown leaves predicted by the model are larger than those observed (compare Fig 10B with Fig 10H), indicating that withdrawal of competence from this region, as implemented in the model, may be activated too early. Conversely, the most proximal lamina cells in the plate-grown leaves (dark blue cells, Fig 10H) are smaller than predicted (Fig 10B), suggesting that the uniform arrest of division when LATE reaches a threshold value is an oversimplification.

## Discussion

Growth rates, cell division, and cell shapes and sizes in the growing first leaf of *Arabidopsis* exhibit complex spatiotemporal patterns. The main features observed in *spch* are (1) a proximal corridor of division competence with an approximately fixed distal limit; (2) the distal limit is greater for subepidermal compared to epidermal tissue; (3) a further proximal restriction of division competence in the epidermis at early stages that extends with growth until the distal limit is reached; (4) a proximodistal gradient in cell size in the epidermal lamina; (5) larger and narrower cells in the proximal midline region of the epidermis; (6) a negative correlation between cell size and growth rate that is stronger in the epidermis than in the subepidermis; (7) variation in both the size at which cells divide and cell cycle duration along both the proximodistal and mediolateral axes; (8) variation in growth rates parallel or perpendicular to the leaf midline. In wild-type plants these patterns are further modulated by expression of SPCH, which leads to division execution at smaller cell sizes and extension of competence, without affecting growth rates at early stages.

The observed varying relations between growth rates and division between tissue layers and genotypes argue against single-point-of-control models, in which spatiotemporal regulators act solely through either division or growth. Instead, they suggest dual control, in which spatiotemporal regulators act on both growth and division, with cross talk between them. We show that a model based on dual control can broadly account for the data. In this model, spatiotemporal control is channelled through two growth components (specified growth rates parallel and perpendicular to polarity) and two division components (competence and mean threshold size for division) (Fig 8A). The growth components reflect turgor and cell wall extensibility in different orientations, and the division components reflect regulatory mechanisms for partitioning cells. Orientation information is provided by a tissue-wide polarity field, for which direct evidence has recently been obtained in both wild-type and *spch* mutants [22,35]. The polarity field may be established through a biochemical mechanism as proposed here, likely involving tissue-level coordinated cell polarity [36]. Alternatively, information could be relayed through mechanical stresses [20,22,37]. The resulting patterns of growth and division determine the distribution of cell sizes and shapes and organ shape. The implications, limitations, and questions raised by this model are discussed below.

### Growth, division, and cell size

Execution of leaf cell division does not occur at an unvarying cell size, even within a given region and developmental stage. Similar variability has been observed for cell divisions in apical meristems [21,38]. Variability may reflect experimental errors in estimation of cell size, stochasticity in the process of division, and/or mechanisms other than geometric size sensing that influence division execution (e.g., factors such as vacuole size, which is not monitored in our analysis). We model such variability by explicitly adding variation around a mean threshold size needed for division, *Ā*. Controlling division execution by a threshold cell size (*Ā*) introduces a cross-dependency between growth and division, as cells need to grow to attain the local threshold size before they can divide. An alternative to using *Ā* would be to use a mean cell cycle duration threshold. However, this would bring in an expected correlation between high growth rates and large cell sizes (for a given cell cycle duration, faster growing cells will become larger before cycle completion), which is the opposite of the correlation observed.

### Role of tissue layers in growth

In contrast to the epidermal layer, intercellular spaces are observed in the subepidermis of wild-type and *spch* from early stages. The spaces may originate, in part, from a reduction in adhesion between subepidermal cells, allowing cell walls to become detached from each other. In addition to reduced adhesion, a further requirement for intercellular spaces is that cells are not too tightly packed against each other. Packing may be reduced if subepidermal cells have lower specified growth rates than the epidermis. Subepidermal cells could move away or be pulled apart from each other, as epidermal growth creates more space than they can fill through their own expansive growth. According to this view, the epidermis rather than the subepidermis provides the expansive force driving planar growth, in contrast to what has been described for other tissues, such as the stem [39]. A primary role for the epidermis in driving planar growth is also consistent with the observed developmental effects of epidermal gene activity [40]. However, it is possible that the subepidermis provides a restraint on growth, which could account for the effect of subepidermal tissue on leaf shape in some chimeras [41].

### Spatiotemporal control

Spatiotemporal control of growth and division in the model of *spch* is established through combinatorial interactions between five factors: PGRAD, MID, LAM, LATE, and a mobile factor that allows proximal corridors with fixed distal limits to be established (PMF). PMF is similar to the previously proposed mobile growth factor [11], except that the effect of PMF on division does not have a consequential effect on growth. To account for the difference in distal limits of the division corridor between cell layers, PMF action extends more distally in the subepidermis compared with the epidermis, either because the competence threshold requirement for PMF is lower in the subepidermal layer, or because PMF levels are higher. A candidate factor for coordinating proliferation between layers is the transcriptional coactivator *ANGUSTIFOLIA3* [42,43]. Candidates for LAM are *LEAFY PETIOLE* [44] and members of the *YABBY* gene family [45], which are expressed in the lamina and promote lateral outgrowth.

A fixed corridor for division has also been described for other systems such as the root, where a division zone is maintiained at a distance of about 300–500 μm from the quiescent centre in *Arabidopsis* [46]. In contrast to the leaf, regions of highest growth rate in the root are outside the cell division zone, providing further support for a dual control mechanism. The spatial extent of the division zone in roots is maintained through auxin-cytokinin interactions [47]. Auxin-cytokinin interactions also influence leaf growth and division: temporal arrest of leaf growth depends on auxin-induced cytokinin breakdown [48]; increased cytokinin degradation in leaf primordia can accelerate termination of cell proliferation [49]; and accumulation of specific cytokinins may promote indeterminate leaf growth [50]. However, it is currently unclear whether auxin, cytokinin, and/or other molecular players underlie PMF.

A limitation of our model is that it does not consider modulation of growth or division near the leaf margin, creating serrations [51,52]. Serrations have previously been modelled by displacement of the leaf outline without modelling the tissue growth explicitly [52,53]. In terms of the modelling framework described here, they may reflect alterations in polarity and/or growth rates of tissue, and accounting for these behaviours would require the introduction of additional factors into the model, as illustrated by generation of winglike outgrowths in barley lemma mutants [54].

To account for the further proximal restriction of competence in the epidermis at early stages, PGRAD limits divisions in the epidermis until the distal limit set by PMF is reached. PMF also interacts with MID in the epidermis, accounting for larger cells in the midline region. The elongated shape of proximal midline cells is a result of early arrest of division combined with low specified growth rates perpendicular to the proximodistal polarity.

Divisions in the wild-type epidermis are also influenced by *SPCH*. We show that *SPCH* acts autonomously in the epidermis to confer competence, and has little impact in the proximal midline region, where its activity has previously been shown to be low [55]. The autonomous effect of *SPCH* on division competence contrasts with its nonautonomous effects at later stages of development, with regard to layer thickness and photosynthetic capacity [56]. This difference in autonomy may reflect primary and secondary consequences of *SPCH* activity. *SPCH* also promotes asymmetric divisions and divisions at smaller cell sizes or shorter cell cycle durations. The complex pattern of divisions in wild type epidermis observed here and elsewhere [9] would thus reflect the combined effect of PMF, PGRAD, MID, and *SPCH*, although the molecular basis of these interactions remains to be established.

In agreement with [24], we observed that mean cell cycle duration is relatively constant for wild type (about 20 h). However, cell cycle duration varies from 8 h to 50 h around the mean. Some of this variation depends on whether SPCH is active: epidermal cells that do not show high SPCH activity divide at a larger cell size and longer cell cycle duration. Moreover, the size at which cells with active SPCH divide is not fixed but becomes progressively smaller with successive divisions [23], indicating that cell cycle duration likely becomes shorter as well. Thus, the spatiotemporal variation in cell cycle duration may be the consequence of variation in growth rates (for a given threshold division size, cell cycle duration depends on growth rate) and/or direct control of cell cycle length.

### Leaf size, cell size, and cell number

Most small-leaf mutants have both fewer and smaller cells [34]. Such outcomes can be generated with the model by reducing specified growth rates. The leaves end up smaller because of the lower growth rate, cells are smaller because they grow less after divisions have arrested, and there are fewer cells because they grow more slowly and thus take longer to reach *Ā*. Thus, the observation that organ size, cell size, and cell number are commonly reduced together in mutants is a natural outcome of the model.

Change in developmental timing through factor LATE also leads to changes in leaf size, although this is mainly reflected in changes in cell number rather than cell size. This is because changing LATE shifts both the onset of growth rate decline and the time of division arrest (loss of division competence). Such variation in developmental timing could underlie mutants that change organ size with little or no effect on cell size, such as *kluh* and *big brother* [57,58].

Loss of expression of D-type cyclins leads to premature termination of cell division and fewer cells autonomously in each layer, without a major change in leaf size [59,60]. Such features can be captured by changing model parameters that are specific to cell division, such as the value of *Ā*, in one or more layers (Fig 9D, 9E, 9K and 9L). This situation corresponds to compensation [61–63], as change in cell number is counterbalanced by a change in cell area (organ size is preserved). However, no dedicated mechanism for counterbalancing is needed, as division is under separate spatiotemporal control from growth in our model.

Although execution of division does not have an immediate effect on growth rates in our model, we explore the possibility of feedback from division on growth at later developmental stages. If growth slows down when cells approach an upper size limit, then cell division could postpone the slowing down of growth by reducing cell size. Such a mechanism would lead to cell division extending the duration of growth, thus increasing leaf size. Mature leaves display an array of final cell sizes that correlate with levels of endoreduplication [64,65], suggesting that as cells approach a size limit, endocycles are induced that allow them to surpass the limit. If endoreduplication is impaired, these cell size limits may not be so easily overcome, leading to smaller leaves with smaller cells [66,67]. However, the extent to which endoreduplication is limited in wild type and thus may constrain final cell size and growth is unclear. Through modelling, we show that it is possible to account for the data with or without feedback from cell size on growth.

If endocycles are promoted as cells enlarge, then promoting division (e.g., by reducing *Ā*) should lead to lower levels of endoreduplication (as cells will be smaller). This prediction is in accord with the effect of overexpressing D-type cyclins, which leads to smaller cells with lower levels of endoreduplication [68]. Conversely, inhibiting cell division (e.g., by increasing *Ā*) should give larger cells and higher levels of endoreduplication, as observed with *cycd3* mutants [7]. However, if both division and the ability to endoreduplicate are impaired, cell size may eventually feedback to inhibit growth rate, giving smaller leaves and perhaps accounting for the phenotype of *ant* mutants, which have smaller organs with larger cells that do not endoreduplicate more than wild type [7,69].

A further application of the model is to explore the effects of different environments on leaf growth and division. As an illustration, we compared leaves of *spch* mutants grown in a bio-imaging chamber (in which nutrients were continually circulated around the leaves) with those grown on agar plates (in which growth rate is greatly reduced). Cell divisions arrested when leaves were at a smaller size in the slow-growing conditions, as predicted by the model in which division arrest depends on a timing mechanism (LATE). However, the growth and cells sizes observed suggests that the timing mechanisms are not based on external time but passage of physiological time, which may also be affected by altered growth conditions.

The model presented here identifies core components of growth and division that may be regulated and interact to generate the spatiotemporal patterns observed. Further integrative studies on growth and division at the subcellular, cellular, and tissue level in different genotypes and environments should help provide a deeper understanding of the mechanisms by which regulatory factors are established and control these core components.

## Materials and methods

### Plant material and growth conditions

For tracking growth of the *speechless* mutant, we used the previously published *Arabidopsis* line, *spch-1*, containing a fluorescently labelled plasma membrane marker [70]. To more precisely determine division execution times, we crossed the *spch* mutant to an *Arabidopsis* line containing fluorescently labelled nuclei, *HTA11-GFP* [71], and *PIN3*:*PIN3-GFP* [72], which labels plasma membranes in the epidermal layer only. For tracking growth in the wild-type background and to distinguish cells in the stomatal lineage, we used the previously published *Arabidopsis* line containing *pSPCH*:*SPCH-GFP* and *PIN3*:*PIN3-GFP* [23]. For measuring leaf widths in the *fama* mutant we used the previously published line *fama-1* (Ohashi-Ito and Bergmann, 2006).

Seeds were surface sterilised with 70% ethanol containing 0.05% Sodium Dodecyl Sulfate (SDS) for 10 min and then rinsed with 100% ethanol. Sterilised seeds were sown on petri dishes containing 25 mL of MS growth media {1× Murashige and Skoog salt mixture, 1% (w/v) sucrose, 100 mg/mL inositol, 1 mg/mL thiamine, 0.5 mg/mL pyridoxin, 0.5 mg/mL nicotinic acid, 0.5 mg/mL MES, 0.8% (w/v) agar, pH 5.7} and kept at 4 ^o^C in the dark for 72 h (stratification). Plates were then transferred to a controlled environment room (CER) at 20 ^o^C in long-day conditions (16-h light/8-h dark cycles) for 5–8 d.

### Time-lapse imaging

At 5–8 d after stratification, seedlings were transferred under sterile conditions into an autoclaved optical live-imaging chamber [16,73] and continuously supplied with 1/4 strength MS liquid growth medium, including sucrose to support growth. Time-lapse imaging was carried out at regular intervals using a Leica SP5 Confocal microscope, a Zeiss LSM 5 EXCITER confocal microscope, or a Zeiss LSM 780 confocal microscope. For experiments imaged with a high temporal resolution (intervals of 1–2 h), the chamber remained mounted on the microscope stage for the duration of the experiment, with room temperature and photoperiod set to be similar to that of the CER in which seedlings were germinated. For experiments with a longer interval between imaging (12–24 h), the chamber was returned to the CER between confocal imaging. Experiments were carried out on leaf 1 within the range of 0.15–2.75 mm width. Seedlings were positioned in the chamber such that the abaxial epidermis of the leaf was oriented approximately parallel and adjacent to the coverslip, although it curved away to some extent at the leaf margins. This curvature affected the leaf outline produced when projected images were made from confocal image stacks. Leaf outlines (indicated by dotted lines in Fig 1, Fig 2, Fig 6, Fig 7, Fig 9, S2 Fig, S3 Fig, S4 Fig, S6 Fig, and S9 Fig) reflect projections onto the imaging plane rather than being corrected for curvature and thus convey a shape that appears narrower than the actual leaf outline. Some regions could not be tracked because of occlusion by overlapping leaves (at early developmental stages) or because movement in the z-dimension caused parts of the leaf to go out of focus. Thus, some cell lineages could not be traced all the way back to the initial time point. Images are available from https://figshare.com/s/b14c8e6cb1fc5135dd87.

### Image processing

To facilitate cell tracking, confocal image stacks were converted into 2D projections using either Volviewer [74] (http://cmpdartsvr3.cmp.uea.ac.uk/wiki/BanghamLab/index.php/VolViewer) or Fiji [75]. For early stages, when the leaf could be captured in a single scan, VolViewer was used to create a projection of the leaf surface. At later stages, when leaves were larger, multiple overlapping tiled scans were required to capture the entire leaf. In such cases, Fiji was used to create multiple 2D projections, which were merged together using Photoshop to create a single composite image. Leaf width was measured in 3D, when possible, using VolViewer. For later stages, leaf width was measured in 2D from merged projections using Fiji. Projections of the subepidermal layer were created in VolViewer using the ‘Depth-Peal Shader’ lighting editor. Several projections were created for each z-stack (using different parameters to reveal as many cells as possible in approximately the middle of the cell layer) and merged together using Photoshop to create a composite image.

### Tracking growth and divisions

Projected confocal images were used to calculate growth rates and cell areas and monitor cell division dynamics in 2D by placing points around the vertices of individual cells using *PointTracker*, as described in [16]. A toolset (Track ‘n’ R) was created for ImageJ (https://imagej.nih.gov/ij/) to facilitate access to ImageJ macros and offer improved visualisation of *PointTracker* data using R [76]. Track ‘n’ R was used to create leaf outlines, visualise the zone of cell division, and analyse cell lineages and to display cell cycle duration, cell area at division execution, and growth rates (source code and detailed instructions for Track’n’R and PointTracker have been deposited at https://github.com/fpantin/Track-n-R and https://figshare.com/s/b14c8e6cb1fc5135dd87 respectively).

Graphical outputs from Track ‘n’ R were reoriented so that the leaf tip pointed upwards. Cellular growth rates over a time interval t1–t2 were calculated according to ln(A_t2_−A_t1_)/(t2−t1), where A_t1_ is cell area at t1 and A_t2_ is cell area at t2. If a cell divided in this interval, A_t2_ was the area of the clone it gave rise to at time t2.

For each tracking experiment, the first row of nondividing cells was identified in the first time point and coloured orange by hand using Photoshop. These cells were identified in each subsequent image and also coloured orange (Fig 1A and S3 Fig and S4 Fig). The approximate location of the petiole-lamina boundary was identified based on the shape of the leaf outline in the last image available for each dataset. A cell was identified in the midline of this image, in line with the base of the leaf lamina. This cell was then traced back to through each image to its earliest ancestor in the first time point, thus identifying the location of the petiole-lamina boundary even when the leaf shape was less developed. Cells were identified as part of the midline region based on appearance (shape and location) in the last image of each tracking experiment. The lineage of these cells were traced back to the beginning of the experiment (S2 Fig). Cells that did not form part of the midline region were classed as lamina cells.

### Analysis of cell size using 3D segmentation

For 3D segmentation and volume measurements, confocal image stacks were processed using Python scripts, as described [17], with additional scripts added to measure the external surfaces of epidermal cells in 3D and the corresponding 2D projections (source code and detailed instructions have been deposited at https://figshare.com/s/b14c8e6cb1fc5135dd87). Fiji macros [75] using the 3D Viewer and Point Picker plugins were used to visualise images and select cells during manual quality control.

For the epidermis, plotting projected segmentation-based area against vertex-based area gave a good linear fit (*R*^2^ = 0.87) with a gradient of about 1, showing that vertex-based cell area is a good proxy for projected cell area (S5A Fig). Areas extracted from the cell surface plotted against vertex-based cell area also gave a good linear fit (*R*^2^ = 0.77) with a gradient of about 1.2 (S5B Fig). The higher value for the gradient likely reflects curvature of the cell surface and the leaf, both of which increase area compared to projected values. Nevertheless, segmented surface area remains linearly related to vertex-based area. Plotting cell volume against segmentation-based cell surface area gave a linear fit, with *R*^2^ = 0.91 and a gradient suggesting an approximately constant cell thickness of about 9 μm (S5C Fig). Variation in cell thickness is displayed by plotting cell volume divided by surface area as a heat map. Although a slight increase in cell thickness was observed in the proximal midline (about 15 μm), cell thickness showed relatively little spatial variation for much of the lamina (S5D Fig), compared with the striking spatiotemporal variation in cell area (S5E Fig) and cell volume (S5F Fig). Thus, the major contribution to cell size variation derives from cell area rather than cell thickness. These results are also consistent with fixed leaf sections shown in [10], which have epidermal cells in the range of 8–15 μm thick (measured according to the scale in the published images).

For the subepidermal cell layer, fewer cells could be segmented in 3D because the bases of the cells were too deep within the tissue to be captured clearly by confocal imaging. However, around 13 cells could be segmented and plotting projected segmentation-based area against vertex-based cell area gave a good linear fit (*R*^2^ = 0.98) with a gradient of about 1 (S11A Fig). Plotting the volume of these cells against projected segmentation-based cell areas showed that they had similar thickness to epidermal cells of the same area, except for cells in the proximal midline region, where subepidermal cells have a greater volume because of increased thickness (S11B and S11C Fig). These results are also consistent with fixed leaf sections shown in [10], which have subepidermal cells in the range of 9–14 μm thick.

### Analysis of cell shape complexity

To quantify cell shape complexity, we employed Lobe-Contribution Elliptic Fourier Analysis (LOCO-EFA), a method to decompose the outline of each cell into a list of biologically meaningful descriptors [77]. The LOCO-EFA decomposition was used to estimate a measure of cell shape complexity, coined the ‘cumulative difference’ (CD), which is the integral over all LOCO-EFA modes larger than 2 of the mismatch (Exclusive OR or XOR) between the original and reconstituted shape, yielding a scalar value representing the degree of shape complexity of each cell [77]. We used this measure normalised to cell area; hence, a small or large cell with the same shape will yield the same cell complexity measure (CD). It ranged from zero (low complexity, which describes perfectly circular or elongated cells) to higher values, as more LOCO-EFA harmonics are required to accurately describe the shape. The computer code was written in C and is available on a remote repository (Git repository), which is publicly available on Bitbucket (https://bitbucket.org/mareelab/LOCO_EFA).

### Analysis of *spch* expression

The expression pattern of *pSPCH*:*SPCH-GFP* was analysed from time-lapse images to distinguish cells in the stomatal lineages from non-stomatal lineages. For each cell division, the duration of *SPCH* expression was determined from the time when *SPCH* first became visible in the nucleus of the mother cell to when it could no longer be seen in each daughter cell. S1 Video shows an example of cell division in the stomatal lineage. S2 Video shows an example cell division in a non-stomatal lineage.

### Analysis of subepidermal cells in wild type

To facilitate imaging of the subepidermal cell layer in wild-type leaves, seedlings grown on plates were stained by the modified pseudo-Schiff propidium iodide (mPS-PI) method, as previously described [78]. After approximately 1 wk (for the mounting solution to set), leaf primordia were imaged using a Leica SP5 Confocal microscope. Projections of the subepidermal layer were created in VolViewer using the ‘Depth-Peal Shader’ lighting editor, as described above for *spch* in “Image processing”.

### Modelling leaf growth

All models and GFT-box software used for modelling can be downloaded from http://cmpdartsvr3.cmp.uea.ac.uk/wiki/BanghamLab/index.php/Software.

Models are also downloadable from https://figshare.com/s/b14c8e6cb1fc5135dd87. To implement an integrated model of division and growth, we built on a previously published tissue-level model for wild-type leaf growth at early stages of development [16]. This model has two interconnected networks: the Polarity Regulatory Network specifies tissue polarity and hence specified orientations of growth, and the Growth Regulatory Network (KRN) determines how factors influence specified growth rates. Specified growth orientations are established in relation to a polarity field, determined by the local gradient of a factor determining polarity field (POL) that propagates through the tissue, termed the canvas. The resultant growth and shape depend on the specified growth rates parallel (*K*_*par*_) and perpendicular (*K*_*per*_) to the polarity, and the mechanical constraints arising from the connectedness of the tissue.

In the equations, factors are denoted by **i** subscripted with the factor name. For instance, the factor PGRAD is described by **i**_*pgrad*_ in the equations. Factors may promote growth rates through the linear function *pro*, defined as follows:

$$pro(p_{f},\;i_{f})=1+p_{f}i_{f}$$

where **i**_*f*_ is a factor, F, and *p_f_* is a promotion coefficient for that factor. Factors may inhibit growth through the function *inh*, defined as follows:

$$inh\left(h_{f},\;i_{f}\right)=1/\left(1+h_{f}i_{f}\right)$$

where *h*_*f*_ is a inhibition coefficient for factor F. All multiplications and divisions are elementwise.

The previously proposed tissue-level growth model [16] was based on tracking only a subset of cell vertices and therefore had a lower cellular resolution than the data presented in this paper. Based on the higher resolution the cell fate map of the midline region of wild type and *spch* (S2 Fig), we widened the initial MID domain (S15 Fig) so that it gave a better match to the cellular data. Running the model with this change produced a narrower leaf, as MID inhibits *K*_*per*_. To compensate for this effect and to account for the regions with high growth rate perpendicular to the midline (Fig 1D and S1B Fig), we promoted *K*_*per*_ with PMF.

### S15 Fig—Model setup

The initial starting canvas for all models consists of 3,000 finite elements, which are not subdivided during the simulations, and model time is aligned with days after initiation (DAI), which is defined based on growth curves of leaf width [16]. To give finer resolution, times are given in hours (hours after initiation [HAI]). A list of growth parameter values is given in Table 1.

**Table 1:** Model parameters.

Parameter	Description	Value
**Polarity parameters**		
*b*_*pol*_	maximum POL levels	0.1
μ_*pol*_	POL decay rate	0.1 h^−1^
**Growth parameters**		
*b*_*pgrad*_	minimum levels of PGRAD	0.195
*g*_*late*_	increase in LATE over time	0.0048 h^−1^
*p*_*pgrad*_	*K*_*par*_ promotion by PGRAD	0.041 h^−1^
*p*_*lam*_	*K*_*per*_ promotion by LAM	0.0235 h^−1^
*p*_*late*_	*K*_*per*_ promotion by LATE	0.7
*h*_*late*_	*K*_*par*_ inhibition by LATE	2.2
*h*_*mid*_	*K*_*per*_ inhibition by MID	1.0
μ_*pmf*_	PMF decay rate	0.3 h^−1^
*d*_*pmf*_	Diffusion rate of PMF	0.01
*p*_*pmf*_	*K*_*per*_ promotion by PMF	0.9

Specified growth rates are modulated by a set of overlapping regional factors, PGRAD, MID, and LAM, the concentrations of which are fixed to the canvas and deform with it during growth (S15A Fig). PGRAD declines distally and accounts for the proximodistal variation in growth rate parallel to the polarity. LAM is expressed highest in the presumptive lamina and at lower levels in the distal regions that will form the petiole. LAM promotes growth perpendicular to polarity. MID is expressed in the midline region, as shown in S15A Fig, and inhibits growth perpendicular to the polarity. The maximum value of these factors is 1. Specified growth rates are also modulated by diffusible factor PMF, which is fixed to a value of 1 at the approximate position of the lamina-petiole boundary and allowed to diffuse through growth with a diffusion rate of *d*_*pmf*_ and a decay rate of μ_*pmf*_, giving the distribution shown in S15 Fig.

A temporally varying factor, LATE, is activated throughout the canvas to decrease growth at later stages. The value of LATE is initially 0 but rises linearly with time after 149 h:

$$i_{late}\left\{ \begin{array}{c} \;0\;if\;t<148\;h\\ g_{late}(t-148\;h)\;if\;t\geq 148\;h \end{array}\right.$$

where *g*_*late*_ defines the increase of LATE with time. LATE inhibits specified growth rates with an inhibition coefficient of *h*_*late*_.

### Polarity regulatory network

Polarity is established using factor PROXORG, which is set to 1 at the base of the canvas and 0 elsewhere (S15A Fig). The value of POL is fixed at a value of *b*_*pol*_, where PROXORG is greater than zero. POL diffuses throughout the canvas with a diffusion rate of *D*_*pol*_ and a decay rate of μ_*pol*_. POL distribution is allowed to establish during the setup phase for 20 time steps before the commencement of growth. Polarity is initially proximodistal and then deforms with the canvas as it grows to its final shape.

### Fig 8E and 8F—*spch* subepidermis model

This is a model for *spch* subepidermis during early stages of development.

#### KRN

Specified growth rates parallel to the polarity field *K*_*par*_ are defined as follows:

$$K_{par}=p_{pgrad}\;{¡}_{pgrad}.inh\left(h_{late},{¡}_{late}\right)$$

where *p*_*pgrad*_ is the promotion of growth by PGRAD.

Specified growth perpendicular to the polarity field *K*_*per*_ is defined as:

$$\begin{array}{l} K_{per}=p_{lam}{¡}_{lam}.pro\left(p_{late},{¡}_{late}\right)\\ \;.inh\left(h_{mid},{¡}_{mid}\right)\\ \;.pro(p_{pmf},{¡}_{pmftk}) \end{array}$$

where *p*_*lam*_ is the promotion of growth by LAM. The value of PMFTK is set to the value of PMF except where the value of PMF ≥ 0.295, in which case the value PMFTK is capped at 0.295. The canvas grows for the period 87 h < *t* < 178 h.

### v-Cells

To incorporate cell divisions within our tissue-level model, we superimposed polygons on the initial canvas to represent cells (S15A Fig, right). The sizes and geometries of these v-cells are based on cells observed at corresponding stages in confocal images of leaf primordia [16]. The vertices of the v-cells are anchored to the canvas and displaced with it during growth. New vertices are introduced as v-cells divide, according to the shortest wall passing through the centre of the v-cell [29]. Calling this the nominal new wall, the actual new wall is chosen to be parallel to this, through a point that is randomly displaced from the midpoint of the nominal new wall. The displacement is a vector chosen uniformly at random from a disc centred on the midpoint. The radius of this disc is 0.25 times the length of the nominal new wall. The length of the new wall is shortened slightly to give more realistic wall angles [79].

Cell divisions are determined through controlling competence and *Ā*.

#### Competence

Competence was determined by factor CDIV. v-cells can only divide if CDIV > 0. Factor CDIV was set to a value of 1 in the zone in which v-cells are competent to divide. The value of CDIV was set to 1 where the value of PMF ≥ 0.184, which corresponds to the value of PMF at about 400 μm^2^ from the petiole-lamina boundary. v-cells within the CDIV zone divide when they reach an area threshold, while v-cells outside the CDIV-expressing zone do not divide, regardless of their size. CDIV is turned off throughout the canvas when LATE reaches a threshold value of 0.1680 at 183 h.

#### Control of *A*

At cell birth, the cell is assigned a target area, *A*, equal to the mean target area *Ā* plus a random variation with standard deviation *σ* = 0.2*Ā*, in accordance with observed values of *σ* (Fig 2F). Before 114 h, we assume *Ā* = *A*_min_. After 114 h in the lamina, *Ā* ranges from 150 μm^2^ to 300 μm^2^ according to the value of PMF. For PMF values above *PMF*_*max*_, *Ā* remains at *A*_min_. As the value of PMF falls below *PMF*_*max*_, *Ā* rises linearly to *A*_max_ according to the formula:

Ā{AminAmin(1−α)+Amaxαt<114ht≥114h

where

$$\alpha =\frac{\left({PMF}_{max}-i_{pmf}\right)}{\left({PMF}_{max}-{PMF}_{min}\right)}$$

In the midline region,

Ā{Amin1.5Amidipgradt<114ht≥114h

In the Fig 8E and 8F*—spch* subepidermis model, *PMF*_*max*_ = 0.51, *PMF*_*min*_ = 0.184, *A*_*min*_ = 150 μm^2^, *A*_*max*_ = 300 μm^2^, and *A*_*mid*_ = 500 μm^2^.

#### Intercellular spaces

For the subepidermal model, formation of intercellular spaces was simulated by replacing a random selection of cell vertices with small empty equilateral triangles (with an initial area of 2 μm^2^). The number of initial intercellular spaces and the rate of additional spaces were calculated using the ratio of cells to intercellular spaces, in accordance with the experimental data. This resulted in 30 intercellular spaces being introduced at 120 h, and further replacements were made at a rate of 11 h^−1^. The vertices of the intercellular spaces moved away from their centres at a rate of 1.25% of their length h^−1^, equivalent to an areal growth rate of 2.5% h^−1^.

### Fig 8G and 8H—*spch* epidermis model

This is a model for *spch* epidermis during early stages of development.

#### KRN

Specified growth rates parallel to the polarity field, *K*_*par*_, and perpendicular to the polarity field, *K*_*per*_, were defined as in “Fig 8E and 8F—*spch* subepidermis model”.

The canvas grew for the period 87 h < *t* < 178 h.

#### v-Cells

v-cells were implemented as in “Fig 8E and 8F—*spch* subepidermis model,” but with the following modifications:

CDIV was set to 1 when the value of PMF ≥ *PMF*_*min*_ = 0.295 (compared with *PMF*_*min*_ = 0.184 for the subepidermis model), which shifts the distal boundary from about 400 μm to about 300 μm from the petiole-lamina boundary. Additionally, in order to model the initial restriction of cell division to proximal regions, CDIV was also set to 0 where PGRAD < 0.628.

*Ā* in the lamina was set using the same equation as the Fig 8E and 8F—*spch* subepidermis model (but with *PMF*_*min*_ = 0.295, as described in the previous paragraph). In the midline region, *Ā* was set to a constant value *A*_*mid*_ = 500 μm^2^ and did not vary with PGRAD as in “Fig 8E and 8F—*spch* subepidermis model.”

Ā{AminAmidt<114ht≥114h

Cells in the midline stopped dividing earlier than in the Fig 8E and 8F—*spch* subepidermis model and this was achieved in this model by reducing the LATE threshold in the midline to 0.0768 (compared with 0.1680).

### Fig 9B—Later stage mature *spch* epidermis model

This model extends the Fig 8G and 8H—*spch* epidermis model to later stages of development.

A new factor, EARLYGROWTH, was introduced in the model setup and set to a value of 1 throughout the canvas. After 189 h, EARLYGROWTH decreases linearly by a value of 0.0417 h^−1^ until it reaches a minimum value of 0.

$$i_{earlygrowth}\;\left\{ \begin{array}{l} \;1\;if\;t<189\;h\\ \;i_{earlygrowth}-0.0417\;(t-189)\;if\;t\geq 189\;h \end{array}\right.$$

To arrest growth, we modified factor LATE to increase exponentially after 189 h:

$$i_{late}\;\left\{ \begin{array}{l} \;0\;if\;t<148\;h\\ \;g_{late}(t-148\;h)\;if\;189\;h>t\geq 148\;h\\ \;{A\;e}^{Bt}\;if\;t\geq 189\;h \end{array}\right.$$

where *A = g*_*late*_ *(189–148) e*^*−B 189*^ and *B = 1 / (189–148)*. This ensures **i**_*late*_ evaluates to *g*_*late*_ *(t– 148)* at 189 h.

#### KRN

Changes relative to the Fig 8G and 8H—*spch* epidermis model are shown underlined.

Specified growth rate parallel to the polarity field *K*_*par*_ was modified with an additional term that led to inhibition after EARLYGROWTH starts to decline (at 189 h), as follows:

$$K_{par}=p_{pgrad}{¡}_{pgrad}.inh\left(h_{late},{¡}_{late}\right).\underline{inh\left(0.24,{¡}_{late}.\left(1‑{¡}_{earlygrowth}\right)\right)}$$

Specified growth perpendicular to the polarity field *K*_*per*_ was modified with additional terms that led to loss of promotion by LATE and inhibition after EARLYGROWTH starts to decline (at 189 h), as follows:

$$\begin{array}{l} K_{per}=p_{lam}{¡}_{lam}\\ \;.inh\left(h_{mid},{¡}_{mid}\right)\\ \;.pro\left(p_{pmf},{¡}_{pmftk}\right)\\ \;.pro(p_{late},{¡}_{late}\underline{.{¡}_{earlygrowth})}\\ \;\underline{.inh\left(2.8,{¡}_{late}.\left(1‑{¡}_{earlygrowth}\right)\right)} \end{array}$$

The canvas grows for the period 87 h < *t* < 412 h.

#### v-Cells

v-cells were defined as in “Fig 8G and 8H—*spch* epidermis model”.

### Fig 9C—Later stage *spch* limit-free epidermis model

This model inhibits distal growth during later stages in order to reduce the size of distal cells. It does this by using the existing factors PGRAD and LAM.

#### KRN

Changes relative to the Fig 8G and 8H—*spch* epidermis model are shown underlined. Specified growth rates parallel to the polarity field *K*_*par*_ were modified by introducing inhibitory factors that increase distally. As both PGRAD and LAM values decrease distally from a maximum of 1, by subtracting their values from 1, we obtain factors that increase distally. These distally increasing factors inhibited growth as EARLYGROWTH decreased. As this introduces extra growth inhibition, this was compensated for by also inhibiting growth inhibition by LATE as EARLYGROWTH decreased:

$$\begin{array}{l} K_{par}=P_{pgrad}{¡}_{pgrad}\\ \;.inh(h_{late},{¡}_{late}.\underline{inh\left(1.5,\left(1‑{¡}_{earlygrowth}\right)\right)}\\ \;\underline{.inh\left(2,\left(1‑{¡}_{lam}\right).\left(1‑{¡}_{earlygrowth}\right)\right)}\\ \;\underline{.inh\left(4,\left(1‑{¡}_{pgrad}\right).\left(1‑{¡}_{earlygrowth}\right)\right)} \end{array}$$

Specified growth perpendicular to the polarity field *K*_*per*_ was modulated in a similar way as *K*_*par*_ described above:

$$\begin{array}{l} K_{per}=p_{lam}{¡}_{lam}\\ \;.inh\left(h_{mid},{¡}_{mid}\right)\\ \;.pro\left(p_{pmf},{¡}_{pmftk}\right)\\ \;.pro(p_{late},{¡}_{late}\underline{.{¡}_{earlygrowth})}\\ \;\underline{.inh\left(1.2,{¡}_{late}.\left(1‑{¡}_{earlygrowth}\right)\right)}\\ \;\underline{.inh\left(4,\left(1‑{¡}_{pgrad}\right).\left(1‑{¡}_{earlygrowth}\right)\right)} \end{array}$$

The canvas grows for the period 87 h < *t* < 412 h.

#### v-Cells

v-cells were implemented as in the Fig 8G and 8H—*spch* epidermis model.

### Fig 9J—Later stage *spch* limiting cell size epidermis model

In this model, cell size can affect growth. This is an alternative way to limit the size of distal cells without having to use the factors PGRAD and LAM, as in the Fig 9C—Later stage *spch* limit-free epidermis model.

#### KRN

Specified growth rates were defined as in the Fig 9B—Later stage mature *spch* epidermis model, but with a v-cell size feedback on growth affecting *K*_*per*_ and *K*_*par*_. When cell area, *ca*, exceeds a threshold size *a*_*1*_, specified growth rates (*K*_*par*_ and *K*_*per*_) start to be reduced by *ω* in proportion to cell area until cell size reaches *a*_*2*_, after which specified growth rates are set to zero.

$$K_{par}=\underline{\omega .}p_{pgrad}{¡}_{pgrad}.inh\left(h_{late},{¡}_{late}\right).inh\left(0.24,{¡}_{late}.\left(1‑{¡}_{earlygrowth}\right)\right)$$

$$\begin{array}{l} K_{per}=\underline{\omega }\\ \;.p_{lam}{¡}_{lam}\\ \;.inh\left(h_{mid},{¡}_{mid}\right)\\ \;.pro\left(p_{pmf},{¡}_{pmftk}\right)\\ \;.pro\left(p_{late},{¡}_{late}.{¡}_{earlygrowth}\right)\\ \;.inh\left(2.8,{¡}_{late}.\left(1‑{¡}_{earlygrowth}\right)\right) \end{array}$$

where

$$\omega =\frac{a_{2}-ca}{a_{2}-a_{1}}$$

In the lamina region, *a*_*1*_ = 4,000 μm^2^ and *a*_*2*_ = 8,000 μm^2^, while in the midline region, *a*_*1*_ = 18,000 μm^2^ and *a*_*2*_ = 20,000 μm^2^.

All other v-cell properties were as in “Fig 9B—Later stage mature *spch* epidermis model.”

### Fig 9D and 9E and 9K–9L—Cell division threshold mutant models

Cell division threshold models were developed for the Fig 9C—later stage *spch* limit-free epidermis model and Fig 9J—later stage *spch* limiting cell size epidermis model. Each of the cell division models was identical to its parent model, but the cell target area for division, *Ā*, was increased by a constant *a'* for t ≥ 114 h.

$$\bar{A}=\left({\bar{A}}_{min}\left(1-\alpha \right)+{\bar{A}}_{max}\;\alpha \right)\;\underline{+\;a'}$$

In Fig 9D and 9K, *a'* = 85 μm^2^, while in Fig 9E and 9L, *a'* = −85 μm^2^.

### Fig 9F and 9G and 9M–9N—Growth rate mutant models

Growth rate mutant models were developed for the Fig 9C—later stage *spch* limit-free epidermis model and the Fig 9J—later stage *spch* limiting cell size epidermis model. Each of the growth rate mutant models was identical to its parent model, but *K*_*per*_ and *K*_*par*_ were globally scaled by a factor *k'*. In Fig 9F and 9M, *k'* = 0.95, while in Fig 9G and 9N, *k'* = 1.05.

For the Fig 9C—later stage *spch* limit-free epidermis model:

$$\begin{array}{l} K_{par}=\underline{k'}\\ \;.p_{pgrad}{¡}_{pgrad}\\ \;.inh(h_{late},{¡}_{late}.inh\left(1.5,\left(1‑{¡}_{earlygrowth}\right)\right)\\ \;.inh\left(2,\left(1‑{¡}_{lam}\right).\left(1‑{¡}_{earlygrowth}\right)\right)\\ \;.inh\left(4,\left(1‑{¡}_{pgrad}\right).\left(1‑{¡}_{earlygrowth}\right)\right)\\ \; \end{array}$$

$$\begin{array}{l} K_{per}=\underline{k'}\\ \;.p_{lam}{¡}_{lam}\\ \;.inh\left(h_{mid},{¡}_{mid}\right)\\ \;.pro\left(p_{pmf},{¡}_{pmftk}\right)\\ \;.pro\left(p_{late},{¡}_{late}.{¡}_{earlygrowth}\right)\\ \;.inh\left(1.2,{¡}_{late}.\left(1‑{¡}_{earlygrowth}\right)\right)\\ \;.inh\left(4,\left(1‑{¡}_{pgrad}\right).\left(1‑{¡}_{earlygrowth}\right)\right) \end{array}$$

For the Fig 9J—later stage *spch* limiting cell size epidermis model:

$$K_{par}=\underline{k'}.\omega .P_{pgrad}{¡}_{pgrad}.inh\left(h_{late},{¡}_{late}\right).inh\left(0.24,{¡}_{late}.\left(1‑{¡}_{earlygrowth}\right)\right)$$

$$\begin{array}{l} K_{per}=\underline{k'}\\ \;.\omega \\ \;.P_{lam}{¡}_{lam}\\ \;.inh\left(h_{mid},{¡}_{mid}\right)\\ \;.pro\left(P_{pmf},{¡}_{pmftk}\right)\\ \;.pro\left(P_{late},{¡}_{late}.{¡}_{earlygrowth}\right)\\ \;.inh\left(2.8,{¡}_{late}.\left(1‑{¡}_{earlygrowth}\right)\right) \end{array}$$

Changes to models relative to those used to generate Fig 9C and Fig 9J are shown underlined.

### Fig 9H, 9I, 9O and 9P—LATE mutant models

Models were developed for the Fig 9C—later stage *spch* limit-free epidermis model and Fig 9J—later stage *spch* limiting cell size epidermis model. Each of the LATE mutant models was identical to its parent model, but the activation of LATE and EARLYGROWTH was shifted by a constant number of hours, *t′*. In Fig 9H and 9O, *t'* = −6 h, while in Fig 9I and 9P, *t'* = 6 h.

$$i_{late}\{\begin{array}{l} \;0\;if\;t<148\;h\underline{+t\prime }\\ g_{late}\left(t-148\;h\underline{+t\prime }\right)\;if\;189\;h\underline{+t\prime }>t\geq 148h\underline{+t\prime }\\ \;A\;e^{Bt}\;if\;t\geq 189\;h\underline{+t\prime } \end{array}$$

where *A = g*_*late*_. *(189*+*t*′ – 148+*t*′). e^*-B 189*+*t'*^ and *B = 1 / (189*+*t'* – 148+*t'*).

$$i_{earlygrowth}\left\{ \begin{array}{l} \;1\;if\;t<189\;h\underline{+t\prime }\\ \;i_{earlygrowth}-0.0417\;(t-189)\;if\;t\geq 189\;h\underline{+t\prime } \end{array}\right.$$

Changes to models relative to those used to generate Fig 9C and Fig 9J are shown underlined.

### Fig 10—Later stage mature *spch* epidermis model

In the above models, *t* refers to actual time. We modified the late stage *spch* epidermis model (as used for Fig 9B) by setting physiological time to be a constant fraction (physiological ratio) of duration since the start of the simulation (when *t* = 87 h). Key transitions and growth rates were then set in relation to physiological time. Parameters describing physical processes such as diffusion were left unchanged. For the model for *spch* grown on plates, the physiological ratio was 0.55. In addition, the growth rates were globally scaled by a factor *k'* = 0.6 The net result of these two changes is that growth in actual time is slowed to 0.33 of normal. If this overall growth rate was matched purely by changing physiological time, the leaf on the plate would end up larger than observed at maturity. Conversely, if the growth rate was matched purely through changes in *k'*, the leaf would end up much smaller than observed for *spch* on plates at maturity. Thus, changes in both physiological time and *k'* are needed to match the observed growth curve.

### S14C Fig—*spch* in chamber tuned to lower growth rate at later stages

The only change in relation to the late stage *spch* epidermis model (as used for Fig 9B) was that the physiological ratio (as defined above) was set to 0.75.

## Supporting information

S1 Fig Scatterplots of growth rates parallel and perpendicular to the midline in the *spch* epidermis. From the time-lapse imaging experiment shown in Fig 1. (**A**) Growth rates parallel to the midline (*K*_*Midline*_) versus distance from the petiole-lamina boundary. (**B**) Growth rates perpendicular to the midline(*K*_*PerMidline*_) versus distance from the centre of the midline. Data points are colour coded according to tracking interval (inset). Source data are available from https://figshare.com/s/b14c8e6cb1fc5135dd87.
(TIF)

S2 Fig Assignment of the midline and lamina regions. From the time-lapse imaging experiment shown in Fig 1. Lineages were traced from cells visible at the beginning of the experiment (0 h, left) through to the end (74 h, right) and were assigned an arbitrary colour, ensuring neighbours were coloured differently. Cells in the midline (within the black outline) were identified using the position and shape of clones (cells of the same lineage) in the final image, as was the approximate position of the distal end of the midline (dark grey). Cells outside the midline region were classified as being in the lamina. Scale bar = 100 μm.
(TIF)

S3 Fig Dynamics of cell division and growth in the *spch* epidermis, additional dataset. Time-lapse imaging of a *spch* leaf at approximately 24-h intervals over 4 d (0–96 h, last time point in series not shown). Data shown on the first time point (underlined) for each tracking interval. Leaf widths for first time point (left to right) are 0.17, 0.27, 0.39, and 0.50 mm. (**A**) Cells amenable to tracking that were competent to divide (green) and either executed division during the interval (light green) or divided in a later interval (dark green). Cells that did not divide (black, first row in 0–25 h are coloured orange throughout). (**B-D**) Cellular growth rates (heat maps) for each tracking interval. Black line refers to orange cells in (A). (**B**) Areal growth rates. (**C**) Growth rates parallel to the midline (proximodistal). (**D**) Growth rates perpendicular to the midline (mediolateral). (**E**) Cell areas for the first time point of the interval. Leaf outline indicated by dotted black line. The petiole-lamina boundary was defined as described in Fig 1. Grey boxes are aligned to the petiole-lamina boundary and extend to 150 or 300 *μ*m. Black arrows indicate distal boundary of the zone of division. Scale bar = 100 *μ*m. Source data are available from https://figshare.com/s/b14c8e6cb1fc5135dd87. *spch*, *speechless*.
(TIF)

S4 Fig Dynamics of cell division and growth in the *spch* epidermis, additional dataset. Time-lapse imaging of a *spch* leaf at approximately 24-h intervals over 5 d (0–118 h, last time point in series not shown). Data shown on first time point (underlined) for each tracking interval. Leaf widths for the first time point (left to right) are 0.23, 0.34, 0.48, 0.67, and 0.87 mm. (**A**) Cells amenable to tracking that were competent to divide (green) and either executed division during the interval (light green) or divided in a later interval (dark green). Cells that did not divide (black, first row in 0–23 h are coloured orange throughout). (**B-D**) Cellular growth rates (heat maps) for each tracking interval. Black line refers to orange cells in (A). (**B**) Areal growth rates. (**C**) Growth rates parallel to the midline (proximodistal). (**D**) Growth rates perpendicular to the midline (mediolateral). (**E**) Cell areas for the first time point of the interval. Leaf outline indicated by dotted black line. The petiole-lamina boundary was defined as described in Fig 1. Grey boxes are aligned to the petiole-lamina boundary and extend to 150 or 300 *μ*m. Black arrows indicate distal boundary of the zone of division. Scale bar = 100 *μ*m. Source data are available from https://figshare.com/s/b14c8e6cb1fc5135dd87. *spch*, *speechless*.
(TIF)

S5 Fig Analysis of epidermal cell size using 3D segmentation. (**A,B**) Data from a sample of cells in the tracking experiment shown in S3 Fig, 96 h. (**A**) Segmentation-based projected cell area versus vertex-based cell area (*R*^2^ = 0.87, slope = 1.03, intercept = 125, standard deviation along y-axis = 72). (**B**) Segmentation-based cell surface area versus vertex-based cell area (*R*^2^ = 0.77, slope = 1.2, intercept = 145, standard deviation along y-axis = 115). (**C-F**) Data from a sample of cells in tracking experiment shown in Fig 1E, 58 h. (**C**) Cell volume versus segmentation-based cell surface area (*R*^2^ = 0.91, slope = 8.93). (**D**) Cell volume divided by cell surface area (cell thickness). (**E**) Segmentation-based cell surface area. (**F**) Cell volume. Scale bar = 100 *μ*m. For each heat map, the upper limit of the colour scale was set to 20-fold that of the lower limit. Source data are available from https://figshare.com/s/b14c8e6cb1fc5135dd87.
(TIF)

S6 Fig Cellular areal growth rates of dividing and nondividing cells in the spch epidermis. Time-lapse imaging of a *spch* leaf shown in Fig 1. (**A**) Panel repeated from Fig 1A, for ease of comparison. Cells amenable to tracking that were competent to divide (green) and either executed division during the interval (light green) or divided in a later interval (dark green). Cells that did not divide (black, first row in 0–14 h are coloured orange throughout). For the last interval (74–100 h), cell divisions could only be tracked for a subset of cells because of missing data in the 100-h time point. (**B-C**) Cellular growth rates (heat maps) shown in Fig 1B, separated to show (**B**) areal growth rates of nondividing cells, coloured black in (A). (**C**) Areal growth rates of dividing cells, coloured green in (A). Leaf outline indicated by dotted black line. The petiole-lamina boundary was defined by selecting a cell from a later stage of development, where the lamina narrows, and then tracing its lineage back to all stages. Grey boxes are aligned to the petiole-lamina boundary and extend to 150 or 300 *μ*m. Black arrows indicate distal boundary of the zone of division competence. Scale bar = 100 *μ*m. *spch*, *speechless*.
(TIF)

S7 Fig Cell division dynamics of the epidermis at high temporal resolution. Cells amenable to tracking from the time-lapse imaging experiment shown in Fig 2; data visualised over 24-h intervals, shown on the first image of the interval (underlined). (**A**) Cell cycle duration (heat map) for cells that were observed to complete a full cell cycle during the course of the experiment. Cells that did not divide or did not complete a full cell cycle are coloured black. (**B**) Cells that were competent to divide (green) and either executed division during the interval (light green) or divided in a later interval (dark green). Cells that did not divide (black). Leaf outline indicated by dotted black line. The petiole-lamina boundary was defined as described in Fig 1. Grey boxes are aligned to the petiole-lamina boundary and extend to 150 or 300 *μ*m. Scale bar = 100 μm.
(TIF)

S8 Fig Subepidermal cells in wild-type leaves. (**A**) Projections of the subepidermal layer of four individual wild-type leaves, fixed and stained at developmental stages, similar to those of the tracked *spch* leaf in Fig 5. Leaf widths (left to right) are 0.14, 0.28, 0.40, and 0.53 mm. A patch of cells was coloured red and used to look in further detail. (**B**) Enlargement of the patch of cells in (A) (red outline). (**C**) Cells outlined in (B), showing spacing of individual cells (filled pink, outlined red). (**D**) Epidermal cells adjacent to the subepidermal patch (subepidermal cells filled and outlined red). (**E**) Epidermal cells without subepidermal patch outlined. Scale bars = 50 *μ*m. Source data are available from https://figshare.com/s/b14c8e6cb1fc5135dd87. *spch*, *speechless*.
(TIF)

S9 Fig Comparison of epidermal and subepidermal cell areal growth rates. A group of cells from the time-lapse imaging experiment shown in Fig 5, subepidermis (cells highlighted red), and adjacent epidermis (dynamics of the epidermis shown in full in S3 Fig). Average cell areal growth rates for each tracking interval are shown on the first image of each interval (time point underlined). (**A**) Epidermis, (**B**) subepidermis. The patch of cells is also shown enlarged for comparison. (**C**) Epidermis. (**D**) Subepidermis, showing increase in number of cells through division compared to the epidermis. Numbers refer to number of cells. Scale bars = 50 *μ*m.
(TIF)

S10 Fig Comparison of the zone of competence in epidermal and subepidermal layers. Time-lapse imaging of a *spch* leaf at approximately 24-h intervals over 2 d (0–50 h); later time points of this experiment are shown in S3 Fig. Cells amenable to tracking are shown on the first time point (underlined). Cells that were competent to divide (green) and either executed division during the interval (light green) or divided in a later interval (dark green). Cells that did not divide (black). (**A**) Subepidermis. (**B**) Epidermis. Leaf outline indicated by dotted black line. Leaf widths from left to right are 0.17 and 0.27 mm. The petiole-lamina boundary was defined as described in Fig 1. Grey boxes are aligned to the petiole-lamina boundary and extend to 150 or 300 *μ*m. Scale bar = 100 *μ*m. *spch*, *speechless*.
(TIF)

S11 Fig Analysis of subepidermal cell size using 3D segmentation. Analysis of a sample of abaxial subepidermal cells from the tracking experiment shown in Fig 6, 58 h. (**A**) Projected segmentation-based cell area versus vertex-based area (*R*^2^ = 0.98, slope = 1.03, intercept = 46, standard deviation along y-axis = 20). (**B**) Cell volume versus projected segmentation-based cell area, subepidermal cells (blue, *R*^2^ = 0.94, slope = 15.4), epidermal cells (red). (**C**) Cell volume divided by projected segmentation-based area (approximate cell thickness). (**D**) Projected segmentation-based area. (**E**) Cell volume. For each heat map, the upper limit was set to 8-fold that of the lower limit. (**F**) Orthogonal slice of confocal image, approximately through the midline of the leaf. Thickness of epidermal and subepidermal cells appears approximately uniform from leaf base (left) to leaf tip (right). Subepidermal cells close to the distal leaf tip are difficult to resolve. Scale bars = 100 *μ*m. Source data are available from https://figshare.com/s/b14c8e6cb1fc5135dd87.
(TIF)

S12 Fig Comparison of wild-type and *spch* primordia. Confocal images of an early wild-type leaf primordium (left) and a *spch* primordium (right) at similar developmental stages. The leaf primordia are partially obscured by the petioles of cotyledon leaves. Scale bar = 50 μm. *spch*, *speechless*.
(TIF)

S13 Fig Quantification of cell area at division execution in wild type. Data from cells amenable to tracking in the time-lapse experiment shown in Fig 7. (**A,B**) Area of lamina cells at the time of division execution versus distance from the petiole-lamina boundary. Mean cell area at the division for all cells is 87 ± 6.0 *μ*m^*2*^. (**A**) Cells classified as non-stomatal lineage (mean = 165 ± 27.8 *μ*m^*2*^). (**B**) Cells classified as stomatal lineage (mean = 81 ± 5.7 *μ*m^*2*^). (**C,D**) Cell cycle duration for all cells observed to complete a cell cycle. (**C**) Non-stomatal lineage cells (mean = 24.6 ± 2.8 h). (**D**) Stomatal lineage cells (mean = 18.5 ± 1.05 h). ± ranges indicate 1.96 × standard error of mean. Data points are colour coded according to time interval (inset in A). Source data are available from https://figshare.com/s/b14c8e6cb1fc5135dd87.
(TIF)

S14 Fig Comparison of wild-type and *spch* growth rates in leaf width. (A) Width measurements of leaf 1 from *spch*, wild-type, and *fama* seedlings grown in standard conditions on plates. The fitted growth curve of wild type (solid black line) was based on a logistic calculation [16]. Pink line shows output leaf widths for the model. Blue line shows output leaf widths for the model tuned to match *spch* growth on plates (by slowing growth by 40% and physiological time by 45%). Horizontal line shows leaf width at about 0.5 mm, corresponding to stages shown in Fig 10. (B) Width measurements of leaf 1 from seedlings grown in the bio-imaging chamber and scanned using confocal microscopy. Measurements are from six independent tracking experiments, two wild-type individuals, and four *spch* individuals (colour key). Because *spch* plants grown prior to moving into the chamber have much-reduced growth (shown in B), the initial data point from each tracking experiment was normalised to the wild-type logistic curve (solid black line) to enable subsequent growth rates to be compared. Pink line shows output leaf widths for the model. Blue line shows output for the leaf model tuned to match *spch* growth in the chamber at later stages (by slowing physiological time by 25%). (C) Outputs for the final stage comparing the model (left) with that tuned to match *spch* growth in chamber (right). Source data are available from https://figshare.com/s/b14c8e6cb1fc5135dd87. *spch*, *speechless*.
(TIF)

S15 Fig Model setup and regulatory factors. **(A)** Initial canvas distribution of regulatory factors, from left to right: PGRAD (greyscale), LAM (pink) and MID (purple), POL (blue, with arrows indicating gradient), and initial pattern of v-cells. (**B**) Time series showing accumulation of LATE at 115, 124, 153, and 182 h. (**C**) Time series of PMF levels at 115, 124, 153, and 182 h. (**D**) Sample plot of PMF concentration along the leaf midline. Note PMF concentration is fixed at 1 around the petiole-lamina boundary (dotted line). LAM, a factor distinguishing lamina from petiole; LATE, a timing factor; MID, a mediolateral factor; PGRAD, a graded proximodistal factor; PMF, proximal mobile factor; POL, factor determining polarity field; v-cell, virtual cell.
(TIF)

S16 Fig Resultant growth rates for model output at different developmental stages. Model output (from left to right) at 115, 132, 140, 147, 156, 164, and 178 h showing (**A**) resultant areal growth rates, (**B**) resultant growth rates parallel to the midline (proximodistal growth rates), and (**C**) resultant growth rates perpendicular to the midline (mediolateral growth rates). Grey boxes are aligned to the petiole-lamina boundary and extend to 150 or 300 *μ*m. Scale bar = 100 μm.
(TIF)

S17 Fig Topological distributions (frequency of *n*-sided cells) from empirical data and different models. **(A)** Dark green line shows *Arabidopsis spch* epidermis with standard deviation between leaves for the fraction of *n*-sided cells indicated by a vertical line. Data are based on 42,901 cells from 99 *spch* leaves at different stages of development. The frequency of six-sided cells is 0.334 ± 0.019 (SD). Black line shows the result of the model of *spch* epidermis presented here. Grey line shows the model without polarity (isotropic specified growth). Light green line shows empirical data from the shoot apical meristem [31]. Orange line shows the model using the shortest path through the cell’s centroid within an isotropically growing tissue [31]. Dark blue line shows a graph-model in which cells divide through the topological centre [30]. **(B-D)** Tissue topology with cells coloured according to their neighbourhood number, with six-sided cells in white, higher numbers in green, and lower numbers in brown, as shown in the colour scale. **(B)** Result for the model of *spch* epidermis presented here (black profile in A); **(C)** model without polarity, yielding an isotropically growing tissue (grey profile in A). **(D)** Example of *spch* leaf, as shown in S18D Fig. Source data are available from https://figshare.com/s/b14c8e6cb1fc5135dd87. *spch*, *speechless*.
(TIF)

S18 Fig Additional comparisons between chamber- and plate-grown *spch* leaves. Supporting data for Fig 10; *spch* leaf grown in the chamber at 8 DAS (A-B) or grown on plates at 13 DAS (C-D) at similar leaf widths to leaves presented in Fig 10. **(A, C)** Confocal images of leaves, with segmentation outlines overlain in blue. **(B, D)** Analysis of segmentations in (A, C) showing (from left to right) neighbourhood numbers (following neighbourhood colour map used in S18B Fig); cell complexity based on the CD measure using LOCO-EFA (using complexity colour scale as in Fig 10K–10N); and absolute areas (using colour scale as in Fig 10A and 10B). Results are consistent with those described for Fig 10. Scale bars = 100 μm. Source data are available from https://figshare.com/s/b14c8e6cb1fc5135dd87. CD, cumulative difference; LOCO-EFA, Lobe-Contribution Elliptic Fourier Analysis; *spch*, *speechless*.
(TIF)

S1 Video Cell division in the stomatal lineage. Magnification of a cell undergoing three rounds of amplifying divisions, from time-lapse imaging of a wild-type leaf (expressing *pSPCH*:*SPCH-GFP*, shown in Fig 7). Bright, nuclear-localised SPCH can be observed at the time of division and is retained in one daughter cell until the next division. Scale bar = 10 *μ*m. Time interval, 1 h. Duration of video, 57 h. Images from time points 18–24 h are missing because of movement of the microscope stage. SPCH, SPEECHLESS.
(AVI)

S2 Video Cell division not in the stomatal lineage. Time-lapse movie of a cell undergoing a single, non-stomatal division, from time-lapse imaging of a wild-type leaf (expressing *pSPCH*:*SPCH-GFP*, shown in Fig 7). Weak, nuclear-localised SPCH can be observed at the time of division and disappears immediately after division in both daughter cells. Scale bar = 10 *μ*m. Time interval, 1 h. Duration of video, 57 h. Images from time points 18–24 h are missing because of movement of the microscope stage. SPCH, SPEECHLESS.
(AVI)

S3 Video Subepidermal growth model. Stages of the model used to generate Fig 8E: growth and divisions of the subepidermal layer during early stages.
(MP4)

S4 Video Epidermal growth model. Stages of the model used to generate Fig 8G: growth and divisions of the epidermal layer during early stages.
(MP4)

S5 Video Epidermal growth model to maturity. Stages of the model used to generate Fig 9B: growth and divisions of the epidermal layer through to maturity.
(MP4)

S6 Video Epidermal growth model to maturity, with limiting cell size. Stages of the model used to generate Fig 9J: growth and divisions of the epidermal layer through to maturity, with feedback of cell size limits on growth.
(MP4)

S7 Video Epidermal growth model to maturity, limit-free. Stages of the model used to generate Fig 9C: growth and divisions of the epidermal layer through to maturity, with distal inhibition of growth.
(MP4)

## Abbreviations

Ā: mean threshold cell area for division execution
CD: cumulative difference
CDIV: competence to divide
CER: controlled environment room
cyc1At-GUS: *cyclin1 Arabidopsis thaliana* β-glucuronidase
DAI: day after initiation
HAI: hour after initiation
FAMA: basic helix-loop-helix transcription factor (bHLH097
KLUH: *Arabidopsis* cytochrome P450/*CYP78A5*
K_par_: specified growth rate parallel to polarity axis
K_per_: specified growth rate perpendicular to polarity axis
KRN: growth regulatory network
LAM: a factor distinguishing lamina from petiole
LATE: a timing factor
LOCO-EFA: Lobe-Contribution Elliptic Fourier Analysis
MID: a mediolateral factor
mPS-PI: modified pseudo-Schiff propidium iodide
PGRAD: a graded proximodistal factor
PMF: proximal mobile factor
POL: factor determining polarity field
SDS: Sodium Dodecyl Sulfate
spch: *speechless*
v-cell: virtual cell
