# Supplementary figures and images for: Spatiotemporal coordination of cell division and growth during organ morphogenesis
Source: PLoS Biol. 2018 Nov 1;16(11):e2005952. doi: 10.1371/journal.pbio.2005952 (PMC6211367; doi:10.1371/journal.pbio.2005952)

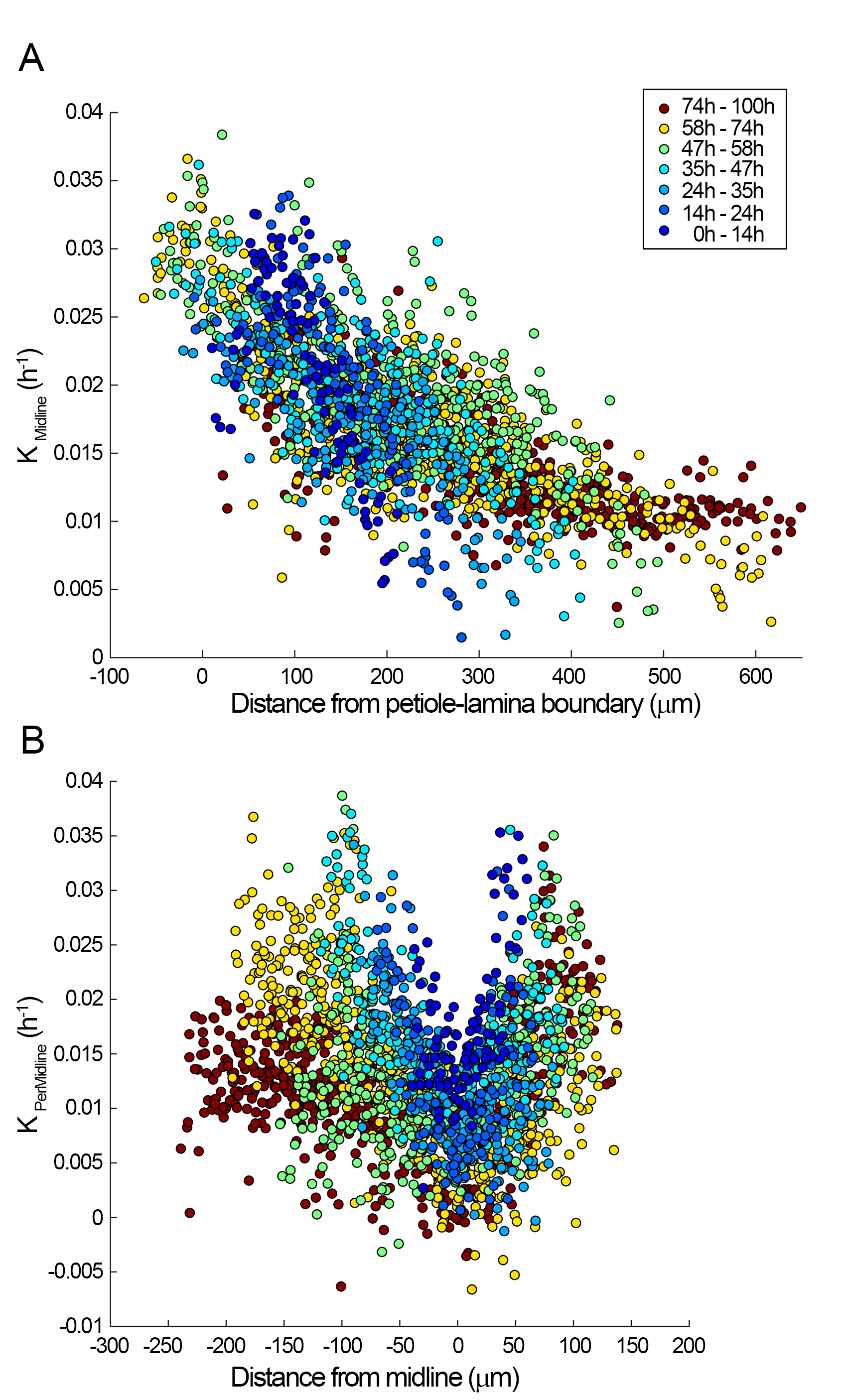

Supplement: S1 Fig — From the time-lapse imaging experiment shown in Fig 1. (A) Growth rates parallel to the midline (KMidline) versus distance from the petiole-lamina boundary. (B) Growth rates perpendicular to the midline(KPerMidline) versus distance from the centre of the midline. Data points are colour coded according to tracking interval (inset). Source data are available from https://figshare.com/s/b14c8e6cb1fc5135dd87. (TIF) [file pbio.2005952.s001.tif]

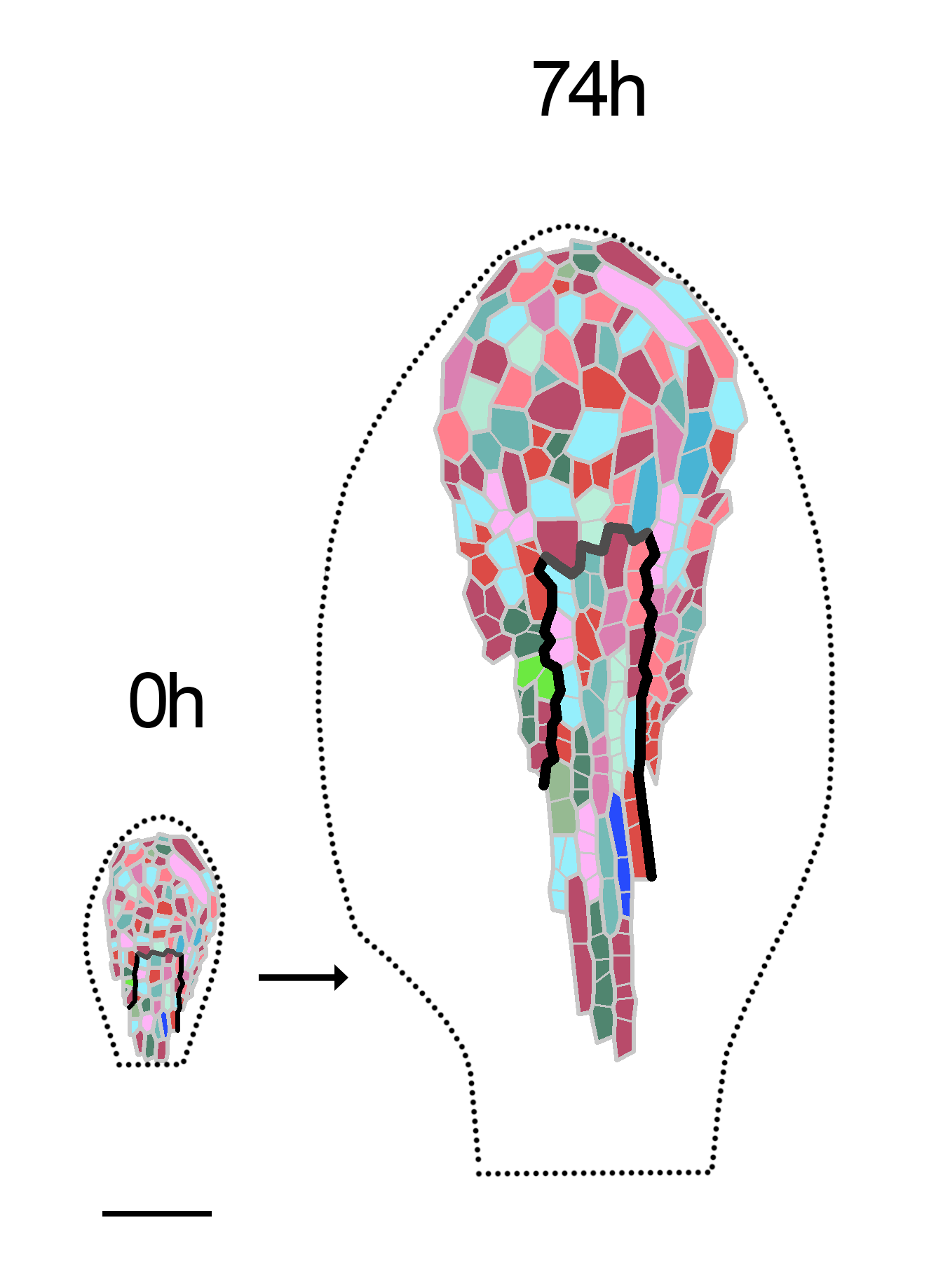

Supplement: S2 Fig — From the time-lapse imaging experiment shown in Fig 1. Lineages were traced from cells visible at the beginning of the experiment (0 h, left) through to the end (74 h, right) and were assigned an arbitrary colour, ensuring neighbours were coloured differently. Cells in the midline (within the black outline) were identified using the position and shape of clones (cells of the same lineage) in the final image, as was the approximate position of the distal end of the midline (dark grey). Cells outside the midline region were classified as being in the lamina. Scale bar = 100 μm. (TIF) [file pbio.2005952.s002.tif]

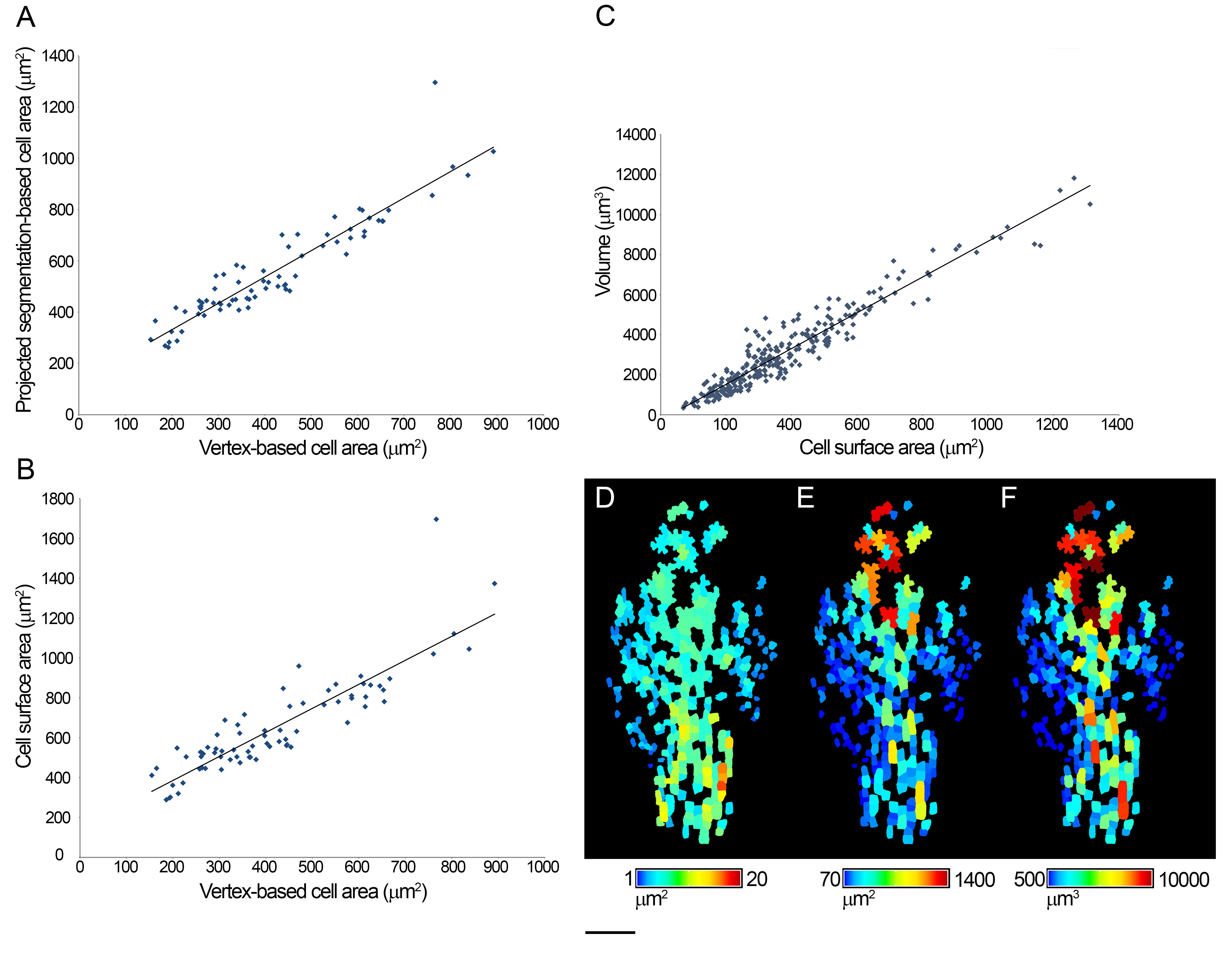

Supplement: S5 Fig — (A,B) Data from a sample of cells in the tracking experiment shown in S3 Fig, 96 h. (A) Segmentation-based projected cell area versus vertex-based cell area (R2 = 0.87, slope = 1.03, intercept = 125, standard deviation along y-axis = 72). (B) Segmentation-based cell surface area versus vertex-based cell area (R2 = 0.77, slope = 1.2, intercept = 145, standard deviation along y-axis = 115). (C-F) Data from a sample of cells in tracking experiment shown in Fig 1E, 58 h. (C) Cell volume versus segmentation-based cell surface area (R2 = 0.91, slope = 8.93). (D) Cell volume divided by cell surface area (cell thickness). (E) Segmentation-based cell surface area. (F) Cell volume. Scale bar = 100 μm. For each heat map, the upper limit of the colour scale was set to 20-fold that of the lower limit. Source data are available from https://figshare.com/s/b14c8e6cb1fc5135dd87. (TIF) [file pbio.2005952.s005.tif]

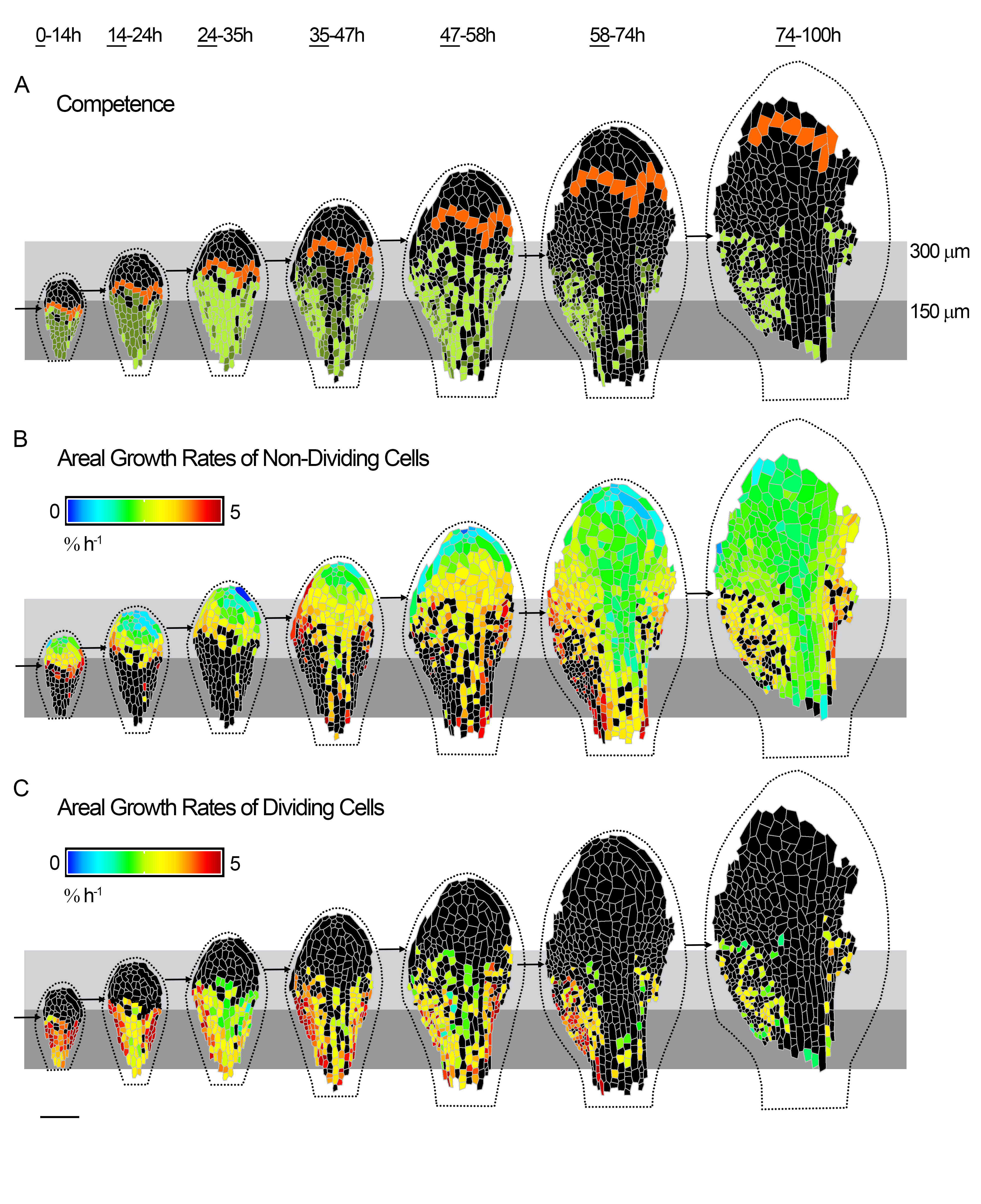

Supplement: S6 Fig — Time-lapse imaging of a spch leaf shown in Fig 1. (A) Panel repeated from Fig 1A, for ease of comparison. Cells amenable to tracking that were competent to divide (green) and either executed division during the interval (light green) or divided in a later interval (dark green). Cells that did not divide (black, first row in 0–14 h are coloured orange throughout). For the last interval (74–100 h), cell divisions could only be tracked for a subset of cells because of missing data in the 100-h time point. (B-C) Cellular growth rates (heat maps) shown in Fig 1B, separated to show (B) areal growth rates of nondividing cells, coloured black in (A). (C) Areal growth rates of dividing cells, coloured green in (A). Leaf outline indicated by dotted black line. The petiole-lamina boundary was defined by selecting a cell from a later stage of development, where the lamina narrows, and then tracing its lineage back to all stages. Grey boxes are aligned to the petiole-lamina boundary and extend to 150 or 300 μm. Black arrows indicate distal boundary of the zone of division competence. Scale bar = 100 μm. spch, speechless. (TIF) [file pbio.2005952.s006.tif]

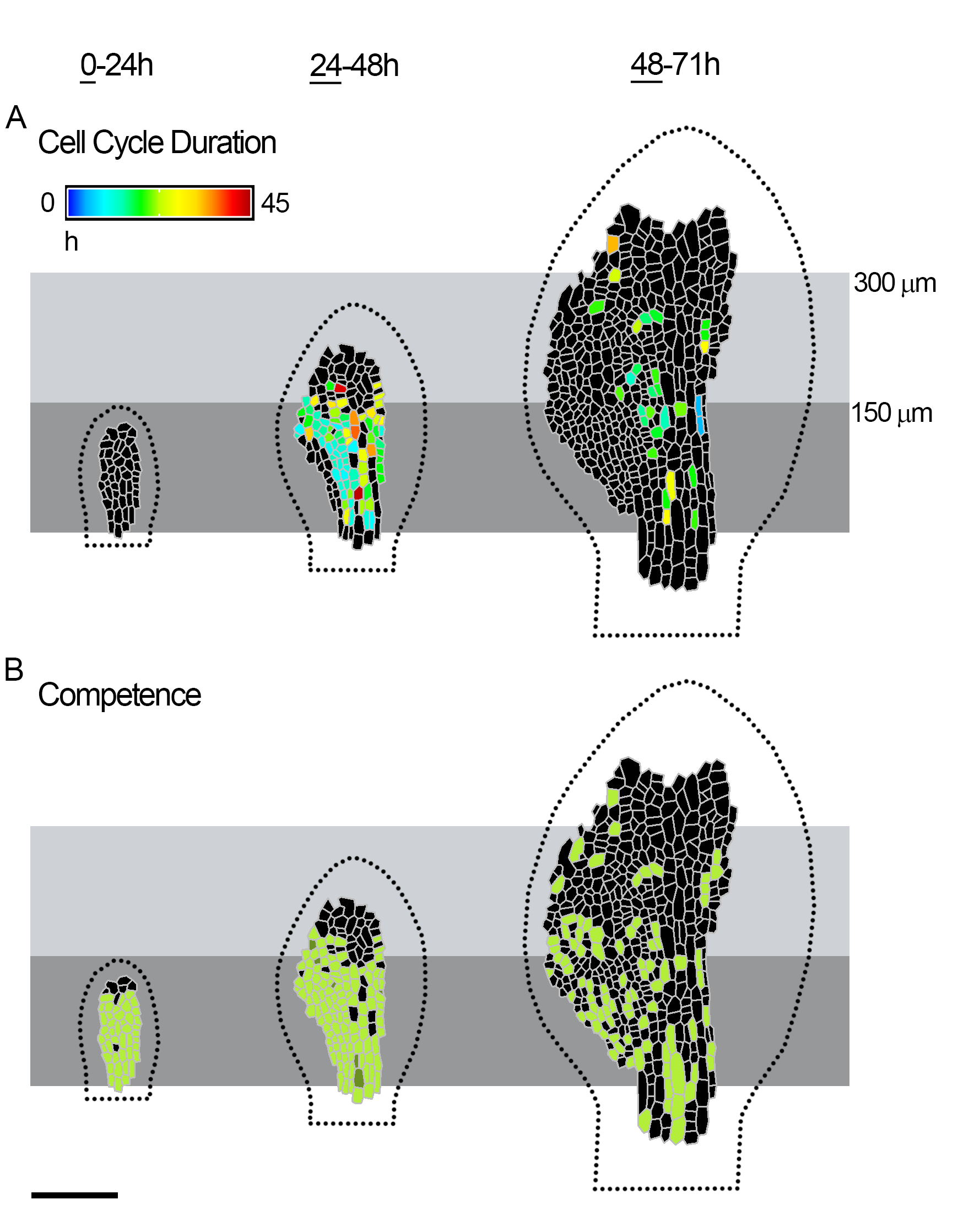

Supplement: S7 Fig — Cells amenable to tracking from the time-lapse imaging experiment shown in Fig 2; data visualised over 24-h intervals, shown on the first image of the interval (underlined). (A) Cell cycle duration (heat map) for cells that were observed to complete a full cell cycle during the course of the experiment. Cells that did not divide or did not complete a full cell cycle are coloured black. (B) Cells that were competent to divide (green) and either executed division during the interval (light green) or divided in a later interval (dark green). Cells that did not divide (black). Leaf outline indicated by dotted black line. The petiole-lamina boundary was defined as described in Fig 1. Grey boxes are aligned to the petiole-lamina boundary and extend to 150 or 300 μm. Scale bar = 100 μm. (TIF) [file pbio.2005952.s007.tif]

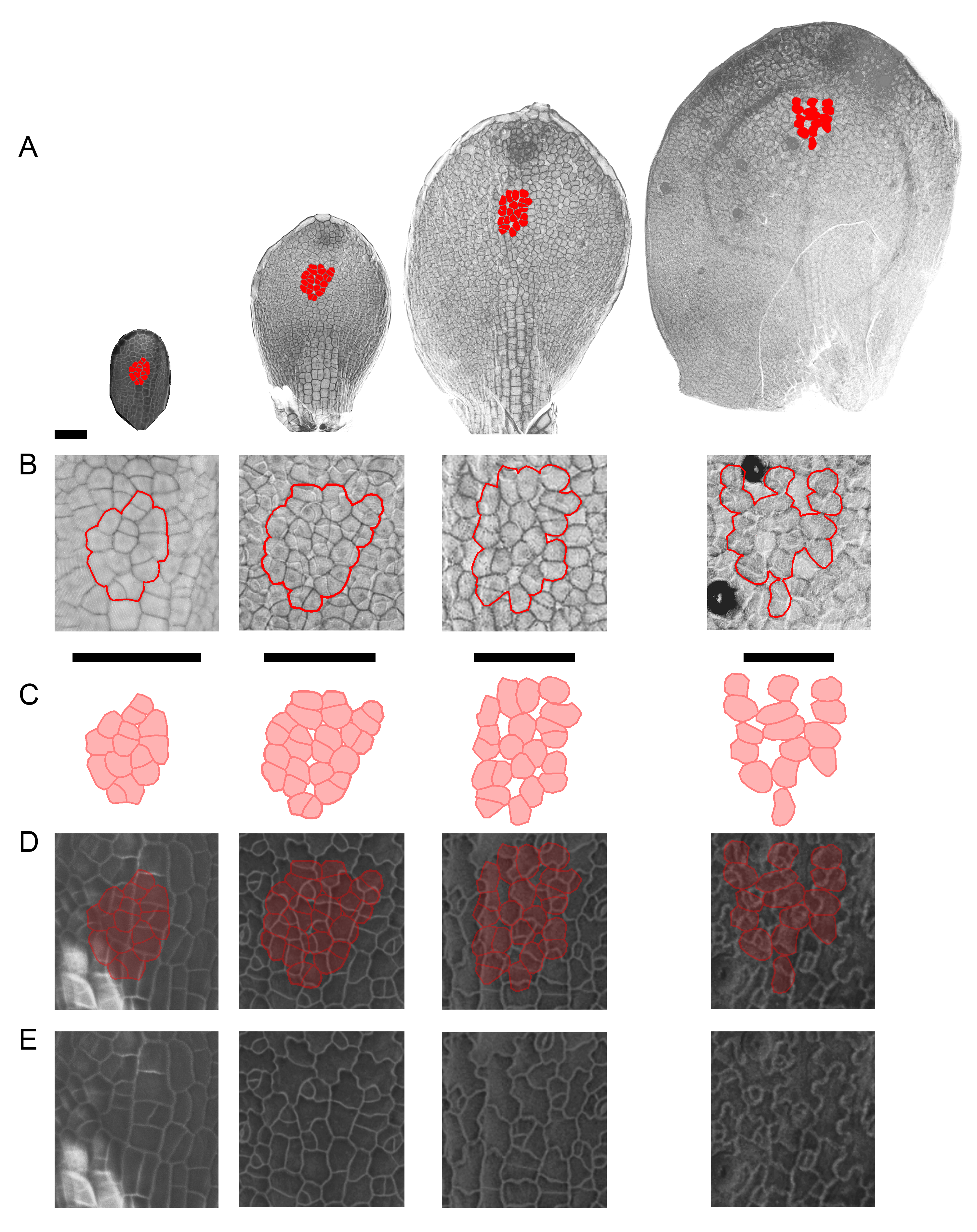

Supplement: S8 Fig — (A) Projections of the subepidermal layer of four individual wild-type leaves, fixed and stained at developmental stages, similar to those of the tracked spch leaf in Fig 5. Leaf widths (left to right) are 0.14, 0.28, 0.40, and 0.53 mm. A patch of cells was coloured red and used to look in further detail. (B) Enlargement of the patch of cells in (A) (red outline). (C) Cells outlined in (B), showing spacing of individual cells (filled pink, outlined red). (D) Epidermal cells adjacent to the subepidermal patch (subepidermal cells filled and outlined red). (E) Epidermal cells without subepidermal patch outlined. Scale bars = 50 μm. Source data are available from https://figshare.com/s/b14c8e6cb1fc5135dd87. spch, speechless. (TIF) [file pbio.2005952.s008.tif]

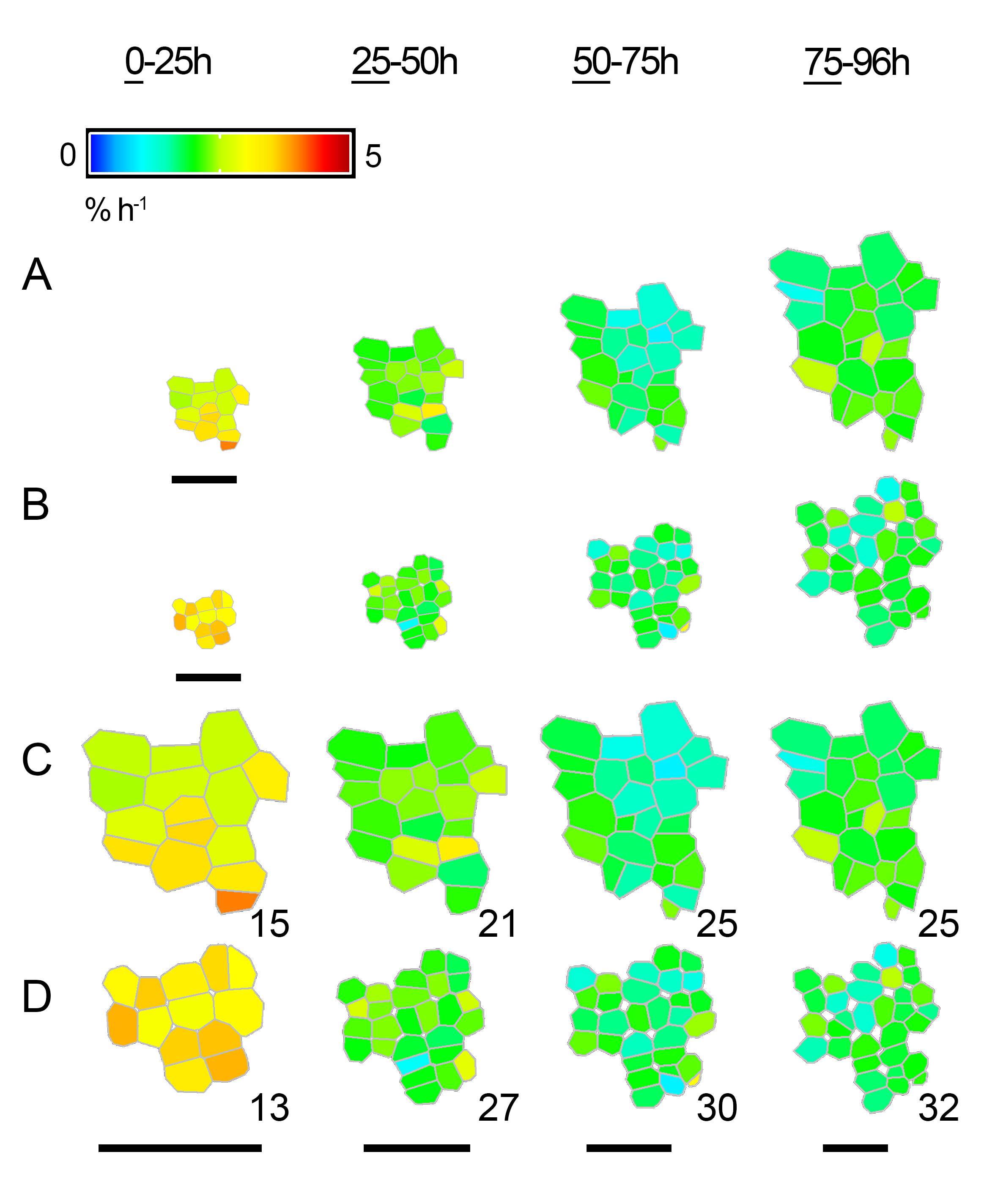

Supplement: S9 Fig — A group of cells from the time-lapse imaging experiment shown in Fig 5, subepidermis (cells highlighted red), and adjacent epidermis (dynamics of the epidermis shown in full in S3 Fig). Average cell areal growth rates for each tracking interval are shown on the first image of each interval (time point underlined). (A) Epidermis, (B) subepidermis. The patch of cells is also shown enlarged for comparison. (C) Epidermis. (D) Subepidermis, showing increase in number of cells through division compared to the epidermis. Numbers refer to number of cells. Scale bars = 50 μm. (TIF) [file pbio.2005952.s009.tif]

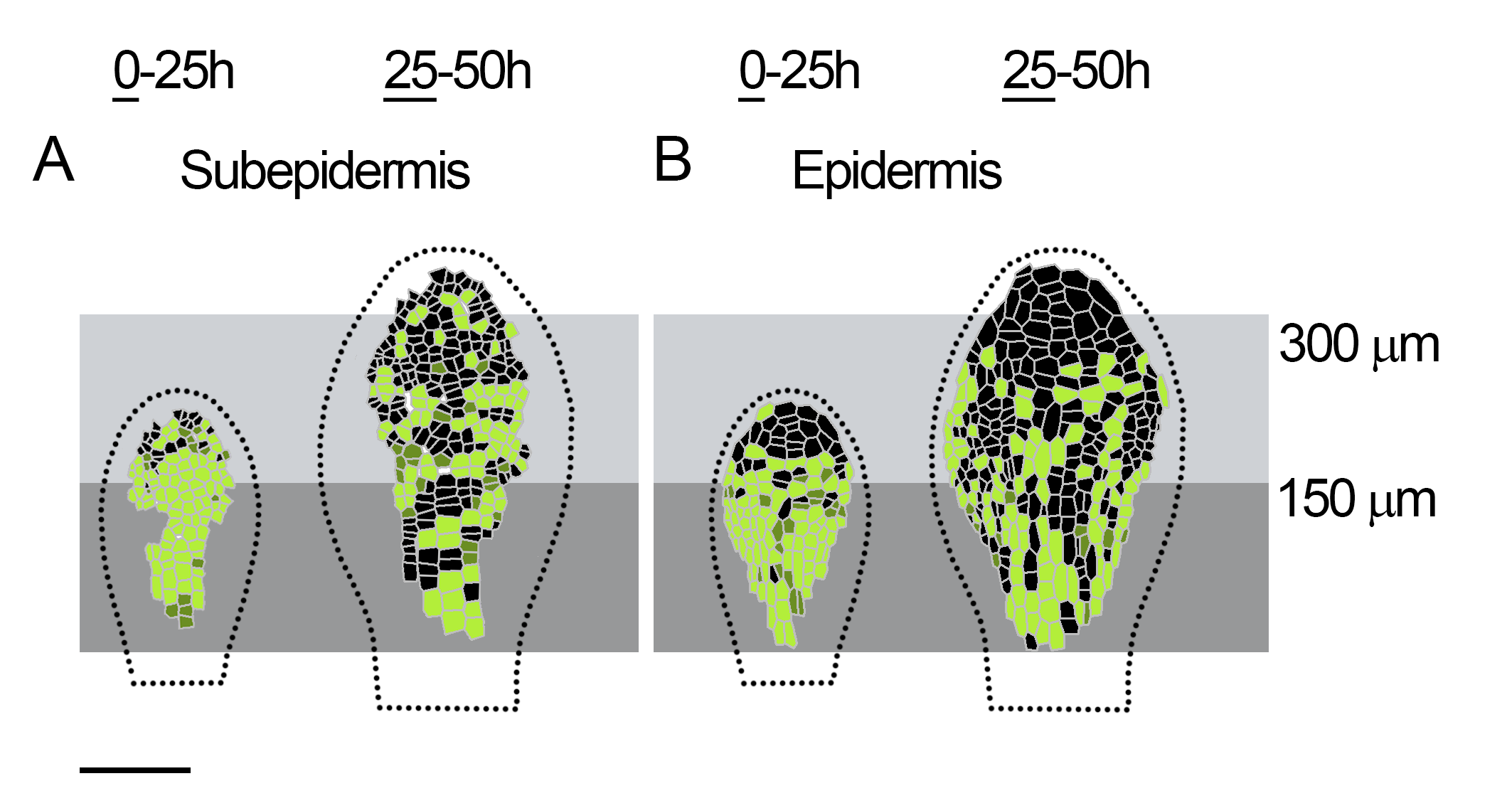

Supplement: S10 Fig — Time-lapse imaging of a spch leaf at approximately 24-h intervals over 2 d (0–50 h); later time points of this experiment are shown in S3 Fig. Cells amenable to tracking are shown on the first time point (underlined). Cells that were competent to divide (green) and either executed division during the interval (light green) or divided in a later interval (dark green). Cells that did not divide (black). (A) Subepidermis. (B) Epidermis. Leaf outline indicated by dotted black line. Leaf widths from left to right are 0.17 and 0.27 mm. The petiole-lamina boundary was defined as described in Fig 1. Grey boxes are aligned to the petiole-lamina boundary and extend to 150 or 300 μm. Scale bar = 100 μm. spch, speechless. (TIF) [file pbio.2005952.s010.tif]

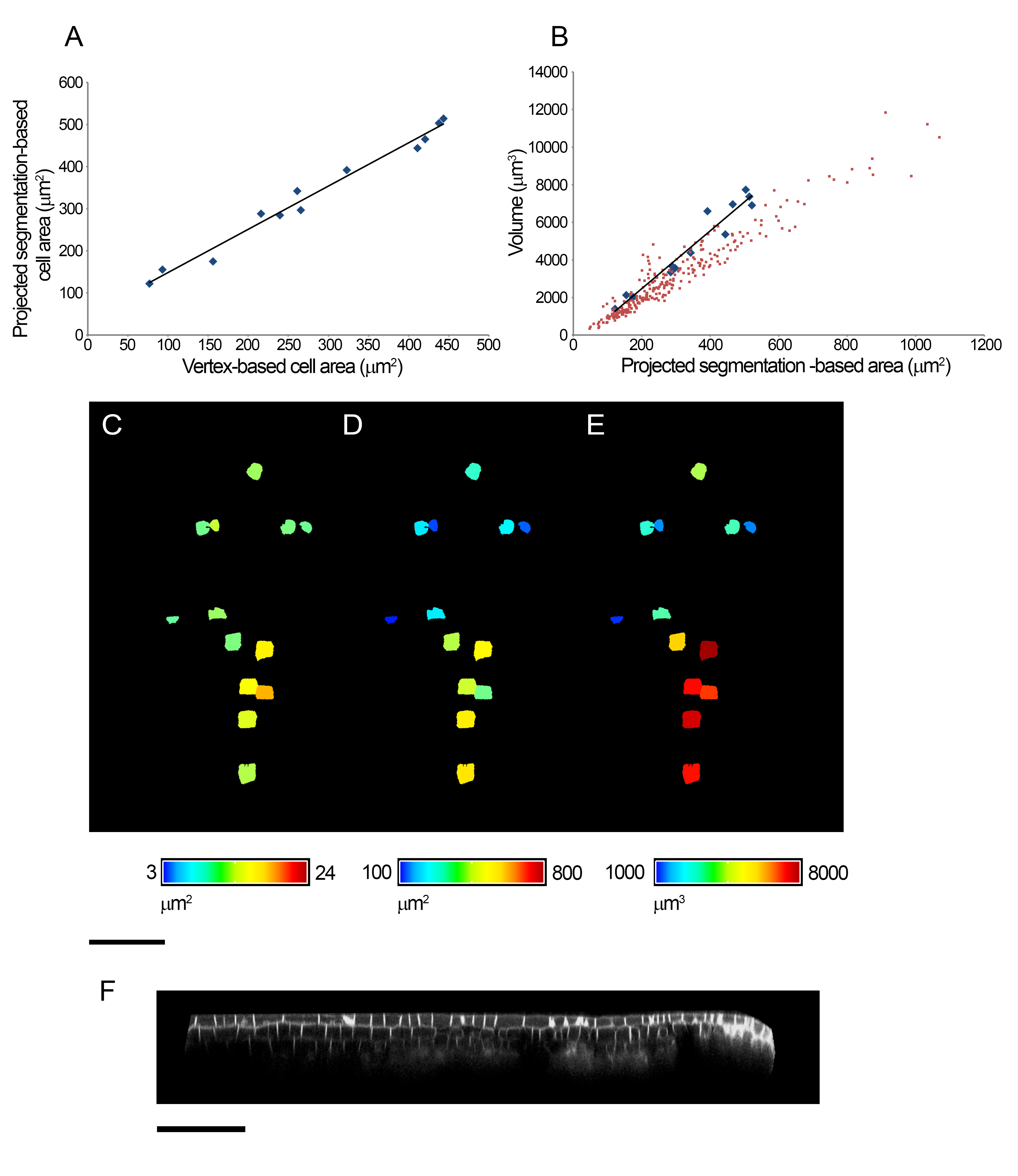

Supplement: S11 Fig — Analysis of a sample of abaxial subepidermal cells from the tracking experiment shown in Fig 6, 58 h. (A) Projected segmentation-based cell area versus vertex-based area (R2 = 0.98, slope = 1.03, intercept = 46, standard deviation along y-axis = 20). (B) Cell volume versus projected segmentation-based cell area, subepidermal cells (blue, R2 = 0.94, slope = 15.4), epidermal cells (red). (C) Cell volume divided by projected segmentation-based area (approximate cell thickness). (D) Projected segmentation-based area. (E) Cell volume. For each heat map, the upper limit was set to 8-fold that of the lower limit. (F) Orthogonal slice of confocal image, approximately through the midline of the leaf. Thickness of epidermal and subepidermal cells appears approximately uniform from leaf base (left) to leaf tip (right). Subepidermal cells close to the distal leaf tip are difficult to resolve. Scale bars = 100 μm. Source data are available from https://figshare.com/s/b14c8e6cb1fc5135dd87. (TIF) [file pbio.2005952.s011.tif]

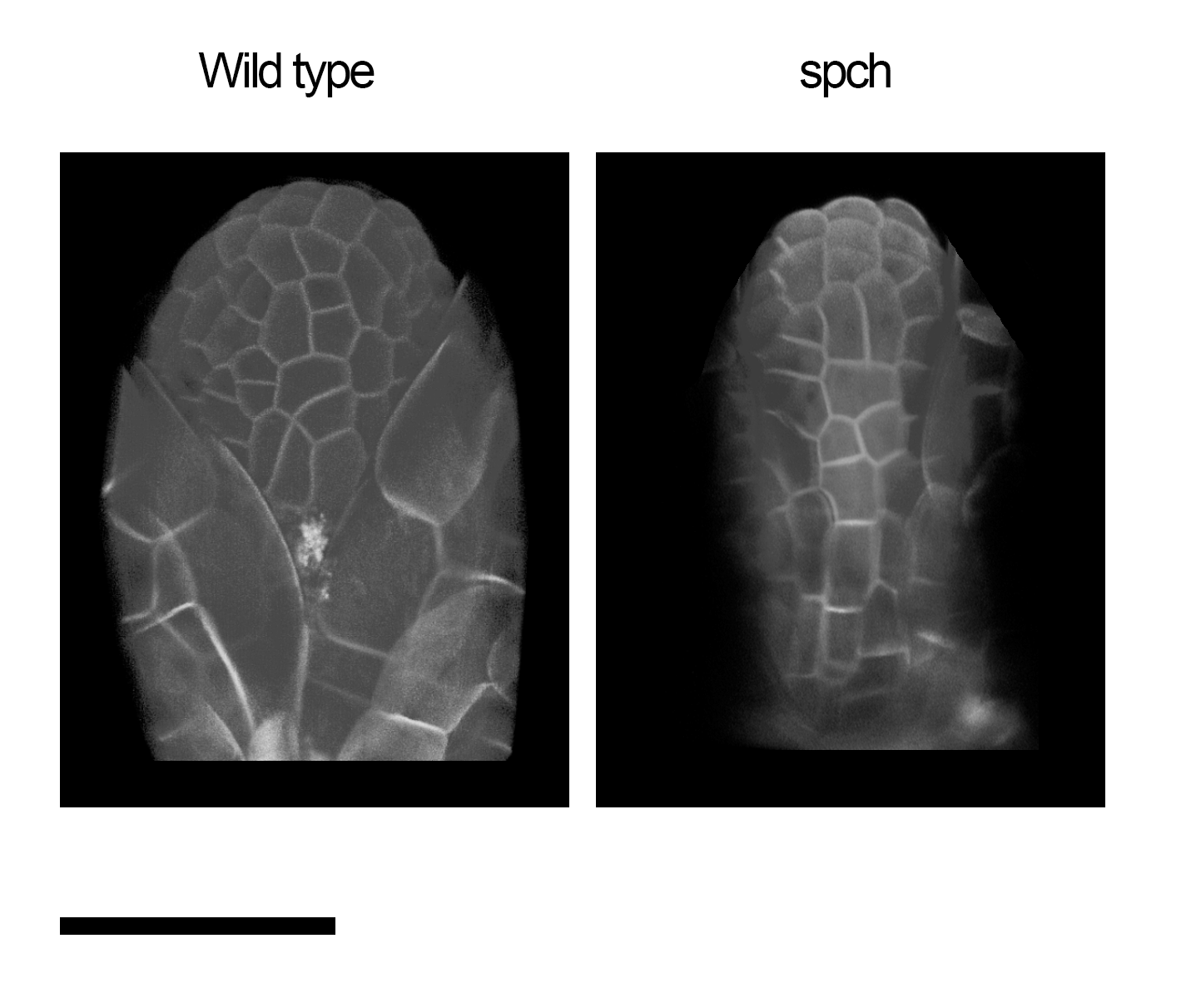

Supplement: S12 Fig — Confocal images of an early wild-type leaf primordium (left) and a spch primordium (right) at similar developmental stages. The leaf primordia are partially obscured by the petioles of cotyledon leaves. Scale bar = 50 μm. spch, speechless. (TIF) [file pbio.2005952.s012.tif]

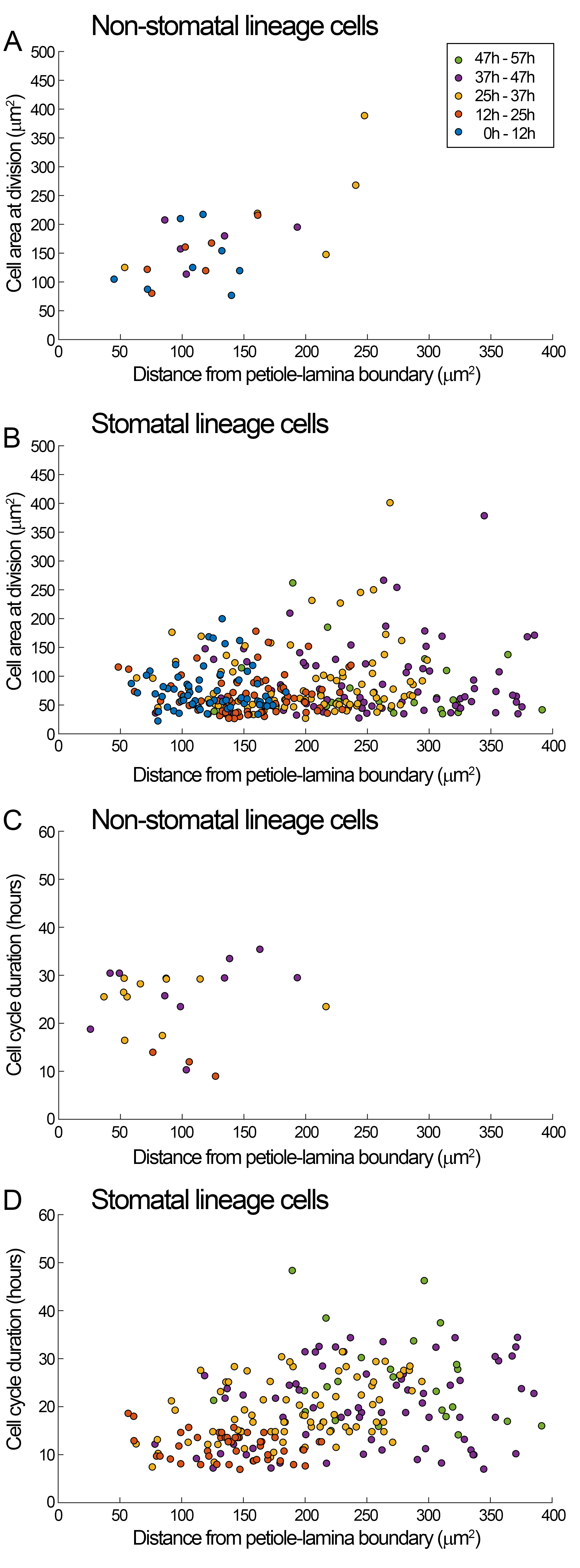

Supplement: S13 Fig — Data from cells amenable to tracking in the time-lapse experiment shown in Fig 7. (A,B) Area of lamina cells at the time of division execution versus distance from the petiole-lamina boundary. Mean cell area at the division for all cells is 87 ± 6.0 μm2. (A) Cells classified as non-stomatal lineage (mean = 165 ± 27.8 μm2). (B) Cells classified as stomatal lineage (mean = 81 ± 5.7 μm2). (C,D) Cell cycle duration for all cells observed to complete a cell cycle. (C) Non-stomatal lineage cells (mean = 24.6 ± 2.8 h). (D) Stomatal lineage cells (mean = 18.5 ± 1.05 h). ± ranges indicate 1.96 × standard error of mean. Data points are colour coded according to time interval (inset in A). Source data are available from https://figshare.com/s/b14c8e6cb1fc5135dd87. (TIF) [file pbio.2005952.s013.tif]

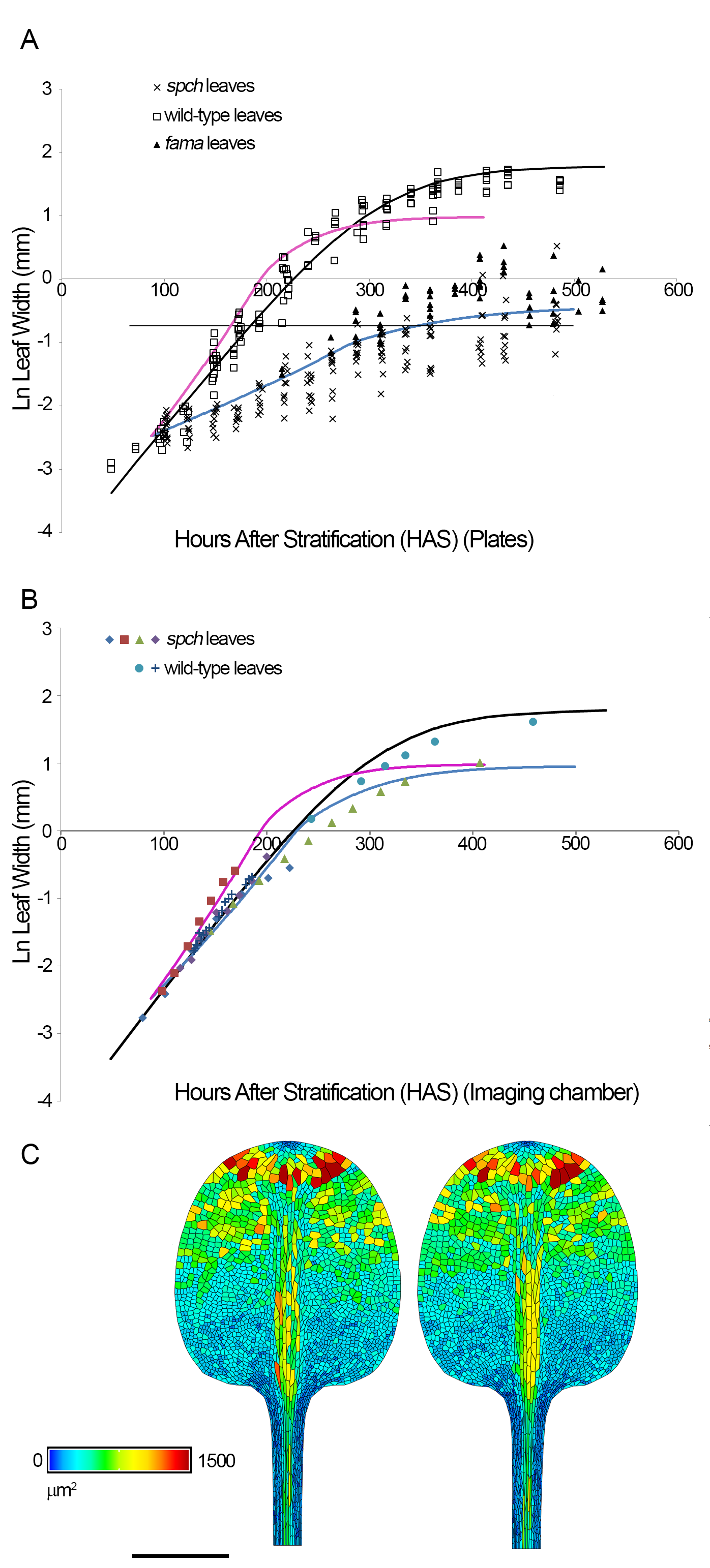

Supplement: S14 Fig — (A) Width measurements of leaf 1 from spch, wild-type, and fama seedlings grown in standard conditions on plates. The fitted growth curve of wild type (solid black line) was based on a logistic calculation [16]. Pink line shows output leaf widths for the model. Blue line shows output leaf widths for the model tuned to match spch growth on plates (by slowing growth by 40% and physiological time by 45%). Horizontal line shows leaf width at about 0.5 mm, corresponding to stages shown in Fig 10. (B) Width measurements of leaf 1 from seedlings grown in the bio-imaging chamber and scanned using confocal microscopy. Measurements are from six independent tracking experiments, two wild-type individuals, and four spch individuals (colour key). Because spch plants grown prior to moving into the chamber have much-reduced growth (shown in B), the initial data point from each tracking experiment was normalised to the wild-type logistic curve (solid black line) to enable subsequent growth rates to be compared. Pink line shows output leaf widths for the model. Blue line shows output for the leaf model tuned to match spch growth in the chamber at later stages (by slowing physiological time by 25%). (C) Outputs for the final stage comparing the model (left) with that tuned to match spch growth in chamber (right). Source data are available from https://figshare.com/s/b14c8e6cb1fc5135dd87. spch, speechless. (TIF) [file pbio.2005952.s014.tif]

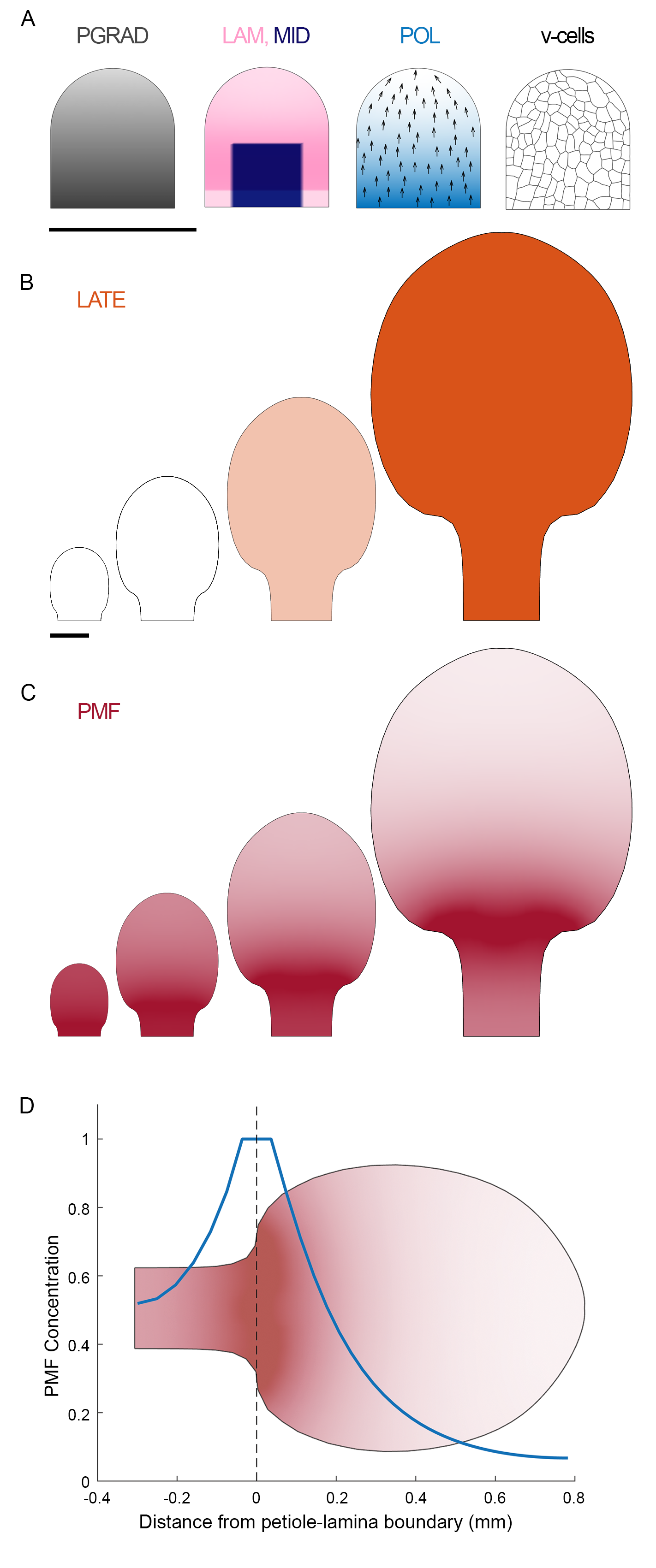

Supplement: S15 Fig — (A) Initial canvas distribution of regulatory factors, from left to right: PGRAD (greyscale), LAM (pink) and MID (purple), POL (blue, with arrows indicating gradient), and initial pattern of v-cells. (B) Time series showing accumulation of LATE at 115, 124, 153, and 182 h. (C) Time series of PMF levels at 115, 124, 153, and 182 h. (D) Sample plot of PMF concentration along the leaf midline. Note PMF concentration is fixed at 1 around the petiole-lamina boundary (dotted line). LAM, a factor distinguishing lamina from petiole; LATE, a timing factor; MID, a mediolateral factor; PGRAD, a graded proximodistal factor; PMF, proximal mobile factor; POL, factor determining polarity field; v-cell, virtual cell. (TIF) [file pbio.2005952.s015.tif]

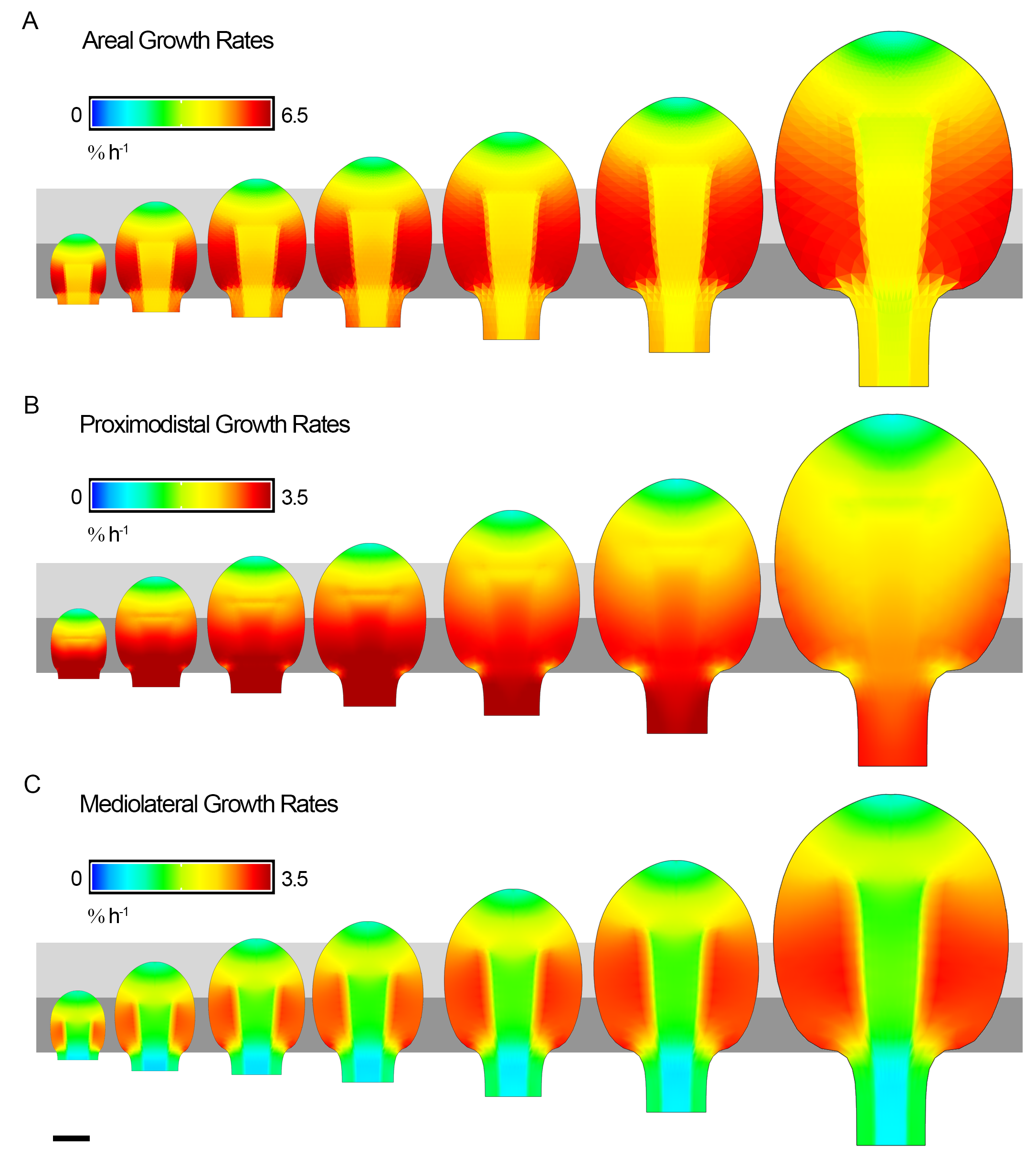

Supplement: S16 Fig — Model output (from left to right) at 115, 132, 140, 147, 156, 164, and 178 h showing (A) resultant areal growth rates, (B) resultant growth rates parallel to the midline (proximodistal growth rates), and (C) resultant growth rates perpendicular to the midline (mediolateral growth rates). Grey boxes are aligned to the petiole-lamina boundary and extend to 150 or 300 μm. Scale bar = 100 μm. (TIF) [file pbio.2005952.s016.tif]

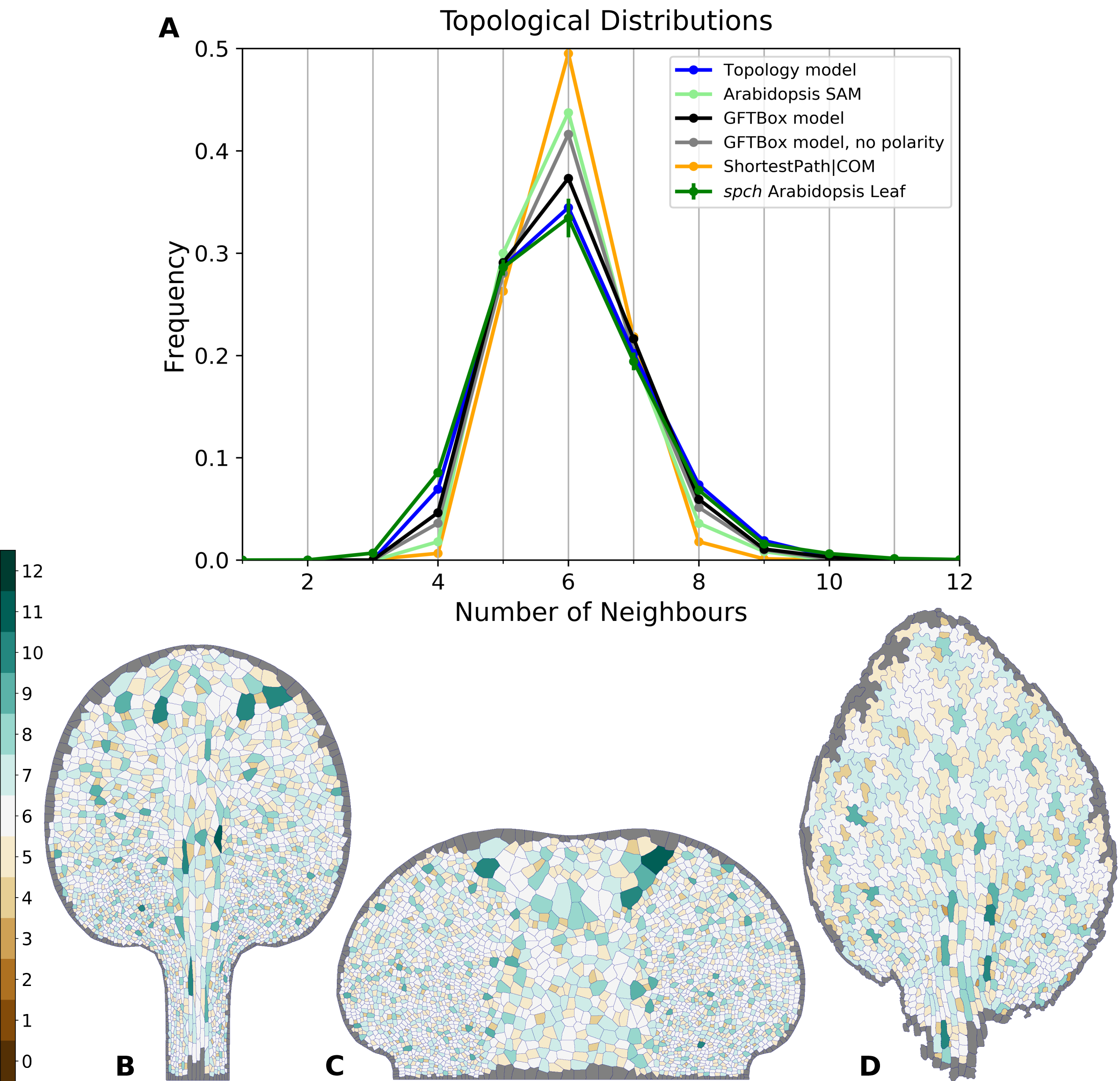

Supplement: S17 Fig — (A) Dark green line shows Arabidopsis spch epidermis with standard deviation between leaves for the fraction of n-sided cells indicated by a vertical line. Data are based on 42,901 cells from 99 spch leaves at different stages of development. The frequency of six-sided cells is 0.334 ± 0.019 (SD). Black line shows the result of the model of spch epidermis presented here. Grey line shows the model without polarity (isotropic specified growth). Light green line shows empirical data from the shoot apical meristem [31]. Orange line shows the model using the shortest path through the cell’s centroid within an isotropically growing tissue [31]. Dark blue line shows a graph-model in which cells divide through the topological centre [30]. (B-D) Tissue topology with cells coloured according to their neighbourhood number, with six-sided cells in white, higher numbers in green, and lower numbers in brown, as shown in the colour scale. (B) Result for the model of spch epidermis presented here (black profile in A); (C) model without polarity, yielding an isotropically growing tissue (grey profile in A). (D) Example of spch leaf, as shown in S18D Fig. Source data are available from https://figshare.com/s/b14c8e6cb1fc5135dd87. spch, speechless. (TIF) [file pbio.2005952.s017.tif]

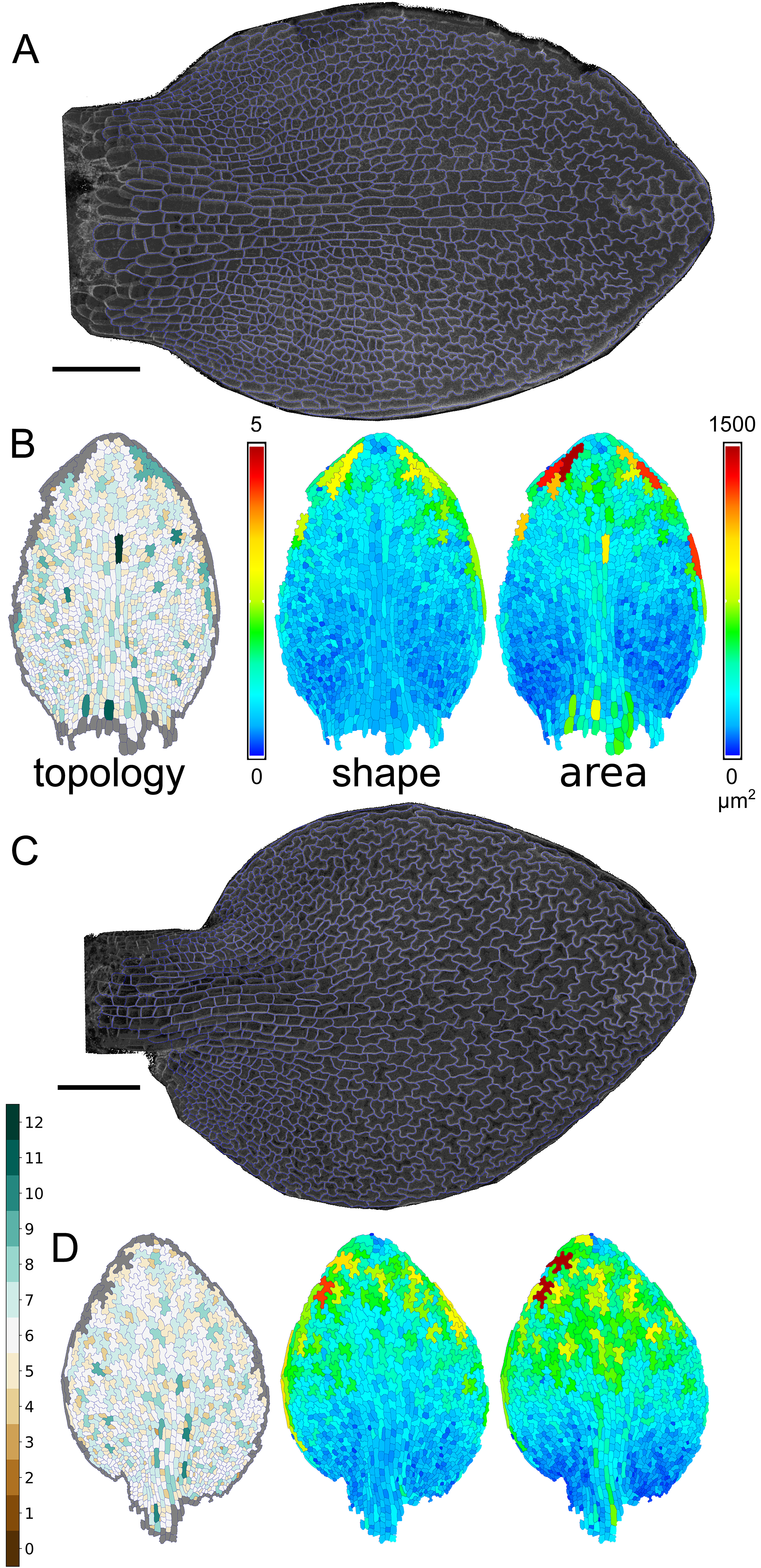

Supplement: S18 Fig — Supporting data for Fig 10; spch leaf grown in the chamber at 8 DAS (A-B) or grown on plates at 13 DAS (C-D) at similar leaf widths to leaves presented in Fig 10. (A, C) Confocal images of leaves, with segmentation outlines overlain in blue. (B, D) Analysis of segmentations in (A, C) showing (from left to right) neighbourhood numbers (following neighbourhood colour map used in S18B Fig); cell complexity based on the CD measure using LOCO-EFA (using complexity colour scale as in Fig 10K–10N); and absolute areas (using colour scale as in Fig 10A and 10B). Results are consistent with those described for Fig 10. Scale bars = 100 μm. Source data are available from https://figshare.com/s/b14c8e6cb1fc5135dd87. CD, cumulative difference; LOCO-EFA, Lobe-Contribution Elliptic Fourier Analysis; spch, speechless. (TIF) [file pbio.2005952.s018.tif]
